# Maleate salts of bedaquiline

**DOI:** 10.1107/S2056989021002991

**Published:** 2021-03-26

**Authors:** Matthias Zeller, Susan Bogdanowich-Knipp, Pamela Smith, Dale K. Purcell, Mercy Okezue, Daniel T. Smith, Stephen R. Byrn, Kari L. Clase

**Affiliations:** a Department of Chemistry, Purdue University, 560 Oval Dr., W. Lafayette, IN 47907-2084, USA; b Ravine Pharmaceuticals LLC, 3425 DuBois St., West Lafayette, IN 47906, USA; c Leading with Smart Science LLC, 5315 Shootingstar Ln, West Lafayette, IN 47906, USA; d Chemical Microscopy LLC, 1281 Win Hentschel Blvd., West Lafayette, IN 47906, USA; e Biotechnology Innovation and Regulatory Science Center, Lilly Hall of Life Sciences, 915 State Street, Purdue University, West Lafayette, IN 47906, USA; f Agricultural & Biological Engineering, Purdue University, 225 South University Street, West Lafayette, IN 47907, USA; g Industrial and Physical Pharmacy, Purdue University, 575 Stadium Mall, West Lafayette, IN 47906, USA; h Improved Pharma LLC, 1281 Win Hentschel Blvd. Suite 1565, West Lafayette, IN 47906, USA

**Keywords:** bedaquiline, drug-resistant tuberculosis, isomorphous organic salts, desolvation, crystal structure

## Abstract

Bedaquiline is one of two important new drugs for the treatment of drug-resistant tuberculosis (TB). It is marketed in the US as its fumarate salt, but only a few salts of bedaquiline have been structurally described so far. We present here five crystal structures of bedaquilinium maleate {systematic name: [4-(6-bromo-2-meth­oxy­quinolin-3-yl)-3-hy­droxy-3-(naphthalen-1-yl)-4-phenyl­but­yl]di­methyl­aza­nium 3-carb­oxy­prop-2-enoate}, C_32_H_32_BrN_2_O_2_^+^·C_4_H_3_O_4_^−^, namely, a hemihydrate, a tetra­hydro­furan (THF) solvate, a mixed acetone/hexane solvate, an ethyl acetate solvate, and a solvate-free structure obtained from the acetone/hexane solvate by *in situ* single-crystal-to-single-crystal desolvation. All salts exhibit a 1:1 cation-to-anion ratio, with the anion present as monoanionic hydro­maleate and a singly protonated bedaquilinium cation. The maleate exhibits the strong intra­molecular hydrogen bond typical for *cis*-di­carb­oxy­lic acid anions. The conformations of the cations and packing inter­actions in the maleate salts are compared to those of free base bedaquiline and other bedaquilinium salts.

## Abstract 2

The single-crystal structures of several maleate salt of bedaquiline, a drug used for the treatment of drug-resistant tuberculosis (TB), are described.

## Chemical context   

Bedaquiline is one of two important new drugs for the treatment of drug-resistant tuberculosis (TB). It is marketed in the US as the fumarate salt with the trade name SirturoTM (Brigden *et al.*, 2015 ▸) and described in US Patent 8,546,428 (Hegyi *et al.*, 2013 ▸). A number of other bedaquilinium salts have been reported since the emergence of its pharmacolog­ical relevance, but until recently only free base bedaquiline had been fully structurally described (Petit *et al.*, 2007 ▸). To fill this gap, which severely hampers understanding of the chemical, physical and physiological properties of bedaquiline and its derivatives, we have recently reported and analyzed the single-crystal structures of several bedaquilinium salts, including that of the commercially traded fumarate as well as two differently solvated benzoate salts (Okezue *et al.*, 2020 ▸). This study revealed that bedaquiline and its salts have a very rich and diverse structural chemistry. Depending on the nature of the anion (fumarate, benzoate or none for free base bedaquiline), different mol­ecular conformations and structural motifs are observed. In free base bedaquiline, the amine moiety is engaged in an intra­molecular O—H⋯N hydrogen bond, limiting the formation of inter­molecular hydrogen-bonding inter­actions. The packing is instead dominated by weaker and less directional inter­actions such as Br⋯Br inter­actions and π-stacking (Petit *et al.*, 2007 ▸). In the fumarate and benzoate salts, the protonated amine moiety is available as a hydrogen-bond donor and forms bonds with the benzoate or fumarate anions, and these salts are dominated by a multitude of N—H⋯O and O—H⋯O hydrogen-bonding inter­actions that connect the cations and anions into strongly hydrogen-bonded ribbon-like structures. The ethane backbone and the malleable ethyl­amine fragment of the bedaquiline core result in a high degree of flexibility, and mol­ecular conformations vary not only widely between the bedaquiline and bedaquilinium structures, but even between independent mol­ecules within the same structure (both the free base and the fumarate are *Z*′ = 2 structures). For a pharmaceutically relevant material, it is essential that a crystalline material can be obtained in a stable and well-defined form. The formation of solvates is generally undesirable, especially if the incorpor­ated solvent mol­ecules are volatile or not generally recognized as safe (GRAS) for human consumption. For the bedaquilinum system, the pronounced conformational flexibility makes any predictions about how a bedaquilinium anion pair might crystallize, and whether solvates are formed and of which kind, extremely difficult. *In silico* crystal-structure prediction, even if only intended as a screening to narrow down a list of anion and solvent candidates, is not yet a viable option for this system; therefore, the best recourse for the bedaquiline system remains experimental screening of combinations of anions, solvents and crystallization conditions to establish which combinations will yield stable and well-defined crystal forms for potential use in pharmaceutical formulations. To this end, we have investigated the combination of bedaquiline with maleate as the anion. *Via* screening of solvents and crystallization conditions, we were able to establish the structures of several of its solvates: a hemihydrate, a THF solvate, an acetone/hexane solvate and an ethyl acetate solvate. A solvate-free form obtained by *in situ* desolvation of the acetone/hexane solvate will also be described. The influence of the incorporated solvents on the crystal structures and their stability will be discussed.
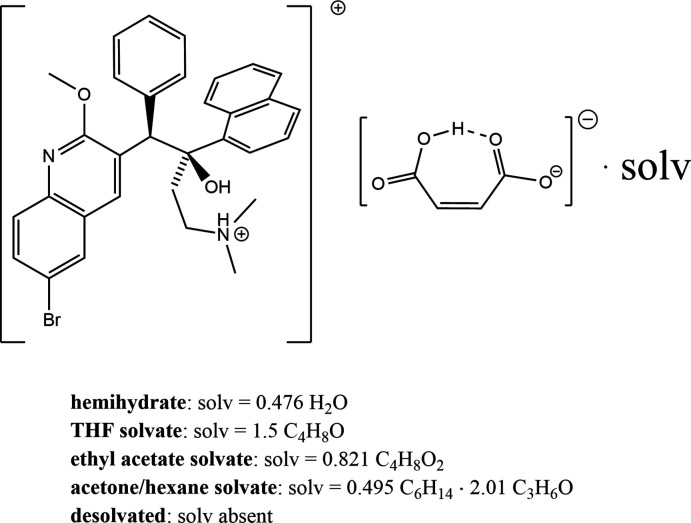


## Structural commentary   

**Figure 1:**
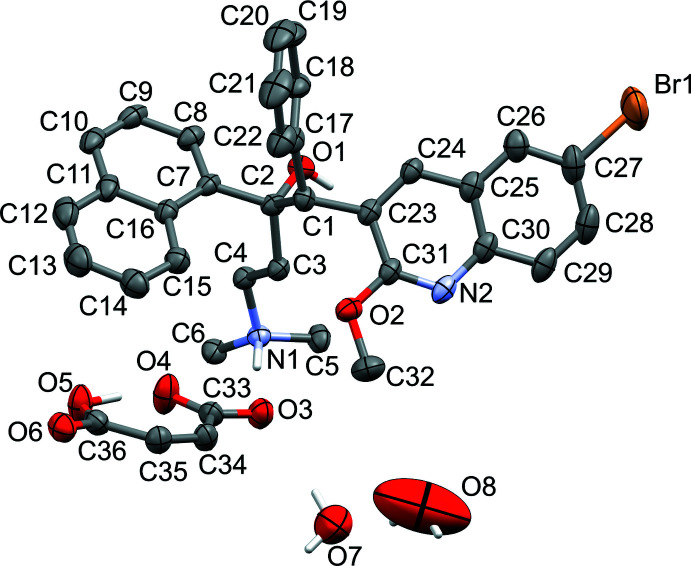
Probability ellipsoid plot (50% probability) of the hemihydrate. C-bound H atoms and H-atom labels are omitted for clarity. Water mol­ecules O7 and O8 are partially occupied [0.276 (17) for O7 and 0.40 (4) for O8].

Probability ellipsoid plots with selected atom labels and solvate mol­ecules (where present) are shown in Figs. 1 ▸–5 ▸ ▸ ▸ ▸. The atom-naming scheme was adopted from the one used for the fumarate and benzoate structures and used for all solvates. Refinement details, including disorder refinement strategies (where present) are given in the *Refinement* section.

Similar to the other three bedaquilinum salts reported thus far, the maleate salt features a singly protonated bedaquilin­ium cation with a 1:1 anion-to-cation ratio. Like in the fumarate and benzoate salts, the protonation site is the dimethyl amine fragment. The second basic site, the quinoline nitro­gen atoms, remained unprotonated, in agreement with the second p*K_a_* of maleic acid (6.22, European Chemical Agency, 2015 ▸), which is not sufficiently basic for a proton transfer to this site. The first p*K_a_* of maleic acid (1.94, European Chemical Agency, 2015 ▸) should be sufficient to protonate the quinoline site if higher ratios of maleic acid to bedaquiline are used (Okezue *et al.*, 2020 ▸). We were, however, unable to identify or isolate any different crystalline materials when increasing the amounts of acid (screening was done by powder XRD).

The four bedaquilinium maleate structures presented here were found to be isomorphous or nearly isomorphous, differing mostly only in the nature of the incorporated solvate mol­ecules. One of the structures, the ethyl acetate solvate, also shows a pronounced modulation of the bedaquilinium maleate, leading to breaking of the crystallographic symmetry observed for the other structures (see the *Supra­molecular features* section for a detailed discussion). Similar isomorphous structures were also found for samples obtained from other solvent systems such as iso­propanol or *n*-propanol, as evidenced by their powder XRD patterns. However, no single crystals of high enough quality for a full structural analysis could be obtained thus far.

The ethane backbone and the malleable ethyl­amine fragment gives the bedaquilinium cation a high degree of flexibility that allows the cations to respond readily to crystal-packing forces. In the previously reported structures, the conformations did vary widely not only from structure to structure, but even between independent mol­ecules within the same structure (both the free base and the fumarate are *Z*′ = 2 structures). For these structures, the torsion angles involving the ethyl­amine fragment adopted conformations ranging between *gauche* and *trans* (Table 1 ▸), with the observation for *gauche* found mostly for free base bedaquiline, where it was induced by an intra­molecular O—H⋯N hydrogen bond. However, one of the two mol­ecules in the fumarate salt also featured a single *gauche* angle (for the C1—C2—C3—C4 torsion angle), and two angles in between *gauche* and *trans* [C2—C3—C4—N1 for the two fumarate mol­ecules, with values of 137.2 (2) and 133.7 (2)°, respectively]. All other torsion angles involving the ethyl­amine group adopted *trans* conformations with various degrees of slight distortions, ranging from 164.04 (15) to 178.8 (3)°. The maleate salts follow the same trend. The torsion angles are all slightly distorted *trans* and range from −164.4 (7) to 176.4 (3)° (C17—C1—C2—C3 and C1—C2—C3—C4 angles, extreme values are for each one of the two mol­ecules of the ethyl acetate solvate).

The other free variables that determine the overall mol­ecular structure of the cations are the torsion angles between the rigid planes of bedaquiline, *i.e*. the 6-bromo-2-meth­oxy­quinoline, phenyl and naphthyl planes (Table 1 ▸). Variations of only a few degrees can be seen between equivalent angles of the various inter­planar angles in the cations, as would be expected for mostly isomorphous structures. The 6-bromo-2-meth­oxy­quinoline *vs* phenyl angle ranges from 63.0 (2) to 71.31 (7)°, which is slightly smaller but similar to what was observed in the previously reported structures [73.29 (7) to 86.02 (8)°; Petit *et al.*, 2007 ▸; Okezue *et al.*, 2020 ▸]. Phenyl to naphthyl angles are between 63.7 (1) and 66.60 (7)°. Previously reported values span a much wider range, from 44.2 (1) to 89.74 (9)°. The bromo-2-meth­oxy­quinoline *vs* naphthyl angles are between 26.4 (1) and 32.62 (9)°, compared to 8.16 (9) to 37.50 (6)° for the other known bedaquiline structures.

**Figure 6:**
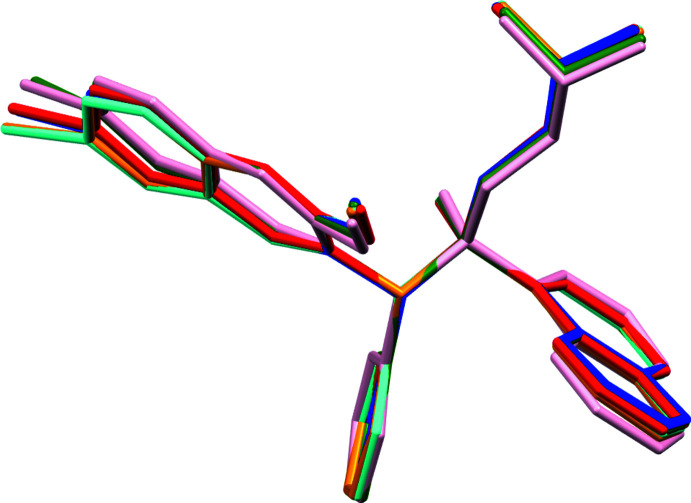
Least-squares overlay based on the atoms C1, C2, C7, C17 and C23. Color coding: hemihydrate – orange; THF solvate – green; ethyl acetate solvate – red and pink (two independent mol­ecules); acetone/hexane solvate – blue; desolvated structure – cyan.

Numerical variations between the four structures are thus clearly resolved. They are not, however, large enough to substanti­ally alter the overall shape and appearance of the cations, as can be seen in a least-squares overlay based on the atoms C1, C2, C7, C17 and C23 around the center of the cation (Fig. 6 ▸). The structures clearly still have the same overall conformation, just slightly modulated by inter­actions with solvate mol­ecules and small differences in unit cell dimensions. The largest variations in the overlay can be seen for the outer atoms of the 6-bromo-2-meth­oxy­quinoline plane, especially the bromine atom, the meth­oxy group, the outer atoms of the naphthyl group, and to a lesser degree for the dimethyl ammonium fragment.

## Supra­molecular features   

Packing and inter­molecular inter­actions not involving solvate mol­ecules are essentially identical between the four structures, a virtue of their isomorphous or nearly isomorphous nature. Unless stated otherwise, all distances in the following discussion will be those of the hemihydrate structure.

**Figure 7:**
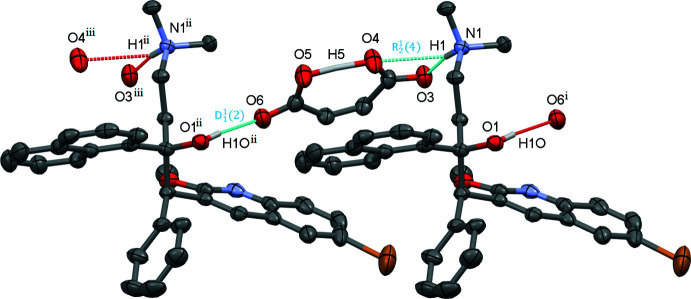
The main hydrogen-bonding inter­actions in common to all structures (turquoise and red dashed lines). Shown is the hemihydrate. Partially occupied water mol­ecules, C-bound H atoms and labels for C-bound H atoms are omitted for clarity. Probability ellipsoids are at the 50% level. Symmetry codes: (i) −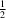 + *x*, −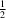 + *y*, *z;* (ii) 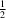 + *x*, 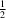 + *y*, *z;* (iii) 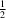 + *x*, 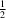 + *y*, *z*.

The main directional forces that are involved in stabilizing crystals of bedaquilinium maleate are hydrogen bonds (Tables 2 ▸–7 ▸ ▸ ▸ ▸ ▸) and π–π stacking inter­actions. One hydrogen bond is intra­molecular and connects the carb­oxy­lic acid and carboxyl­ate groups of the hydro­maleate anion, which shows the very strong and close to symmetrical hydrogen bonding typical for *cis*-di­carb­oxy­lic acid anions (Fig. 7 ▸). The acidic maleate hydrogen atoms are well resolved in all five structures and their positions were freely refined. The position of the H atom varies slightly between the five structures. It is close to symmetric, with a slight deviation towards oxygen atom O5 in all but the THF solvate (see Table 8 ▸ for numerical details). The more accurately measured carbon–oxygen bond distances confirm the slight asymmetry for the hydro­maleate, with the C33—O4 bond being on average 0.02 Å shorter than the C36—O5 bond. This includes the THF solvate, for which the H atom was found slightly closer to O4, indicating that the H-atom position is not measured sufficiently accurately to reliably determine its actual position (among the five structures, the THF solvate has the largest estimated standard deviations for atom positions, and within its s.u., the position of H5 is symmetric between O4 and O5).

The not quite symmetric nature of the hydro­maleate anion could be a result of asymmetric inter­molecular hydrogen bonding towards the two ends of the anion. Oxygen atom O3 acts as a hydrogen-bond acceptor towards the ammonium cation, while O6 plays the same role for the hydroxyl group of another cation (at 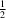 + *x*, 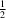 + *y*, +*z*). The N—H⋯O hydrogen bond, being charge assisted, is slightly shorter and stronger than its O—H⋯O equivalent on the other side of the anion, inducing the negative charge of the anion to be localized more on the O3/O4 carboxyl­ate group, and the positive proton being slightly delocalized towards O5.

The individual graph-set motifs (Etter *et al.*, 1990 ▸) for the inter­molecular O—H⋯O and N^+^—H⋯O hydrogen-bonding inter­actions common to all structures are as follows (Fig. 7 ▸): a linear *D* ^1^_1_(2) motif for the O—H⋯O hydrogen bond with one hydrogen-bond acceptor and one hydrogen-bond donor and a bifurcated 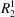
(4) motif towards both O3 and O4 of the maleate for the N^+^—H⋯O bond. The latter inter­action is, however, quite asymmetric, with the N1⋯O3 distance substanti­ally shorter than the N1⋯O4 distance [2.707 (4) and 3.174 (4) Å in the hemihydrate structure], thus making a description as *D* ^1^_1_(2) more suitable. Together, the N—H⋯O and O—H⋯O hydrogen bonds connect the cations and anions into infinite chains. The graph-set motif of these chains is 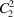
(13). This means that including the connecting carbon atoms of the maleate and the propyl­ene backbone of the beadquilinium cation expands the graph-set motif from individual linear *D* hydrogen bonds into infinite 1D chains with a repeat unit that includes two hydrogen-bond acceptors, two donors and thirteen atoms in total (seven carbon atoms in addition to the N—H⋯O and O—H⋯O moieties), thus 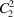
(13).

**Figure 8:**
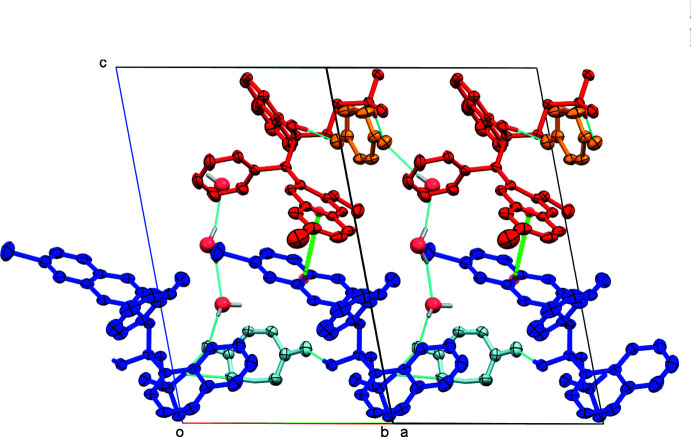
Packing view of the hemihydrate structure showing the propagation directions of the hydrogen-bonded chains. In the lower half of the unit cell, chains propagate horizontally (right–left, along [110]; in the upper half they propagate longitudinally (forward–backward, along [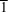
10]). Hydrogen bonds are shown as turquoise dashed lines. Green dashed lines connect the centroids of the bromo­quinoline substituents [3.432 (14) Å]. Partially occupied water mol­ecules shown as spheres of arbitrary radius. For all other atoms, probability ellipsoids are at the 50% level. C-bound H atoms and labels for C H atoms are omitted for clarity.

The 1D chains formed in that way extend diagonally through the lattice along the [110] and the [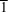
10] directions. Neighboring chains thus do not run in parallel, but are split into chains with two different propagation directions, related to each other by the twofold rotation of the *C*2 space group (red and blue chains in Fig. 8 ▸). Cations and anions along the chain are related to each other *via* half-unit translations of the *C*-centered cell (±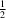 + *x*, ±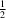 + *y*, +*z*). This differentiates the maleate structures described here from the other previously described bedaquilinium salt structures, the fumarate and benzoate structures, in which hydrogen-bonding inter­actions between cations and anions led to formation of layered structures (Okezue *et al.*, 2020 ▸). For highly solvated structures such as the maleate salts described here, the formation of 1D rather than 2D structures can be of relevance for the resilience of the lattice upon removal of solvent, or the persistence of the packing motif if a different solvent is used. Inter­actions within the layers or chains, mediated *via* hydrogen bonds, are likely to be strong and persistent. The stability of the entire lattice thus depends on how these layers or chains are connected with each other. Are they tightly inter­woven or connected in other ways to ensure stability of the lattice after removal or exchange of solvate mol­ecules? Or can layers or chains easily move past each other, thus allowing easy movement of the secondary building units and either collapse or undergo a complete rearrangement of the entire structure?

In the maleate salts, inter­actions between individual chains is facilitated through effective inter­locking of neighboring chains as well as a number of directional inter­actions, such as C—H⋯O, C—H⋯N and C—H⋯π inter­actions. Importantly, neighboring chains that are rotated against each other by the twofold axis are inter­digitating with each other *via* π–π stacking inter­actions of the bromo­quinoline rings, preventing easy slippage of chains against each other. The stacked quinoline rings are thus related to each other through a twofold rotation (1 − *x*, +*y*, 1 − *z*). They are not exactly coplanar but their planes are angled against each other by 19.43 (8)°. As a result, no exact inter­planar distance can be defined, but the 3.432 (14) Å centroid-to-centroid distance between the quinoline rings (measured for the hemihydrate) indicates an efficient stacking inter­action. The closest atom-to-atom distance is 3.252 (7) Å for the two atoms C30 related by the twofold axis.

Additional weaker inter­actions within the 1D chains and between parallel chains as well as chains that are inclined with respect to each other are provided by C—H⋯O and C—H⋯N inter­actions involving the quinoline nitro­gen and several of the hydro­maleate oxygen atoms as well as by several C—H⋯π inter­actions towards the naphthyl and quinoline π-systems. The *para* C—H group of the phenyl ring forms a C—H⋯N hydrogen bond with the quinoline nitro­gen atom of a neighboring mol­ecule (at 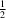 + *x*, −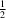 + *y*, +*z*). C—H⋯O bonds towards the maleate O atom O6 are established by both C3 and C6, being the methyl­ene and methyl groups of the dangling dimethyl propyl­ene ammonium group. O6 also acts as the hydrogen-bond acceptor for the hydroxyl O—H⋯O bond, and these C—H⋯O bonds thus just reinforce this connection within the hydrogen-bonded chains, and do not provide any new connection between chains. Atoms O4 and O5 of the maleate, on the other hand, act as C—H⋯O acceptors towards the methyl C6 and naphthyl C10 atoms from cations in neighboring chains, thus providing some stabilization for the overall 3D lattice. Another C—H⋯O inter­action towards O4, originating from another methyl C6 atom, does provide reinforcement for the N—H⋯O bond and no connection between neighboring chains. The meth­oxy and hydroxyl O atoms do not act as acceptors for intra­molecular C—H⋯O bonds. Finally, a number of inter­molecular C—H⋯π inter­actions towards the naphthyl and quinoline π-systems are observed: from naphthyl C14 and maleate C35 towards quinoline density (these inter­actions are within the 1D chains and assist in stabilizing the hydrogen bonds), from phenyl C19 towards maleate C35 (this is an inter-chain inter­action), and a weaker inter­action from methyl C6 towards naphthyl C10 (this is an inter-chain inter­action, but the geometry of this inter­action makes it unlikely to be very stabilizing). The sum of these inter­actions, especially the inter­locking of the stacked quinolones, is likely to prevent slippage of hydrogen-bonded chains against each other, which stabilizes the three-dimensional arrangement against collapse, even upon complete removal of all solvate mol­ecules.

Additional inter­molecular inter­actions towards the various solvate mol­ecules are observed. These are generally much weaker than the inter­actions described so far, with the possible exception of the water mol­ecules in the hemihydrate, which are partially hydrogen bonded to the main lattice. The partial occupancy of the water mol­ecules does, however, indicate that these hydrogen bonds are not essential in any way to sustain the overall structure, despite being individually quite strong. It appears that the water mol­ecules simply occupy the positions most suitable for them, but that they do not influence the overall structure much. This is further substanti­ated by the fact that the hemihydrate is isomorphous to the other solvates, with no indication that the structure is modulated much by the presence of the water mol­ecules. Their partial occupancy does, for example, not lead to disorder of the cations or anions, but the 1D chains are unfazed by the presence or absence of the water mol­ecules.

When the water mol­ecules are present, then they are located such that they are hydrogen bonded (Fig. 8 ▸). One of the mol­ecules, associated with O7 and about one quarter occupied [refined occupancy 0.276 (17)] is located in a general position and is hydrogen bonded to the maleate C=O group of O3 (which is also hydrogen bonded to the ammonium cation). The second solvate water mol­ecule, associated with O8, is located on a twofold axis, and is in hydrogen-bonding distance to the other water mol­ecule. It features a higher occupancy rate, 0.40 (4), but less than double that of the first water mol­ecule, indicating that it is hydrogen bonded to either O7 or to its symmetry-related counterpart by the twofold axis, but not to both at the same time. Its large displacement ellipsoid indicates possible unresolved disorder resulting from the varying environments and/or large thermal libration due to the absence of a second hydrogen-bonding partner and the presence of an unoccupied void space instead. No second acceptor site for the first water mol­ecule is present, which indicates that the overall structure is not well suited for inclusion of water in its lattice. A *PLATON* SQUEEZE analysis (van der Sluis & Spek, 1990 ▸; Spek, 2015 ▸) revealed 6.9% of additional void space not occupied by any solvate mol­ecules (even partially occupied). Crystals of the hemihydrate were grown from aceto­nitrile by evaporation with only trace amounts of water available from the solvent and the surrounding atmosphere, and crystals were exposed to atmosphere prior to analysis. Thus presence of additional aceto­nitrile solvate mol­ecules in the original crystals, which were subsequently lost, is likely. Attempts to grow single crystals from solvents with more available water have so far been unsuccessful, which indicates that the presence of larger amounts of water might result in formation of a different type of maleate salt. Further single crystal and powder XRD experiments are under way to investigate this possibility.

The less-than-ideal nature of the overall structure to host hydrogen-bonded guest solvate mol­ecules is supported by the ready formation of solvate structures with aprotic solvents, such as THF and acetone/hexane resulting in isomorphous structures with little or no modulation. Well-formed crystals could readily be grown from these solvents up to millimeters in size, showing how readily accessible this structural motif is.

THF mol­ecules in that solvate are only loosely bonded to anions and cations. Two sites occupied by THF mol­ecules were found. One located on a twofold axis and intrinsically 1:1 disordered. It is encapsulated between four different naphthyl groups and is weakly hydrogen bonded to all of them *via* C—H⋯O inter­actions originating from C9 and C12. The other mol­ecule is in a general position and exhibits no directional inter­actions with any neighboring entities at all, thus simply taking up the space provided by the lattice. The mol­ecule is disordered, in a refined ratio of 0.587 (16) to 0.413 (16), further supporting the absence of any steering inter­actions with its neighbors in space.

Acetone and hexane mol­ecules also do not strongly inter­act with the cations and anions in this solvate. Two distinct solvate-occupied sites are present in the lattice. One site is occupied by only acetone. This mol­ecule is located on and disordered around a twofold axis and is additionally disordered by a slight tilt of the mol­ecule. Occupancies refined to two × 0.230 (11) and two × 0.270 (11) for this site. The other solvate site is occupied by both acetone and hexane, with either one hexane mol­ecule located on another twofold axis, or two acetone mol­ecules being symmetry equivalent by this axis. The occupancy rates refined to 0.505 (9) and 0.495 (9) in slight favor of the acetone mol­ecules. The acetone mol­ecules of this site are weakly bound *via* a C—H⋯O inter­action to the meth­oxy methyl group and to one of the maleate C—H groups. No other directional inter­actions of either acetone or hexane with anions or cations are observed.

Inter­actions with solvate mol­ecules are more pronounced in the ethyl acetate solvate, but the exact nature of the inter­actions is obscured by substantial disorder, with up to fivefold disorder refined for one solvate cluster. Solvate disorder induces disorder of a cation phenyl group and a cation naphthyl group (see the *Refinement* section for a more detailed discussion of disorder). Some C—H⋯O inter­actions appear evident for the major disordered moieties though, which will be discussed below. The larger extent of the solvate inter­action with the main structure, when compared to the hemihydrate, THF and acetone/hexane solvates, is also supported by the fact that the ethyl acetate solvate is not exactly isomorphous with the other three solvates, but is modulated and crystallizes with lower symmetry than the other structures. *C*-centered and twofold symmetry are broken, resulting in a structure with a similar unit-cell size and shape, but with a primitive lattice and space group *P*2_1_. Exact translation and twofold symmetry for the ethyl acetate solvate is broken by ordering of the solvate mol­ecules and by a slight modulation of cations and anions (see *Refinement* section for more details).

The ethyl acetate mol­ecules are arranged into two clusters with light and severe disorder, refined as twofold and fivefold disorder, with partial overlap between the two clusters. Total occupancy for the severely disordered site refined to less than unity, just above 60% [0.641 (6)], inducing disorder for the surrounding naphthyl and phenyl groups. Additional unresolved disorder cannot be ruled out for this site. Despite the pseudo-translational symmetry, the two solvate sites are clearly distinct from each other, with little to no correlation effects between the two sites, and the differences between the two solvent sites appear to be the main reason for modulation and breaking of the *C*2 symmetry.

In the less disordered and fully occupied solvate site, the major moiety ethyl acetate [87.4 (3)% occupancy] is hydrogen bonded *via* its keto group to the meth­oxy methyl group, C32*A*. The major moiety mol­ecule of the other site, on the other hand, exhibits C–H⋯O bonds originating from methyl ammonium C5*A* and naphthyl C12*B*. The same inter­action is observed for one of the minor moieties at this site, with a combined occupancy rate of 35.4%, or more than half of the total site occupancy. No C—H⋯O inter­action originating from the meth­oxy methyl group C32*B* is present.

## Stability and desolvation, solvent-free salt   

The stronger inter­action of the ethyl acetate solvate mol­ecules with the framework mol­ecules, when compared to their THF and acetone/hexane analogues, also translates into the stabil­ity of the solvates. The THF and acetone/hexane analogues readily loose most of their solvate mol­ecules under ambient conditions, and crystals become opaque within a few hours. Crystals of the ethyl acetate solvate, under the same conditions, do not change in appearance. When taken out of solution and stored overnight, exposed to normal atmosphere, crystals of the ethyl acetate solvate are visually unchanged, and data collected from single crystals are unchanged from data collected from a crystal fresh out of mother liquor. Solvate mol­ecules are still clearly resolved, the disorder pattern is not changed noticeably, and occupancy rates are unchanged. The modulation of the main mol­ecule framework is preserved, unit-cell parameters are virtually unchanged (reduction by 0.3%), and mosaicity is essentially unchanged (0.73° and 0.77°, respectively; see supporting information, Fig. S1).

Crystals of the THF and acetone/hexane solvate behave differently. Crystals of either compound become milky within a few minutes of being taken out of mother liquor, and even the cores of large crystals (up to 1 mm) completely lose transparency within a few hours when stored in air outside the mother liquor. Crystals do, however, retain crystallinity, despite becoming opaque and white in appearance. Single-crystal data for such a crystal obtained from the acetone/hexane solvate did diffract well, with little to no loss of diffraction power compared to the solvated crystals or the ethyl acetate solvate (Fig. S1 in the supporting information), and there was only a small increase in mosaicity from 0.71° to 0.85° after storing in air for 14 h.

Hot stage microscopy showed that if the crystals are crushed, solvent loss is rapid for both the acetone/hexane as well as the ethyl acetate samples. When single crystals were crushed on a microscope slide and heated at 10.0°C min^−1^ to 110.0°C, then at 5.0°C min^−1^ to 120.0°C and then finally at 2.0°C min^−1^ to 140.0°C, no loss of solvate mol­ecules was observable for either the acetone/hexane nor the ethyl acetate crystals for either a dry sample or immersed in mineral oil. Onset of melting was observed between 122.1 and 124.5°C, and melting was complete at 128.3 to 133.7°C, with no noticeable difference between the acetone/hexane and the ethyl acetate sample (Table 9 ▸, selected figures shown in the supporting information). The comparable melting temperatures of these materials support the finding from XRD that the crystal structures of the maleate solvates are isomorphically related and that the presence of solvate is not required to maintain these structures. This indicates that for smaller particles, solvate mol­ecules are rapidly lost for both solvates, possibly before start of the hot stage microscopy experiment, while larger crystals of the ethyl acetate solvate (> 200 µm^3^ such as used for single-crystal diffraction) do not desolvate readily and retain most of their solvate mol­ecules.

For the acetone/hexane sample stored in atmosphere overnight, when analyzed by SC-XRD, a noticeable change of the unit-cell dimensions was observed, accompanied by a decrease in volume by *ca* 3.5% [from 3733.9 (3) to 3603.0 (5) Å^3^]. An overlay of the structure before and after desolvation is shown in the supporting information (Fig. S2). Changes of unit-cell parameters and a slight shifting of functional groups are perceptible, but the overall magnitude of those changes is small.

**Figure 9:**
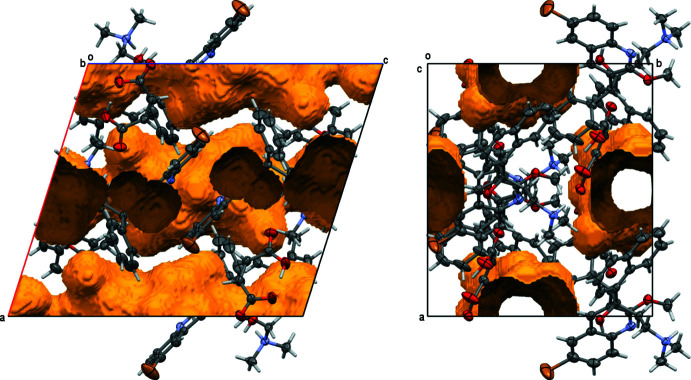
Residual void space in the acetone/hexane structure after artificial removal of solvent mol­ecules. The solvent-accessible volume would be 781 Å^3^ [20.9% of the unit-cell volume; probe radius 1.2 Å; numerical values from *PLATON* SQUEEZE calculation (van der Sluis & Spek, 1990 ▸; Spek, 2015 ▸)]. Probability ellipsoids are at the 50% level.

**Figure 10:**
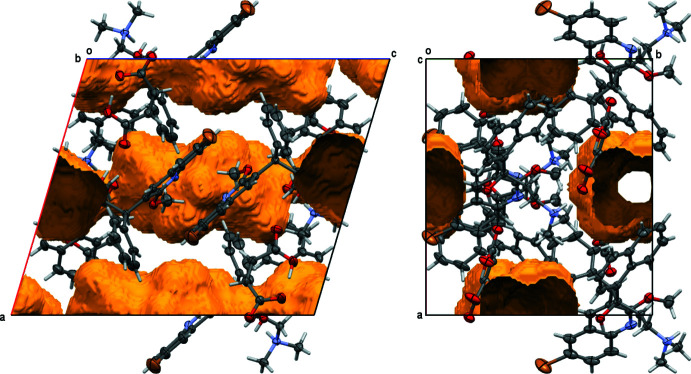
Residual void space in the desolvated structure. The solvent-accessible volume is 607 Å^3^ [16.8% of the unit cell volume; probe radius 1.2 Å; numerical values from *PLATON* SQUEEZE calculation (van der Sluis & Spek, 1990 ▸; Spek, 2015 ▸)]. Probability ellipsoids are at the 50% level.

The decrease of the unit-cell volume is, however, substanti­ally less than the 20.9% of the volume taken up by solvate mol­ecules in the acetone/hexane solvate, Fig. 9 ▸. Indeed, a *PLATON* SQUEEZE analysis (van der Sluis & Spek, 1990 ▸; Spek, 2015 ▸) reveals a residual void space of 16.8% of the unit-cell volume, Fig. 10 ▸. This indicates that either a substantial fraction of the solvate mol­ecules is retained, or that the hydrogen-bonded framework is stable enough to withstand collapse, even without any solvate mol­ecules in the void space between the bedaquilinium maleate framework. A future in-depth analysis of several bedaquilinium salts, including the various solvates of the maleate system, will focus on their thermal stability and physical properties, and will include thermal gravimetric analysis, porosity measurements of the desolvated salts and surface-area measurements. The single-crystal structure of the acetone/hexane crystals stored under ambient conditions does, however, already provide some first insights. Analysis of the data revealed a well-defined bedaquilinium maleate framework, with barely any increased libration, but a completely featureless electron-density difference map for the areas previously taken up by the acetone/hexane mol­ecules. The largest difference-electron peaks inside the void area are less than 0.5 e Å^3^. A solvate SQUEEZE analysis performed using the program *PLATON* revealed some residual electron density, but substanti­ally less than what would be expected for full occupancy. The SQUEEZE procedure corrected for 66 electrons within the solvent-accessible voids, equivalent to 1.14 mol­ecules of acetone per unit cell, or 0.28 acetone per cation–anion pair. Prior to desolvation, one mol­ecule of acetone and half a mol­ecule of hexane were present per cation–anion pair, equivalent to 202 electrons per unit cell. Thus, there seems to be some retention of solvate mol­ecules within the voids (*ca* one third based on the SQUEEZE data), but those solvate mol­ecules appear to be completely disordered and equally distributed within the solvate-accessible area. The bedaquil­inium maleate framework is not affected by the residual solvate. No disorder is observed for either cation or anion, nor any increased libration, indicating that any residual solvate has negligible inter­action with the framework, and that desolvation is homogeneous throughout the whole crystal. Thermal gravimetric analysis and surface measurements, to be reported in an upcoming publication, will provide more insight as to how much or if any residual solvates are indeed present in the void area.

## Database survey   

Only four structures of bedaquiline or its salts have been previously reported in the literature (Cambridge Structural Database; Groom *et al.*, 2016 ▸), *viz*. free base bedaquiline (Petit *et al.*, 2007 ▸), the fumarate salt (Okezue *et al.*, 2020 ▸), and two isomorphous solvates of the benzoate salt (Okezue *et al.*, 2020 ▸). The structures of the salts are dominated by a multitude of N—H⋯O and O—H⋯O hydrogen-bonding inter­actions that connect the cations and anions into strongly hydrogen-bonded motifs, while in free base bedaquiline the packing is dominated by weaker and less directional inter­actions such as Br⋯Br inter­actions and π-stacking (Petit *et al.*, 2016 ▸). In all structures, the ethane backbone and the malleable ethyl­amine fragment of the bedaquiline core give the cations a high degree of flexibility, and mol­ecular conformations not only vary widely between the bedaquiline and bedaquilinium structures, but even between independent mol­ecules within the same structure (both the free base and the fumarate are *Z*′ = 2 structures). The fumarate salt was solvent free. The benzoate salt formed a hydrate with one strongly bound solvate water mol­ecule, and a second solvate site occupied either partially by water (occupancy 17%) or by disordered aceto­nitrile. The aceto­nitrile solvate was prone to desolvation and converted quickly into a simple monohydrate once taken out of solution. In both of the salts, the anions and cations are bridged *via* hydrogen atoms into 2D ribbons in which the bedaquilinum cations wrap around a single strand of anions (fumarate) or around anions and water mol­ecules (benzoate). This differentiates the fumarate and benzoate salts from the maleates, which exhibit simpler 1D chains of anions and cations.

## Methods and procedures   

Maleic acid was purchased from BTC, THF from VWR chemicals, acetone from VWR chemicals, ethyl acetate from Macron, and aceto­nitrile from VWR Chemicals. Bedaquil­inium fumarate was obtained from Johnson & Johnson. All chemicals were used as received without further purification. Free base bedaquiline was prepared by extracting a CH_2_Cl_2_ solution of the fumarate three times with saturated NaHCO_3_ solution (Rombouts *et al.*, 2016 ▸).

## Hot stage optical microscopy   

Analyses were completed using an Olympus Series BX51TRF microscope (Olympus America Inc., Melville, NY) equipped with 12V/100W illumination, an achromat 0.9 NA polarized light condenser, a 20X, 0.40 Numerical Aperture, LM PLAN FL N objective, an inter­mediate tube with variable position analyzer and first-order red compensator, a trinocular viewing head with a Lumenera Series Infinity X (Teledyne Lumenera, Ottawa, Ontario, Canada) digital camera using *Infinity* software version 6.5.6 and *Infinity Analyze* software version 7.0.2.930 (build date 01-Feb-2020). Heating was conducted with a Linkam LTS420 hot stage with a T95 LinkPad system controller. A single crystal of either the acetone/hexa­nes or ethyl acetate (200 µm^3^) was removed from the solvent and crushed on a clean microscope slide under a No. 1 1/2 cover glass. A small portion of each sample was transferred to three individual clean microscope slides under a No. 1 1/2 cover glass for analysis. A preset heating program ramp was used during each individual analysis using the hot stage system controller programmed with a ramp of 10.0°C min^−1^ to 110.0°C, at 5.0°C min^−1^ to 120.0°C, and at 2.0°C min^−1^ to 140.0°C. The system calibration was verified with melting-point standards prior to analyses. Samples were analyzed in triplicate, twice as dry mounts and once in mineral oil, USP (CAS: 8042-47-5), which was allowed to cover the sample by capillarity. Sample and thermomicroscopy information is given in Table 9 ▸, selected images are given in the supporting information.

## Synthesis and crystallization   

**Hemihydrate, C_32_H_32_BrN_2_O_2_·C_4_H_3_O_4_·0.5H_2_O:** Bedaquiline free base (400.3 mg) was weighed into a 20 mL glass scintillation vial and dissolved in 3 mL of THF. Maleic acid (85.6 mg) was added and the contents mixed. The solution was allowed to evaporate slowly at ambient conditions. White crystals that appeared dry were evident within two days in the vial. Approximately 14 mg of this dried material was weighed into a 2 dram glass vial, re-dissolved in 800 µL ACN by vortexing/sonicating until just dissolved, and wrapped in aluminum foil. An 18 gauge needle was placed into the top of the conical-shaped foil to allow for slow evaporation at ambient conditions until solids were evident and the sample appeared dry.

**THF solvate, C_32_H_32_BrN_2_O_2_·C_4_H_3_O_4_·1.5C_4_H_8_O:** Bedaquiline free base (400.3 mg) was weighed into a 20 mL glass scintillation vial and dissolved in 3 mL THF. Maleic acid (85.6 mg) was added and the contents mixed. The solution was allowed to evaporate slowly at ambient conditions. White crystals that appeared dry were evident within two days in the vial. Approximately 16 mg of this dried material was weighed into a 2 dram glass vial, re-dissolved in 1200 µL THF, and wrapped in aluminum foil. An 18 gauge needle was inserted into the top of the aluminum foil to allow for slow evaporation at ambient conditions until solids were evident and the sample appeared dry.

**Acetone/hexane solvate, C_32_H_32_BrN_2_O_2_·C_4_H_3_O_4_·C_2_H_6_O_2_·0.25C_6_H_14_:** Bedaquiline maleate (23.1 mg) was weighed into a glass vial and dissolved in acetone (3 mL). A layer of *n*-hexa­nes was gently streamed onto the top of the solution. The vial contents were capped and placed under a hood at ambient conditions. After two days, clear crystals were evident in the vial.

**Desolvated structure, C_32_H_32_BrN_2_O_2_·C_4_H_3_O_4_:** Crystals of the solvent-free compound were obtained from the monoacetone quadrant-hexane solvate by drying in air on a microscope slide. Crystals become milky overnight when taken out of mother liquor solution and left to dry in air, but retain crystallinity. Data collection revealed a solvent-free structure.

**Ethyl acetate solvate, C_32_H_32_BrN_2_O_2_·C_4_H_3_O_4_·0.821C_4_H_8_O_2_:** Bedaquiline maleate (22.3 mg) was weighed into glass vial and dissolved in ethyl acetate (4 mL). A layer of *n*-hexa­nes was gently streamed onto the top of the solution. The vial contents were capped and allowed to equilibrate at ambient conditions. After two days, clear crystals were evident in the vial.

## Refinement   

Crystal data, data collection and structure refinement details are summarized in Table 10 ▸.

The structures of the four solvates and of the solvent-free salt are closely related. The hemihydrate, THF solvate, the acetone/hexane solvate and the solvate-free salt derived from the acetone/hexane solvate are isomorphous in space group *C*2. In the ethyl acetate solvate, *C*-centered and twofold symmetry is broken and the salt crystallizes in *P*2_1_, but the structure is closely related to the other *C*-centered structures.

The four isomorphous structures were refined using a common model for the non-solvate part of the structures, with the THF solvate, the acetone/hexane solvate and the desolvated structure solved by isomorphous replacement. The ethyl acetate solvate was solved independently, by dual Patterson/direct methods, but the atom-naming scheme from the other structures was adopted and augmented by suffixes *A* and *B* to distinguish between the two cation–anion pairs related by pseudo-translation.

**Hydrogen-atom treatment**: C—H bond distances were constrained to 0.95 Å for aromatic and alkene C—H moieties, and to 1.00, 0.99 and 0.98 Å for aliphatic C—H, CH_2_ and CH_3_ moieties, respectively. N—H bond distances were constrained to 1.00 Å for pyramidal (*sp* ^3^ hybridized) ammonium *R*_3_H^+^ groups. O—H distances of alcohols were constrained to 0.84 Å. Methyl CH_3_ and hydroxyl H atoms were allowed to rotate but not to tip to best fit the experimental electron density. The positions of hydro­maleate acidic hydrogen atoms were freely refined. Water H-atom positions in the hemihydrate were refined and O—H and H⋯H distances were restrained to 0.84 (2) and 1.36 (2) Å, respectively. H-atom positions were further restrained based on hydrogen-bonding considerations. A damping factor was applied during refinement. In the final refinement cycles, the damping factor was removed and the water H atoms were constrained to ride on their carrier oxygen atoms. *U*_iso_(H) values were set to a multiple of *U*_eq_(C/O/N) with 1.5 for CH_3_ and OH, and 1.2 for C—H, CH_2_ and N—H units, respectively.

**Disorder and solvate refinement, handling of void space:**

In the hemihydrate, two partially occupied water mol­ecules are situated in the asymmetric part of the unit cell. One is in a general position, the other located on a twofold axis. They are hydrogen bonded to each other, and the one in the general position is also hydrogen bonded to atom O3 of the hydro­maleate anion. Water H-atom positions were refined as described above. Occupancy rates refined to 0.276 (17) for O7 (in the general position) and 0.40 (4) for O8 (on the twofold axis).

Additional solvent-accessible space is present in the crystal lattice (two × 123 Å^3^ or 6.9% of the unit-cell volume). No electron density was found inside the void space [a *PLATON* SQUEEZE analysis (van der Sluis & Spek, 1990 ▸; Spek, 2015 ▸) found eight electrons in the combined void space], and the content of the void space was ignored.

In the THF solvate, two THF mol­ecules were refined as disordered, one in a 1:1 ratio around a twofold rotation axis, the other in a general position. The three disordered moieties were restrained to have similar geometries. *U^ij^* components of ADPs for disordered atoms closer to each other than 2.0 Å were restrained to be similar. Subject to these conditions, the occupancy ratio for the mol­ecule in the general position refined to 0.587 (16) to 0.413 (16).

In the acetone/hexane solvate, two symmetry-equivalent acetone mol­ecules are disordered with a hexane mol­ecule located on a twofold axis. Another acetone mol­ecule is located on and disordered around a twofold axis and additionally disordered by a slight tilt of the mol­ecule. All acetone moieties were restrained to have similar geometries and to be close to planar. The two C—C bond distances within the acetone were restrained to be similar to each other. C—C bond distances of the hexane mol­ecule were restrained to target values [1.55 (1) Å] and hexane C—C—C angles were restrained to be similar. *U^ij^* components of ADPs for disordered atoms closer to each other than 2.0 Å were restrained to be similar. Subject to these conditions, the occupancy rates refined to 0.505 (9) and 0.495 (9) for the acetone/hexane disorder, and two × 0.230 (11) and two × 0.270 (11) for the disordered acetone.

The structure of the ethyl acetate solvate exhibits pseudo *C*-centered symmetry emulating space group *C*2 as observed for the hemihydrate, THF and acetone/hexane solvates and the solvent-free salt derived from the acetone/hexane solvate. Exact translation and twofold symmetry for the ethyl acetate solvate is broken by the solvate mol­ecules and by a slight modulation of cations and anions. The mean intensity for reflections that should be systematically absent in *C*2 was 1.8, *vs* 6.9 for all reflections (2.2 *vs* 2.9 for mean intensity/σ).

Ethyl acetate mol­ecules are arranged into two clusters with light and severe disorder. Solvate disorder induces disorder of a cation phenyl and a cation naphthyl group. The site associated with the ethyl acetate mol­ecule of O1/O2 was refined as twofold disordered and as fully occupied. The site associated with the ethyl acetate mol­ecule of O3/O4 was refined as fivefold disordered and only partially occupied. One of the moieties of O3/O4 (suffix *F*) extends away from the main cluster. It induces the disorder of the O1/O2 ethyl acetate, and for the naphthyl group of cation *A*. A common occupancy ratio was used for these three entities. Disorder of the phenyl group of cation *B* is correlated with multiple disordered moieties of the severely disordered ethyl acetate and was refined independently.

All ethyl acetate moieties were restrained to have similar geometries. The acetate sections were restrained to be close to planar. The ethyl C—C bond distances were restrained to a target value [1.55 (2) Å]. Disordered phenyl and naphthyl groups were restrained to have similar geometries as their not disordered counterparts in the other cation. *U^ij^* components of ADPs for disordered atoms closer to each other than 2.0 Å were restrained to be similar. Subject to these conditions, the occupancy rates refined to 0.874 (3) to 0.126 (3) for the twofold-disordered ethyl acetate of O1/O2 (shared with the naphthyl disorder of cation *A*). The occupancy rates for the partially occupied site refined to 0.171 (7), 0.183 (7), 0.126 (3) (the same as minor moiety of O1/O2 ethyl acetate), 0.074 (6) and 0.087 (6), for a total occupancy of 0.641. The occupancy ratio of the phenyl disorder of cation *B* refined to 0.573 (17) to 0.427 (17).

Crystals of the solvate-free salt were obtained from the acetone/hexane solvate by drying on a glass slide in air overnight. In the solvated structure, acetone and hexane mol­ecules are located in infinite channels and slowly vacate the crystal lattice. Crystals become milky overnight when taken out of mother liquor solution and left to dry in air, but retain crystallinity.

No substantial electron density was found in the previously solvate-occupied channels (largest void peaks are less than 0.5 electrons per cubic Ångstrom), and the residual electron-density peaks are not arranged in an inter­pretable pattern. The structure was refined both with and without correction of residual electron density, with only marginally different results. In the second approach, the structure factors were augmented *via* reverse Fourier transform methods using the SQUEEZE routine (van der Sluis & Spek, 1990 ▸; Spek, 2015 ▸) as implemented in the program *PLATON*. The resultant FAB file containing the structure-factor contribution from the electron content of the void space was used together with the original hkl file in the further refinement. (The FAB file with details of the SQUEEZE results is appended to the CIF). The SQUEEZE procedure corrected for 66 electrons within the solvent-accessible voids, equivalent to 1.14 mol­ecules of acetone per unit cell, or 0.28 acetone per cation–anion pair. Prior to desolvation, one mol­ecule of acetone and a quarter mol­ecule of hexane were determined per cation–anion pair [the *F*(000) values of the solvated and unsolvated structures differ by 178 electrons].

## Supplementary Material

Crystal structure: contains datablock(s) hemihydrate, THF, ethyl_acetate, acetone_hexane, desolvated, desolvated_sq, global. DOI: 10.1107/S2056989021002991/tx2038sup1.cif

Example diffraction patterns; unit-cell overlay for acetone/hexane solvate and desolvated structure; selected hot stage microscopy images. DOI: 10.1107/S2056989021002991/tx2038sup8.pdf

Structure factors: contains datablock(s) hemihydrate. DOI: 10.1107/S2056989021002991/tx2038hemihydratesup2.hkl

Structure factors: contains datablock(s) THF. DOI: 10.1107/S2056989021002991/tx2038THFsup3.hkl

Structure factors: contains datablock(s) ethyl_acetate. DOI: 10.1107/S2056989021002991/tx2038ethyl_acetatesup4.hkl

Structure factors: contains datablock(s) acetone_hexane. DOI: 10.1107/S2056989021002991/tx2038acetone_hexanesup5.hkl

Structure factors: contains datablock(s) desolvated. DOI: 10.1107/S2056989021002991/tx2038desolvatedsup6.hkl

Structure factors: contains datablock(s) desolvated_sq. DOI: 10.1107/S2056989021002991/tx2038desolvated_sqsup7.hkl

CCDC references: 2072581, 2072582, 2072583, 2072584, 2072585, 2072586

Additional supporting information:  crystallographic information; 3D view; checkCIF report

## Figures and Tables

**Figure 2:**
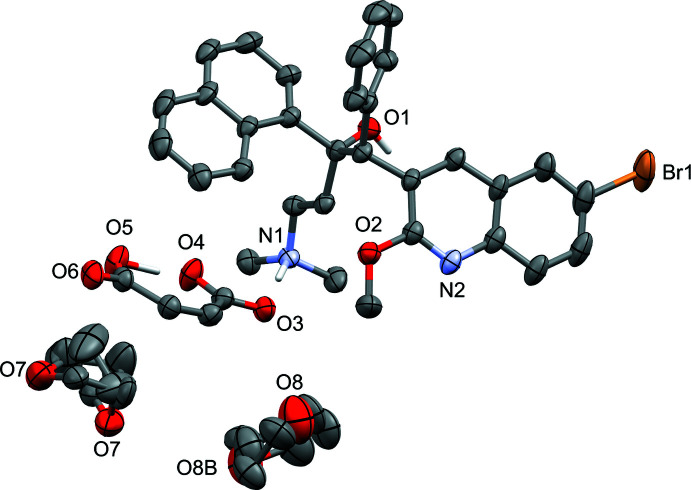
Probability ellipsoid plot (50% probability) of the THF solvate (see Fig. 1 ▸ for cation and anion carbon-atom labels). C-bound H atoms and labels for C and H atoms are omitted for clarity. THF mol­ecules are disordered around a twofold axis (O7) or in a general position (O8).

**Figure 3:**
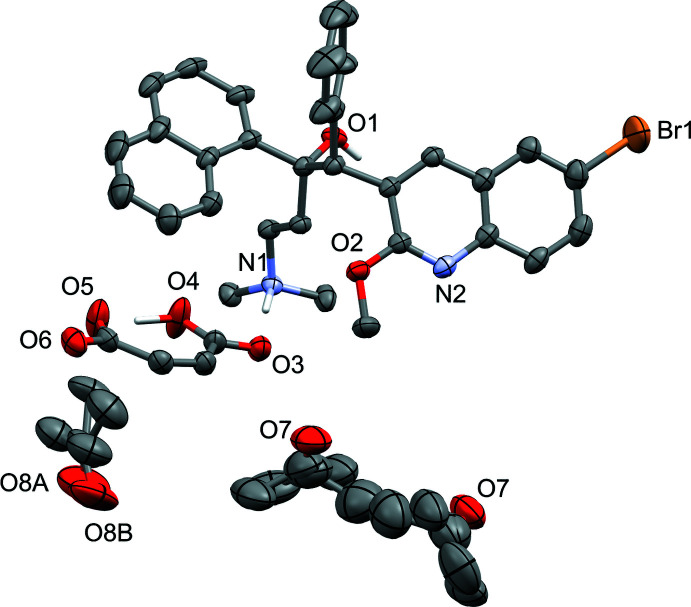
Probability ellipsoid plot (50% probability) of the acetone/hexane solvate (see Fig. 1 ▸ for cation and anion carbon-atom labels). C-bound H atoms and labels for C and H atoms are omitted for clarity. Acetone mol­ecules are disordered: fourfold around a twofold axis plus general disorder (O8, disorder by the twofold axis not shown for clarity) or with a hexane mol­ecule (O7, the hexane mol­ecule is located on a twofold axis).

**Figure 4:**
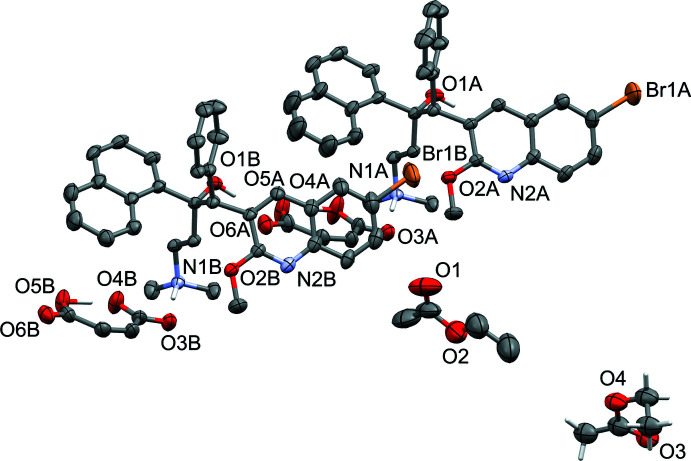
Probability ellipsoid plot (50% probability) of the ethyl acetate solvate, showing both ion pairs (suffixes *A* and *B*) related by pseudo-translation (see Fig. 1 ▸ for cation and anion carbon-atom labels). C-bound H atoms, labels for C and H atoms and disorder of ethyl acetate mol­ecules are omitted for clarity.

**Figure 5:**
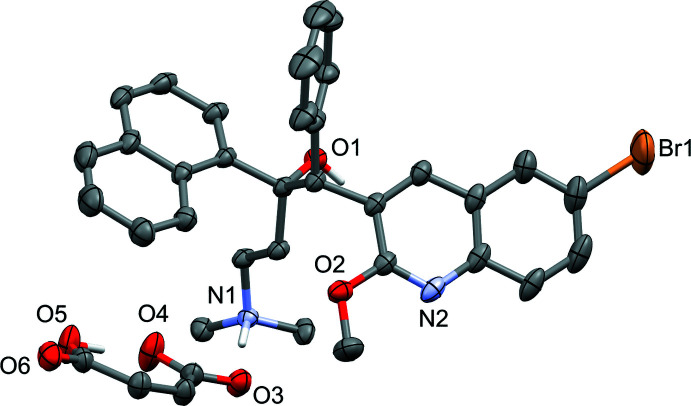
Probability ellipsoid plot (50% probability) of the desolvated structure (see Fig. 1 ▸ for cation and anion carbon-atom labels). C-bound H atoms and labels for C and H atoms are omitted for clarity.

**Table 1:** Selected torsion angles for bedaquilinium maleate structures

	hemihydrate	THF	ethyl acetate	acetone/hexa­ne	desolvated
τ plane 1 *vs* plane 2	71.31 (7)	64.3 (1)	67.78 (7), 63.0 (2)	70.00 (6)	70.34 (5)
τ plane 2 *vs* plane 3	65.35 (9)	63.7 (1)	65.8 (1), 64.0 (2)	66.60 (7)	64.89 (6)
τ plane 1 *vs* plane 3	32.62 (9)	26.4 (1)	31.7 (1), 31.50 (9)	29.47 (7)	32.06 (6)
τ C1—C2—C3—C4	175.6 (3)	174.1 (4)	176.4 (3), 175.9 (3)	176.2 (2)	175.85 (18)
τ C2—C3—C4—N1	169.4 (3)	174.3 (4)	173.9 (3), 174.6 (3)	172.86 (19)	169.78 (18)
τ C17—C1—C2—C3	−170.8 (3)	−169.8 (4)	−172.0 (3), −164.4 (7)	−172.3 (2)	−171.37 (19)

**Table 2:** Hydrogen-bond geometry (Å, °) for the hemihydrate[Chem scheme1]

*D*—H⋯*A*	*D*—H	H⋯*A*	*D*⋯*A*	*D*—H⋯*A*
O1—H1*O*⋯O6^i^	0.84	1.98	2.822 (4)	175
O5—H5⋯O4	1.13 (6)	1.29 (6)	2.417 (4)	169 (5)
N1—H1⋯O3	1.00	1.71	2.707 (4)	172
N1—H1⋯O4	1.00	2.49	3.174 (4)	125
C3—H3*A*⋯O6^i^	0.99	2.57	3.308 (4)	131
C6—H6*B*⋯O4^ii^	0.98	2.61	3.257 (5)	124
O7—H7*A*⋯O3	0.84	2.31	3.139 (16)	172
O8—H8*A*⋯O7	0.94	2.13	3.02 (2)	158

Symmetry codes: (i) x-{\script{1\over 2}}, y-{\script{1\over 2}}, z; (ii) -x+1, y, -z.

**Table 3:** Hydrogen-bond geometry (Å, °) for the THF solvate[Chem scheme1]

*D*—H⋯*A*	*D*—H	H⋯*A*	*D*⋯*A*	*D*—H⋯*A*
O1—H1*O*⋯O6^i^	0.84	1.99	2.824 (5)	176
O4—H5⋯O5	1.15 (8)	1.28 (8)	2.422 (5)	171 (7)
N1—H1⋯O3	1.00	1.70	2.699 (5)	175
N1—H1⋯O4	1.00	2.63	3.310 (6)	125
C3—H3*A*⋯O6^i^	0.99	2.53	3.258 (6)	130
C3—H3*B*⋯O3	0.99	2.65	3.314 (6)	125
C6—H6*B*⋯O4^ii^	0.98	2.55	3.173 (7)	122
C26—H26⋯O8*B* ^i^	0.95	2.57	3.44 (3)	152
C42—H42*B*⋯O3^iii^	0.99	2.67	3.478 (17)	138

Symmetry codes: (i) x-{\script{1\over 2}}, y-{\script{1\over 2}}, z; (ii) -x+1, y, -z; (iii) x+{\script{1\over 2}}, y-{\script{1\over 2}}, z.

**Table 4:** Hydrogen-bond geometry (Å, °) for the ethyl acetate solvate[Chem scheme1]

*D*—H⋯*A*	*D*—H	H⋯*A*	*D*⋯*A*	*D*—H⋯*A*
O1*A*—H1*AB*⋯O6*B* ^i^	0.84 (6)	1.98 (6)	2.791 (4)	160 (6)
O5*A*—H5*A*⋯O4*A*	1.11 (10)	1.33 (10)	2.426 (5)	171 (9)
N1*A*—H1*A*⋯O3*A*	1.00	1.71	2.707 (4)	175
N1*A*—H1*A*⋯O4*A*	1.00	2.54	3.211 (4)	125
O1*B*—H1*BB*⋯O6*A*	0.92 (6)	1.91 (6)	2.812 (4)	168 (6)
O5*B*—H5*B*⋯O4*B*	1.17 (8)	1.29 (8)	2.429 (4)	164 (6)
N1*B*—H1*B*⋯O3*B*	1.00	1.70	2.701 (4)	174
N1*B*—H1*B*⋯O4*B*	1.00	2.52	3.204 (4)	125
C3*A*—H3*AA*⋯O6*B* ^i^	0.99	2.56	3.251 (4)	127
C5*A*—H5*AC*⋯O3^ii^	0.98	2.60	3.391 (15)	138
C6*A*—H6*AA*⋯O4*B* ^iii^	0.98	2.59	3.218 (5)	122
C32*A*—H32*A*⋯O1^iv^	0.98	2.59	3.220 (7)	122
C1*B*—H1*BA*⋯O2*B*	1.00	2.26	2.780 (4)	111
C3*B*—H3*BA*⋯O6*A*	0.99	2.58	3.284 (4)	128
C6*B*—H6*BA*⋯O4*A* ^v^	0.98	2.58	3.190 (5)	121
C2*E*—H2*EB*⋯O3*A* ^vi^	0.98	2.34	2.96 (2)	120
C4*E*—H4*EB*⋯O1*E*	0.98	2.37	2.91 (5)	114
C7—H7*A*⋯O4*A* ^iii^	0.99	2.29	3.068 (15)	134
C8*E*—H8*EC*⋯O5*A* ^iii^	0.98	2.65	3.57 (3)	157
C8*G*—H8*GB*⋯O1*A* ^v^	0.98	2.05	2.90 (5)	145

Symmetry codes: (i) x-1, y-1, z; (ii) x-1, y, z; (iii) -x+1, y-{\script{1\over 2}}, -z; (iv) -x+1, y+{\script{1\over 2}}, -z+1; (v) -x+1, y+{\script{1\over 2}}, -z; (vi) -x+1, y-{\script{1\over 2}}, -z+1.

**Table 5:** Hydrogen-bond geometry (Å, °) for the acetone/hexane solvate[Chem scheme1]

*D*—H⋯*A*	*D*—H	H⋯*A*	*D*⋯*A*	*D*—H⋯*A*
O1—H1*O*⋯O6^i^	0.84	1.98	2.816 (3)	175
O5—H5⋯O4	1.18 (5)	1.24 (5)	2.422 (3)	175 (5)
N1—H1⋯O3	1.00	1.71	2.709 (3)	174
N1—H1⋯O4	1.00	2.54	3.212 (3)	125
C32—H32*B*⋯O7	0.98	2.60	3.337 (8)	132
C34—H34⋯O7	0.95	2.66	3.600 (9)	171

Symmetry code: (i) x-{\script{1\over 2}}, y-{\script{1\over 2}}, z.

**Table 6:** Hydrogen-bond geometry (Å, °) for the desolvated structure[Chem scheme1]

*D*—H⋯*A*	*D*—H	H⋯*A*	*D*⋯*A*	*D*—H⋯*A*
O1—H1*O*⋯O6^i^	0.84	1.98	2.818 (3)	180
O5—H5⋯O4	0.99 (5)	1.43 (5)	2.419 (3)	172 (5)
N1—H1⋯O3	1.00	1.71	2.704 (3)	172
N1—H1⋯O4	1.00	2.51	3.188 (3)	125

Symmetry code: (i) x-{\script{1\over 2}}, y-{\script{1\over 2}}, z.

**Table 7:** Hydrogen-bond geometry (Å, °) for the desolvated structure (SQUEEZE applied)

*D*—H⋯*A*	*D*—H	H⋯*A*	*D*⋯*A*	*D*—H⋯*A*
O1—H1*O*⋯O6^i^	0.84	1.98	2.820 (2)	180
O5—H5⋯O4	1.01 (4)	1.42 (4)	2.420 (3)	169 (4)
N1—H1⋯O3	1.00	1.71	2.705 (2)	172
N1—H1⋯O4	1.00	2.51	3.186 (2)	125

Symmetry code: (i) x-{\script{1\over 2}}, y-{\script{1\over 2}}, z.

**Table 8:** Bond distances and angles involving the intra­molecular O⋯H⋯O hydrogen bond of the hydro­maleate anions (Å, °)

	hemihydrate	THF	ethyl acetate	acetone/hexa­ne	desolvated
O5—H5	1.13 (6)	1.28 (8)	1.11 (10) *A* 1.17 (8) *B*	1.18 (5)	0.99 (5)
O4—H5	1.29 (6)	1.15 (8)	1.33 (10) *A* 1.29 (8) *B*	1.24 (5)	1.43 (5)
O5⋯H5⋯O6	169 (5)	171 (7)	171 (9) *A* 164 (6) *B*	175 (5)	172 (5)
C33—O4	1.275 (5)	1.275 (7)	1.246 (3) *A* 1.266 (5) *B*	1.271 (3)	1.276 (3)
C36—O5	1.290 (5)	1.301 (6)	1.292 (5) *A* 1.293 (5) *B*	1.283 (3)	1.283 (3)
C33—O3	1.237 (5)	1.234 (6)	1.245 (5) *A* 1.241 (5) *B*	1.230 (3)	1.238 (3)
C36—O6	1.231 (5)	1.217 (6)	1.216 (5) *A* 1.216 (5) *B*	1.222 (3)	1.233 (3)

**Table 9:** Hot stage optical microscopy data for the acetone/hexane and the ethyl acetate crystals

	Run 1	Run 2	Run 3 (in mineral oil)
**acetone/hexane solvate**, onset and end of melting	124.5°C, 133.7°C	122.1°C, 133.7°C	119.9°C, 128.8°*C*
**ethyl acetate solvate**, onset and end of melting	122.1°C, 133.6°C	122.8°C, 133.6°C	118.4°C, 128.3°C

**Table:** 

	hemihydrate	THF solvate	ethyl acetate solvate	acetone/hexane solvate
Crystal data				
Chemical formula	C_32_H_32_BrN_2_O_2_^+^·C_4_H_3_O_4_^−^·0.476H_2_O	2C_32_H_32_BrN_2_O_2_^+^·2C_4_H_3_O_4_^−^·3C_4_H_8_O	C_32_H_32_BrN_2_O_2_^+^·C_4_H_3_O_4_^−^·0.821C_4_H_8_O_2_	2C_32_H_32_BrN_2_O_2_^+^·2C_4_H_3_O_4_^−^·0.495C_6_H_14_·2.01C_3_H_6_O
*M*_r_	680.17	1559.45	743.88	1502.51
Crystal system, space group	Monoclinic, *C*2	Monoclinic, *C*2	Monoclinic, *P*2_1_	Monoclinic, *C*2
Temperature (K)	150	150	150	150
*a*, *b*, *c* (Å)	15.7469 (5), 13.2627 (4), 17.8602 (6)	16.4119 (6), 13.5643 (6), 17.8475 (8)	16.1525 (10), 13.5353 (9), 17.8572 (11)	16.0678 (9), 13.6440 (8), 17.8720 (8)
β (°)	106.3762 (13)	107.318 (3)	107.359 (2)	107.6347 (18)
*V* (Å^3^)	3578.7 (2)	3793.0 (3)	3726.3 (4)	3733.9 (3)
*Z*	4	2	4	2
Radiation type	Cu *K*α	Cu *K*α	Mo *K*α	Mo *K*α
μ (mm^−1^)	1.94	1.92	1.16	1.15
Crystal size (mm)	0.21 × 0.17 × 0.13	0.33 × 0.19 × 0.16	0.45 × 0.43 × 0.37	0.48 × 0.35 × 0.21

Data collection				
Diffractometer	Bruker AXS D8 Quest with PhotonIII C14 CPAD	Bruker AXS D8 Quest with PhotonIII C14 CPAD	Bruker AXS D8 Quest with PhotonII CPAD	Bruker AXS D8 Quest with PhotonII CPAD
Absorption correction	Multi-scan (*SADABS*; Krause *et al.*, 2015 ▸)	Multi-scan (*SADABS*; Krause *et al.*, 2015 ▸)	Multi-scan (*SADABS*; Krause *et al.*, 2015 ▸)	Multi-scan (*SADABS*; Krause *et al.*, 2015 ▸)
*T*_min_, *T*_max_	0.646, 0.754	0.254, 0.391	0.670, 0.742	0.439, 0.498
No. of measured, independent and observed [*I* > 2σ(*I*)] reflections	15060, 7003, 6474	18660, 7473, 6615	153291, 28338, 15758	73087, 14074, 9700
*R*_int_	0.040	0.049	0.082	0.059
(sin θ/λ)_max_ (Å^−1^)	0.638	0.639	0.770	0.769

Refinement				
*R*[*F* ^2^ > 2σ(*F* ^2^)], *wR*(*F* ^2^), *S*	0.041, 0.107, 1.08	0.054, 0.127, 1.05	0.062, 0.200, 1.03	0.047, 0.126, 1.03
No. of reflections	7003	7473	28338	14074
No. of parameters	430	551	1359	558
No. of restraints	8	223	1870	407
H-atom treatment	H atoms treated by a mixture of independent and constrained refinement	H atoms treated by a mixture of independent and constrained refinement	H atoms treated by a mixture of independent and constrained refinement	H atoms treated by a mixture of independent and constrained refinement
Δρ_max_, Δρ_min_ (e Å^−3^)	0.35, −0.46	0.46, −0.52	0.88, −1.03	0.61, −0.62
Absolute structure	Flack *x* determined using 2633 quotients [(*I* ^+^)−(*I* ^−^)]/[(*I* ^+^)+(*I* ^−^)] (Parsons *et al.*, 2013 ▸)	Refined as an inversion twin	Flack *x* determined using 5601 quotients [(*I* ^+^)−(*I* ^−^)]/[(*I* ^+^)+(*I* ^−^)] (Parsons *et al.*, 2013 ▸).	Flack *x* determined using 3753 quotients [(*I* ^+^)−(*I* ^−^)]/[(*I* ^+^)+(*I* ^−^)] (Parsons *et al.*, 2013 ▸)
Absolute structure parameter	0.019 (8)	0.03 (3)	0.020 (4)	0.006 (3)

**Table:** 

	desolvated structure	desolvated structure (SQUEEZE applied)
Crystal data		
Chemical formula	C_32_H_32_BrN_2_O_2_^+^·C_4_H_3_O_4_^−^	C_32_H_32_BrN_2_O_2_^+^·C_4_H_3_O_4_^−^
*M*_r_	671.56	671.56
Crystal system, space group	Monoclinic, *C*2	Monoclinic, *C*2
Temperature (K)	150	150
*a*, *b*, *c* (Å)	15.7494 (12), 13.3568 (11), 17.8634 (14)	15.7494 (12), 13.3568 (11), 17.8634 (14)
β (°)	106.500 (3)	106.500 (3)
*V* (Å^3^)	3603.0 (5)	3603.0 (5)
*Z*	4	4
Radiation type	Mo *K*α	Mo *K*α
μ (mm^−1^)	1.19	1.19
Crystal size (mm)	0.48 × 0.35 × 0.21	0.48 × 0.35 × 0.21

Data collection		
Diffractometer	Bruker AXS D8 Quest with PhotonII CPAD	Bruker AXS D8 Quest with PhotonII CPAD
Absorption correction	Multi-scan (*SADABS*; Krause *et al.*, 2015 ▸)	Multi-scan (*SADABS*; Krause *et al.*, 2015 ▸)
*T*_min_, *T*_max_	0.678, 0.740	0.678, 0.740
No. of measured, independent and observed [*I* > 2σ(*I*)] reflections	78980, 13641, 10348	78970, 13639, 10346
*R*_int_	0.049	0.049
(sin θ/λ)_max_ (Å^−1^)	0.769	0.769

Refinement		
*R*[*F* ^2^ > 2σ(*F* ^2^)], *wR*(*F* ^2^), *S*	0.048, 0.142, 1.06	0.042, 0.119, 1.06
No. of reflections	13641	13639
No. of parameters	418	418
No. of restraints	1	1
H-atom treatment	H atoms treated by a mixture of independent and constrained refinement	H atoms treated by a mixture of independent and constrained refinement
Δρ_max_, Δρ_min_ (e Å^−3^)	0.89, −0.74	0.76, −0.63
Absolute structure	Flack *x* determined using 4138 quotients [(*I* ^+^)−(*I* ^−^)]/[(*I* ^+^)+(*I* ^−^)] (Parsons *et al.*, 2013 ▸)	Flack *x* determined using 4139 quotients [(*I* ^+^)−(*I* ^−^)]/[(*I* ^+^)+(*I* ^−^)] (Parsons *et al.*, 2013 ▸)
Absolute structure parameter	0.044 (3)	0.044 (3)

Computer programs: *APEX3* and *SAINT* (Bruker, 2020 ▸), *SHELXS97* (Sheldrick, 2008 ▸), *SHELXT* (Sheldrick, 2015*a* ▸), *SHELXL2018/3* (Sheldrick, 2015*b* ▸), *shelXle* (Hübschle *et al.*, 2011 ▸), *Mercury* (Macrae *et al.*, 2020 ▸) and *publCIF* (Westrip, 2010 ▸).

## supplementary crystallographic information

### [4-(6-bromo-2-methoxyquinolin-3-yl)-3-hydroxy-3-(naphthalen-1-yl)-4-phenylbutyl]dimethylazanium 3-carboxyprop-2-enoate 0.476-hydrate (hemihydrate) Crystal data

**Table:**

C_32_H_32_BrN_2_O_2_^+^·C_4_H_3_O_4_^−^·0.476H_2_O	*F*(000) = 1411
*M**_r_* = 680.17	*D*_x_ = 1.262 Mg m^−^^3^
Monoclinic, *C*2	Cu *K*α radiation, λ = 1.54178 Å
*a* = 15.7469 (5) Å	Cell parameters from 9805 reflections
*b* = 13.2627 (4) Å	θ = 2.6–79.1°
*c* = 17.8602 (6) Å	µ = 1.94 mm^−^^1^
β = 106.3762 (13)°	*T* = 150 K
*V* = 3578.7 (2) Å^3^	Block, colourless
*Z* = 4	0.21 × 0.17 × 0.13 mm

### [4-(6-bromo-2-methoxyquinolin-3-yl)-3-hydroxy-3-(naphthalen-1-yl)-4-phenylbutyl]dimethylazanium 3-carboxyprop-2-enoate 0.476-hydrate (hemihydrate) Data collection

**Table:**

Bruker AXS D8 Quest with PhotonIII_C14 CPAD diffractometer	7003 independent reflections
Radiation source: I-mu-S microsource X-ray tube	6474 reflections with *I* > 2σ(*I*)
Laterally graded multilayer (Goebel) mirror monochromator	*R*_int_ = 0.040
Detector resolution: 7.4074 pixels mm^-1^	θ_max_ = 79.8°, θ_min_ = 2.6°
ω and phi scans	*h* = −19→19
Absorption correction: multi-scan (SADABS; Krause *et al.*, 2015)	*k* = −16→16
*T*_min_ = 0.646, *T*_max_ = 0.754	*l* = −18→22
15060 measured reflections	

### [4-(6-bromo-2-methoxyquinolin-3-yl)-3-hydroxy-3-(naphthalen-1-yl)-4-phenylbutyl]dimethylazanium 3-carboxyprop-2-enoate 0.476-hydrate (hemihydrate) Refinement

**Table:**

Refinement on *F*^2^	Hydrogen site location: mixed
Least-squares matrix: full	H atoms treated by a mixture of independent and constrained refinement
*R*[*F*^2^ > 2σ(*F*^2^)] = 0.041	*w* = 1/[σ^2^(*F*_o_^2^) + (0.0255*P*)^2^ + 3.0883*P*] where *P* = (*F*_o_^2^ + 2*F*_c_^2^)/3
*wR*(*F*^2^) = 0.107	(Δ/σ)_max_ < 0.001
*S* = 1.08	Δρ_max_ = 0.35 e Å^−^^3^
7003 reflections	Δρ_min_ = −0.46 e Å^−^^3^
430 parameters	Extinction correction: SHELXL2018/3 (Sheldrick, 2015b), Fc^*^=kFc[1+0.001xFc^2^λ^3^/sin(2θ)]^-1/4^
8 restraints	Extinction coefficient: 0.00103 (15)
Primary atom site location: structure-invariant direct methods	Absolute structure: Flack *x* determined using 2633 quotients [(*I*^+^)-(*I*^-^)]/[(*I*^+^)+(*I*^-^)] (Parsons *et al.*, 2013)
Secondary atom site location: difference Fourier map	Absolute structure parameter: 0.019 (8)

### [4-(6-bromo-2-methoxyquinolin-3-yl)-3-hydroxy-3-(naphthalen-1-yl)-4-phenylbutyl]dimethylazanium 3-carboxyprop-2-enoate 0.476-hydrate (hemihydrate) Special details

**Table:**

Geometry. All esds (except the esd in the dihedral angle between two l.s. planes) are estimated using the full covariance matrix. The cell esds are taken into account individually in the estimation of esds in distances, angles and torsion angles; correlations between esds in cell parameters are only used when they are defined by crystal symmetry. An approximate (isotropic) treatment of cell esds is used for estimating esds involving l.s. planes.
Refinement. The position of the hydromaleate acidic hydrogen atom was freely refined. Two partially occupied water molecules are situated in the asymmetric part of the unit cell. One in a general position, the other located on a twofold axis. They are hydrogen bonded to each other, and the one ion the general position is also H-bonded to O3 of the hydromaleate anion. Water H atom positions were refined and O-H and H···H distances were restrained to 0.84 (2) and 1.36 (2) Angstrom, respectively, and H atom positions were further restrained based on hydrogen bonding considerations. A damping factor was applied during refinement. In the final refinement cycles the damping factor was removed and the water H atoms were set to ride on their carrier oxygen atoms. Subject to these conditions the occupancy rates refined to 0.276 (17) for O7 (general position) and 0.40 (4) for O8 (twofold axis). Additional solvent accessible space is present in the crystal lattice (two times 123 cubic Angstrom, or 6.9% of the unit cell volume). No electron density was found inside the void space (A Platon Squeeze analysis corrected for 8 electrons in the combined void space), and the content of the void space was ignored.

### [4-(6-bromo-2-methoxyquinolin-3-yl)-3-hydroxy-3-(naphthalen-1-yl)-4-phenylbutyl]dimethylazanium 3-carboxyprop-2-enoate 0.476-hydrate (hemihydrate) Fractional atomic coordinates and isotropic or equivalent isotropic displacement parameters (Å^2^)

**Table:**

	*x*	*y*	*z*	*U*_iso_*/*U*_eq_	Occ. (<1)
Br1	0.30375 (5)	0.01666 (6)	0.48115 (4)	0.0839 (3)	
O1	0.48764 (16)	0.27255 (18)	0.16376 (15)	0.0333 (5)	
H1O	0.440678	0.288534	0.174294	0.040*	
O2	0.56686 (17)	0.48680 (18)	0.36191 (15)	0.0366 (6)	
O3	0.48874 (18)	0.6977 (2)	0.21055 (18)	0.0454 (7)	
O4	0.5661 (2)	0.7107 (3)	0.12579 (16)	0.0555 (9)	
O5	0.7100 (2)	0.7702 (3)	0.12234 (17)	0.0541 (8)	
H5	0.642 (4)	0.739 (4)	0.118 (3)	0.065*	
O6	0.83376 (18)	0.8188 (2)	0.20818 (18)	0.0459 (7)	
N1	0.39765 (18)	0.5691 (2)	0.09891 (17)	0.0289 (6)	
H1	0.430917	0.620889	0.136586	0.035*	
N2	0.46061 (19)	0.4070 (3)	0.40605 (17)	0.0343 (7)	
C1	0.6050 (2)	0.3244 (3)	0.27938 (19)	0.0298 (7)	
H1A	0.643444	0.384240	0.299018	0.036*	
C2	0.5524 (2)	0.3481 (3)	0.1918 (2)	0.0289 (7)	
C3	0.5040 (2)	0.4505 (3)	0.18735 (18)	0.0278 (7)	
H3A	0.459479	0.445909	0.216773	0.033*	
H3B	0.547308	0.503311	0.212095	0.033*	
C4	0.4581 (2)	0.4805 (3)	0.10285 (19)	0.0296 (7)	
H4A	0.503360	0.497710	0.076116	0.036*	
H4B	0.423422	0.422623	0.075196	0.036*	
C5	0.3164 (2)	0.5436 (3)	0.1216 (2)	0.0381 (8)	
H5A	0.281406	0.494176	0.084779	0.057*	
H5B	0.281123	0.604731	0.120611	0.057*	
H5C	0.332940	0.515003	0.174331	0.057*	
C6	0.3746 (2)	0.6148 (3)	0.0197 (2)	0.0356 (8)	
H6A	0.348869	0.563192	−0.019349	0.053*	
H6B	0.428209	0.642151	0.009674	0.053*	
H6C	0.331672	0.669221	0.016597	0.053*	
C7	0.6162 (2)	0.3460 (3)	0.14034 (19)	0.0295 (7)	
C8	0.5999 (3)	0.2769 (3)	0.0803 (2)	0.0359 (8)	
H8	0.550478	0.233013	0.072570	0.043*	
C9	0.6545 (3)	0.2695 (3)	0.0299 (2)	0.0423 (9)	
H9	0.640299	0.222296	−0.011847	0.051*	
C10	0.7265 (3)	0.3288 (3)	0.0404 (2)	0.0430 (9)	
H10	0.763079	0.322213	0.006498	0.052*	
C11	0.7480 (3)	0.4006 (3)	0.1014 (2)	0.0401 (9)	
C12	0.8229 (3)	0.4631 (4)	0.1120 (3)	0.0544 (11)	
H12	0.859811	0.454499	0.078529	0.065*	
C13	0.8443 (3)	0.5352 (4)	0.1683 (3)	0.0560 (12)	
H13	0.894833	0.576837	0.173912	0.067*	
C14	0.7905 (3)	0.5467 (3)	0.2176 (3)	0.0471 (10)	
H14	0.804887	0.596659	0.257288	0.056*	
C15	0.7170 (2)	0.4875 (3)	0.2103 (2)	0.0375 (8)	
H15	0.681837	0.497728	0.245051	0.045*	
C16	0.6919 (2)	0.4111 (3)	0.1521 (2)	0.0316 (7)	
C17	0.6665 (2)	0.2338 (3)	0.2887 (2)	0.0340 (7)	
C18	0.6427 (3)	0.1422 (3)	0.2516 (2)	0.0415 (9)	
H18	0.586140	0.135021	0.214926	0.050*	
C19	0.7002 (3)	0.0608 (4)	0.2671 (3)	0.0558 (12)	
H19	0.682897	−0.001380	0.240840	0.067*	
C20	0.7823 (4)	0.0694 (4)	0.3204 (3)	0.0641 (14)	
H20	0.821473	0.013425	0.331189	0.077*	
C21	0.8069 (3)	0.1597 (5)	0.3579 (3)	0.0659 (15)	
H21	0.863077	0.165785	0.395277	0.079*	
C22	0.7503 (3)	0.2422 (4)	0.3417 (2)	0.0476 (10)	
H22	0.768804	0.304756	0.366877	0.057*	
C23	0.5424 (2)	0.3152 (3)	0.33007 (19)	0.0301 (7)	
C24	0.5058 (2)	0.2271 (3)	0.34449 (19)	0.0321 (7)	
H24	0.520783	0.166081	0.323320	0.039*	
C25	0.4456 (2)	0.2245 (3)	0.3907 (2)	0.0361 (8)	
C26	0.4097 (3)	0.1350 (4)	0.4095 (2)	0.0434 (9)	
H26	0.426466	0.071968	0.392785	0.052*	
C27	0.3499 (3)	0.1392 (4)	0.4524 (2)	0.0511 (11)	
C28	0.3230 (3)	0.2303 (4)	0.4767 (2)	0.0511 (11)	
H28	0.280267	0.231305	0.505079	0.061*	
C29	0.3584 (3)	0.3182 (4)	0.4597 (2)	0.0441 (10)	
H29	0.339894	0.380429	0.476275	0.053*	
C30	0.4221 (2)	0.3183 (3)	0.4178 (2)	0.0359 (8)	
C31	0.5198 (2)	0.4035 (3)	0.36758 (19)	0.0309 (7)	
C32	0.5519 (3)	0.5748 (3)	0.4034 (3)	0.0503 (10)	
H32A	0.493257	0.602628	0.377597	0.075*	
H32B	0.597264	0.625486	0.403543	0.075*	
H32C	0.554939	0.556447	0.457197	0.075*	
C33	0.5569 (2)	0.7229 (3)	0.1938 (2)	0.0363 (8)	
C34	0.6313 (3)	0.7650 (3)	0.2571 (2)	0.0377 (8)	
H34	0.618840	0.773706	0.305769	0.045*	
C35	0.7131 (2)	0.7926 (3)	0.2566 (2)	0.0369 (8)	
H35	0.749826	0.815444	0.305430	0.044*	
C36	0.7555 (3)	0.7933 (3)	0.1923 (2)	0.0372 (8)	
O7	0.4482 (13)	0.8469 (14)	0.3326 (10)	0.106 (9)	0.276 (17)
H7A	0.461417	0.803439	0.303738	0.159*	0.276 (17)
H7B	0.477841	0.899199	0.331524	0.159*	0.276 (17)
O8	0.500000	0.777 (3)	0.500000	0.30 (4)	0.40 (4)
H8A	0.488152	0.815245	0.454344	0.443*	0.40 (4)

### [4-(6-bromo-2-methoxyquinolin-3-yl)-3-hydroxy-3-(naphthalen-1-yl)-4-phenylbutyl]dimethylazanium 3-carboxyprop-2-enoate 0.476-hydrate (hemihydrate) Atomic displacement parameters (Å^2^)

**Table:**

	*U*^11^	*U*^22^	*U*^33^	*U*^12^	*U*^13^	*U*^23^
Br1	0.0824 (4)	0.0920 (5)	0.0923 (5)	−0.0330 (3)	0.0492 (4)	0.0030 (4)
O1	0.0317 (11)	0.0322 (12)	0.0336 (12)	0.0002 (10)	0.0051 (10)	−0.0007 (10)
O2	0.0427 (13)	0.0362 (13)	0.0312 (13)	0.0063 (10)	0.0106 (11)	−0.0011 (10)
O3	0.0404 (14)	0.0510 (16)	0.0496 (16)	−0.0108 (13)	0.0203 (13)	−0.0087 (13)
O4	0.0459 (15)	0.089 (2)	0.0337 (14)	−0.0255 (16)	0.0139 (12)	−0.0177 (16)
O5	0.0492 (16)	0.082 (2)	0.0334 (14)	−0.0239 (16)	0.0148 (12)	−0.0089 (15)
O6	0.0356 (13)	0.0453 (15)	0.0565 (17)	−0.0048 (12)	0.0127 (12)	0.0015 (14)
N1	0.0260 (13)	0.0298 (13)	0.0302 (14)	0.0031 (11)	0.0070 (11)	0.0032 (12)
N2	0.0307 (14)	0.0485 (18)	0.0224 (13)	0.0099 (13)	0.0054 (11)	−0.0009 (13)
C1	0.0298 (15)	0.0347 (17)	0.0240 (15)	0.0022 (13)	0.0060 (13)	0.0035 (14)
C2	0.0278 (15)	0.0303 (16)	0.0275 (16)	0.0045 (12)	0.0056 (13)	−0.0004 (13)
C3	0.0282 (15)	0.0314 (15)	0.0231 (15)	0.0043 (13)	0.0062 (12)	0.0011 (13)
C4	0.0320 (16)	0.0317 (16)	0.0247 (15)	0.0066 (13)	0.0072 (13)	0.0016 (13)
C5	0.0270 (16)	0.044 (2)	0.045 (2)	0.0047 (14)	0.0143 (15)	0.0101 (17)
C6	0.0380 (18)	0.0365 (18)	0.0315 (17)	0.0084 (15)	0.0085 (15)	0.0090 (16)
C7	0.0342 (16)	0.0324 (17)	0.0222 (15)	0.0123 (13)	0.0083 (13)	0.0030 (13)
C8	0.0397 (18)	0.0392 (18)	0.0273 (16)	0.0089 (16)	0.0070 (14)	−0.0010 (15)
C9	0.057 (2)	0.044 (2)	0.0264 (17)	0.0229 (19)	0.0123 (16)	0.0017 (16)
C10	0.052 (2)	0.044 (2)	0.040 (2)	0.0228 (18)	0.0229 (18)	0.0118 (18)
C11	0.0384 (18)	0.046 (2)	0.042 (2)	0.0151 (16)	0.0202 (16)	0.0127 (17)
C12	0.045 (2)	0.061 (3)	0.065 (3)	0.012 (2)	0.029 (2)	0.018 (2)
C13	0.040 (2)	0.057 (3)	0.075 (3)	−0.0041 (19)	0.023 (2)	0.007 (3)
C14	0.0392 (19)	0.045 (2)	0.055 (2)	−0.0032 (16)	0.0099 (18)	0.0038 (19)
C15	0.0373 (18)	0.0398 (19)	0.0357 (19)	0.0027 (15)	0.0108 (15)	0.0031 (16)
C16	0.0306 (16)	0.0357 (17)	0.0293 (16)	0.0075 (14)	0.0095 (13)	0.0040 (14)
C17	0.0342 (17)	0.0414 (19)	0.0275 (16)	0.0115 (15)	0.0105 (14)	0.0094 (16)
C18	0.047 (2)	0.0392 (19)	0.043 (2)	0.0153 (17)	0.0190 (17)	0.0099 (17)
C19	0.062 (3)	0.044 (2)	0.069 (3)	0.021 (2)	0.031 (2)	0.011 (2)
C20	0.066 (3)	0.070 (3)	0.063 (3)	0.044 (3)	0.029 (2)	0.023 (3)
C21	0.044 (2)	0.099 (4)	0.054 (3)	0.035 (3)	0.011 (2)	0.018 (3)
C22	0.0377 (19)	0.066 (3)	0.0372 (19)	0.0149 (19)	0.0075 (16)	0.006 (2)
C23	0.0257 (15)	0.0408 (18)	0.0225 (15)	0.0070 (14)	0.0045 (12)	0.0018 (14)
C24	0.0319 (16)	0.0405 (19)	0.0247 (15)	0.0064 (15)	0.0092 (13)	0.0016 (15)
C25	0.0291 (16)	0.053 (2)	0.0238 (15)	0.0030 (16)	0.0038 (13)	0.0018 (16)
C26	0.0382 (19)	0.056 (2)	0.0350 (19)	−0.0059 (18)	0.0094 (16)	0.0017 (19)
C27	0.040 (2)	0.079 (3)	0.036 (2)	−0.019 (2)	0.0122 (17)	0.002 (2)
C28	0.0354 (19)	0.089 (3)	0.0308 (18)	−0.010 (2)	0.0126 (16)	−0.004 (2)
C29	0.0325 (18)	0.074 (3)	0.0251 (17)	0.0032 (18)	0.0075 (14)	−0.0065 (19)
C30	0.0286 (16)	0.056 (2)	0.0199 (15)	0.0046 (16)	0.0025 (13)	−0.0013 (16)
C31	0.0303 (16)	0.0412 (18)	0.0203 (15)	0.0076 (14)	0.0057 (13)	0.0024 (14)
C32	0.055 (2)	0.041 (2)	0.055 (3)	0.0068 (19)	0.016 (2)	−0.009 (2)
C33	0.0380 (18)	0.0346 (18)	0.0379 (18)	−0.0070 (15)	0.0133 (15)	−0.0048 (16)
C34	0.0439 (19)	0.0418 (19)	0.0281 (17)	−0.0080 (16)	0.0114 (15)	−0.0054 (15)
C35	0.0365 (18)	0.042 (2)	0.0298 (17)	−0.0049 (15)	0.0059 (15)	−0.0035 (16)
C36	0.0394 (18)	0.0325 (18)	0.040 (2)	−0.0046 (15)	0.0117 (16)	0.0009 (15)
O7	0.109 (15)	0.086 (13)	0.146 (19)	0.007 (10)	0.073 (14)	0.001 (12)
O8	0.32 (7)	0.14 (3)	0.33 (7)	0.000	−0.07 (5)	0.000

### [4-(6-bromo-2-methoxyquinolin-3-yl)-3-hydroxy-3-(naphthalen-1-yl)-4-phenylbutyl]dimethylazanium 3-carboxyprop-2-enoate 0.476-hydrate (hemihydrate) Geometric parameters (Å, º)

**Table:**

Br1—C27	1.909 (5)	C12—C13	1.360 (8)
O1—C2	1.417 (4)	C12—H12	0.9500
O1—H1O	0.8400	C13—C14	1.392 (7)
O2—C31	1.349 (5)	C13—H13	0.9500
O2—C32	1.437 (5)	C14—C15	1.374 (6)
O3—C33	1.237 (5)	C14—H14	0.9500
O4—C33	1.275 (5)	C15—C16	1.425 (5)
O4—H5	1.29 (6)	C15—H15	0.9500
O5—C36	1.290 (5)	C17—C18	1.383 (6)
O5—H5	1.13 (6)	C17—C22	1.395 (6)
O6—C36	1.231 (5)	C18—C19	1.386 (6)
N1—C5	1.487 (4)	C18—H18	0.9500
N1—C6	1.487 (5)	C19—C20	1.376 (8)
N1—C4	1.501 (4)	C19—H19	0.9500
N1—H1	1.0000	C20—C21	1.373 (9)
N2—C31	1.306 (5)	C20—H20	0.9500
N2—C30	1.366 (5)	C21—C22	1.388 (7)
C1—C23	1.520 (5)	C21—H21	0.9500
C1—C17	1.523 (5)	C22—H22	0.9500
C1—C2	1.581 (5)	C23—C24	1.359 (5)
C1—H1A	1.0000	C23—C31	1.443 (5)
C2—C7	1.541 (5)	C24—C25	1.423 (5)
C2—C3	1.548 (5)	C24—H24	0.9500
C3—C4	1.531 (4)	C25—C26	1.395 (6)
C3—H3A	0.9900	C25—C30	1.420 (6)
C3—H3B	0.9900	C26—C27	1.372 (6)
C4—H4A	0.9900	C26—H26	0.9500
C4—H4B	0.9900	C27—C28	1.390 (8)
C5—H5A	0.9800	C28—C29	1.363 (7)
C5—H5B	0.9800	C28—H28	0.9500
C5—H5C	0.9800	C29—C30	1.410 (5)
C6—H6A	0.9800	C29—H29	0.9500
C6—H6B	0.9800	C32—H32A	0.9800
C6—H6C	0.9800	C32—H32B	0.9800
C7—C8	1.379 (5)	C32—H32C	0.9800
C7—C16	1.439 (5)	C33—C34	1.488 (5)
C8—C9	1.413 (5)	C34—C35	1.342 (5)
C8—H8	0.9500	C34—H34	0.9500
C9—C10	1.349 (7)	C35—C36	1.482 (6)
C9—H9	0.9500	C35—H35	0.9500
C10—C11	1.416 (6)	O7—H7A	0.8370
C10—H10	0.9500	O7—H7B	0.8394
C11—C12	1.411 (7)	O8—H8A	0.9355
C11—C16	1.438 (5)	O8—H8A^i^	0.9355
			
C2—O1—H1O	109.5	C14—C15—C16	121.9 (4)
C31—O2—C32	117.0 (3)	C14—C15—H15	119.1
C33—O4—H5	115 (3)	C16—C15—H15	119.1
C36—O5—H5	114 (3)	C15—C16—C11	115.8 (3)
C5—N1—C6	110.8 (3)	C15—C16—C7	125.5 (3)
C5—N1—C4	113.1 (3)	C11—C16—C7	118.7 (3)
C6—N1—C4	110.2 (3)	C18—C17—C22	118.2 (4)
C5—N1—H1	107.5	C18—C17—C1	124.2 (3)
C6—N1—H1	107.5	C22—C17—C1	117.5 (4)
C4—N1—H1	107.5	C17—C18—C19	121.0 (4)
C31—N2—C30	117.7 (3)	C17—C18—H18	119.5
C23—C1—C17	111.8 (3)	C19—C18—H18	119.5
C23—C1—C2	111.0 (3)	C20—C19—C18	120.4 (5)
C17—C1—C2	113.9 (3)	C20—C19—H19	119.8
C23—C1—H1A	106.6	C18—C19—H19	119.8
C17—C1—H1A	106.6	C21—C20—C19	119.4 (4)
C2—C1—H1A	106.6	C21—C20—H20	120.3
O1—C2—C7	107.5 (3)	C19—C20—H20	120.3
O1—C2—C3	107.9 (3)	C20—C21—C22	120.6 (5)
C7—C2—C3	112.3 (3)	C20—C21—H21	119.7
O1—C2—C1	109.5 (3)	C22—C21—H21	119.7
C7—C2—C1	109.8 (3)	C21—C22—C17	120.5 (5)
C3—C2—C1	109.8 (3)	C21—C22—H22	119.8
C4—C3—C2	111.6 (3)	C17—C22—H22	119.8
C4—C3—H3A	109.3	C24—C23—C31	115.9 (3)
C2—C3—H3A	109.3	C24—C23—C1	124.2 (3)
C4—C3—H3B	109.3	C31—C23—C1	119.8 (3)
C2—C3—H3B	109.3	C23—C24—C25	121.2 (3)
H3A—C3—H3B	108.0	C23—C24—H24	119.4
N1—C4—C3	111.5 (3)	C25—C24—H24	119.4
N1—C4—H4A	109.3	C26—C25—C30	120.1 (3)
C3—C4—H4A	109.3	C26—C25—C24	122.8 (4)
N1—C4—H4B	109.3	C30—C25—C24	117.2 (4)
C3—C4—H4B	109.3	C27—C26—C25	119.2 (4)
H4A—C4—H4B	108.0	C27—C26—H26	120.4
N1—C5—H5A	109.5	C25—C26—H26	120.4
N1—C5—H5B	109.5	C26—C27—C28	121.8 (4)
H5A—C5—H5B	109.5	C26—C27—Br1	119.3 (4)
N1—C5—H5C	109.5	C28—C27—Br1	118.9 (3)
H5A—C5—H5C	109.5	C29—C28—C27	119.6 (4)
H5B—C5—H5C	109.5	C29—C28—H28	120.2
N1—C6—H6A	109.5	C27—C28—H28	120.2
N1—C6—H6B	109.5	C28—C29—C30	121.0 (4)
H6A—C6—H6B	109.5	C28—C29—H29	119.5
N1—C6—H6C	109.5	C30—C29—H29	119.5
H6A—C6—H6C	109.5	N2—C30—C29	119.6 (4)
H6B—C6—H6C	109.5	N2—C30—C25	122.2 (3)
C8—C7—C16	118.5 (3)	C29—C30—C25	118.2 (4)
C8—C7—C2	117.9 (3)	N2—C31—O2	120.0 (3)
C16—C7—C2	123.7 (3)	N2—C31—C23	125.3 (4)
C7—C8—C9	122.0 (4)	O2—C31—C23	114.7 (3)
C7—C8—H8	119.0	O2—C32—H32A	109.5
C9—C8—H8	119.0	O2—C32—H32B	109.5
C10—C9—C8	120.6 (4)	H32A—C32—H32B	109.5
C10—C9—H9	119.7	O2—C32—H32C	109.5
C8—C9—H9	119.7	H32A—C32—H32C	109.5
C9—C10—C11	120.6 (4)	H32B—C32—H32C	109.5
C9—C10—H10	119.7	O3—C33—O4	122.4 (3)
C11—C10—H10	119.7	O3—C33—C34	117.9 (3)
C12—C11—C10	120.5 (4)	O4—C33—C34	119.6 (3)
C12—C11—C16	119.9 (4)	C35—C34—C33	130.5 (3)
C10—C11—C16	119.6 (4)	C35—C34—H34	114.8
C13—C12—C11	122.4 (4)	C33—C34—H34	114.8
C13—C12—H12	118.8	C34—C35—C36	130.6 (3)
C11—C12—H12	118.8	C34—C35—H35	114.7
C12—C13—C14	118.5 (4)	C36—C35—H35	114.7
C12—C13—H13	120.8	O6—C36—O5	122.3 (4)
C14—C13—H13	120.8	O6—C36—C35	117.6 (4)
C15—C14—C13	121.7 (4)	O5—C36—C35	120.0 (3)
C15—C14—H14	119.2	H7A—O7—H7B	108.6
C13—C14—H14	119.2	H8A—O8—H8A^i^	113.8
			
C23—C1—C2—O1	−56.3 (4)	C1—C17—C18—C19	−175.4 (4)
C17—C1—C2—O1	70.9 (4)	C17—C18—C19—C20	0.4 (7)
C23—C1—C2—C7	−174.1 (3)	C18—C19—C20—C21	−0.3 (8)
C17—C1—C2—C7	−46.9 (4)	C19—C20—C21—C22	−0.9 (8)
C23—C1—C2—C3	62.0 (4)	C20—C21—C22—C17	1.9 (7)
C17—C1—C2—C3	−170.8 (3)	C18—C17—C22—C21	−1.7 (6)
O1—C2—C3—C4	−65.2 (4)	C1—C17—C22—C21	174.5 (4)
C7—C2—C3—C4	53.1 (4)	C17—C1—C23—C24	−36.4 (4)
C1—C2—C3—C4	175.6 (3)	C2—C1—C23—C24	91.9 (4)
C5—N1—C4—C3	−71.8 (4)	C17—C1—C23—C31	142.4 (3)
C6—N1—C4—C3	163.7 (3)	C2—C1—C23—C31	−89.3 (4)
C2—C3—C4—N1	169.4 (3)	C31—C23—C24—C25	3.1 (5)
O1—C2—C7—C8	0.5 (4)	C1—C23—C24—C25	−178.1 (3)
C3—C2—C7—C8	−118.0 (3)	C23—C24—C25—C26	−177.2 (3)
C1—C2—C7—C8	119.5 (3)	C23—C24—C25—C30	3.0 (5)
O1—C2—C7—C16	−178.8 (3)	C30—C25—C26—C27	1.8 (5)
C3—C2—C7—C16	62.7 (4)	C24—C25—C26—C27	−178.0 (4)
C1—C2—C7—C16	−59.8 (4)	C25—C26—C27—C28	0.9 (6)
C16—C7—C8—C9	−0.9 (5)	C25—C26—C27—Br1	−177.9 (3)
C2—C7—C8—C9	179.8 (3)	C26—C27—C28—C29	−1.8 (6)
C7—C8—C9—C10	1.8 (6)	Br1—C27—C28—C29	177.0 (3)
C8—C9—C10—C11	−1.0 (6)	C27—C28—C29—C30	−0.1 (6)
C9—C10—C11—C12	−179.5 (4)	C31—N2—C30—C29	179.4 (3)
C9—C10—C11—C16	−0.6 (6)	C31—N2—C30—C25	1.0 (5)
C10—C11—C12—C13	177.9 (4)	C28—C29—C30—N2	−175.7 (3)
C16—C11—C12—C13	−1.0 (7)	C28—C29—C30—C25	2.8 (5)
C11—C12—C13—C14	0.7 (7)	C26—C25—C30—N2	174.9 (3)
C12—C13—C14—C15	−0.2 (7)	C24—C25—C30—N2	−5.3 (5)
C13—C14—C15—C16	0.1 (6)	C26—C25—C30—C29	−3.6 (5)
C14—C15—C16—C11	−0.5 (5)	C24—C25—C30—C29	176.2 (3)
C14—C15—C16—C7	−179.9 (4)	C30—N2—C31—O2	−172.6 (3)
C12—C11—C16—C15	0.9 (5)	C30—N2—C31—C23	6.0 (5)
C10—C11—C16—C15	−178.0 (3)	C32—O2—C31—N2	3.5 (5)
C12—C11—C16—C7	−179.6 (4)	C32—O2—C31—C23	−175.3 (3)
C10—C11—C16—C7	1.4 (5)	C24—C23—C31—N2	−8.1 (5)
C8—C7—C16—C15	178.7 (3)	C1—C23—C31—N2	173.0 (3)
C2—C7—C16—C15	−2.0 (5)	C24—C23—C31—O2	170.6 (3)
C8—C7—C16—C11	−0.7 (5)	C1—C23—C31—O2	−8.3 (4)
C2—C7—C16—C11	178.6 (3)	O3—C33—C34—C35	175.8 (4)
C23—C1—C17—C18	80.4 (4)	O4—C33—C34—C35	−1.8 (7)
C2—C1—C17—C18	−46.3 (5)	C33—C34—C35—C36	2.2 (8)
C23—C1—C17—C22	−95.5 (4)	C34—C35—C36—O6	−177.1 (4)
C2—C1—C17—C22	137.7 (3)	C34—C35—C36—O5	4.6 (7)
C22—C17—C18—C19	0.5 (6)		

Symmetry code: (i) −*x*+1, *y*, −*z*+1.

### [4-(6-bromo-2-methoxyquinolin-3-yl)-3-hydroxy-3-(naphthalen-1-yl)-4-phenylbutyl]dimethylazanium 3-carboxyprop-2-enoate 0.476-hydrate (hemihydrate) Hydrogen-bond geometry (Å, º)

**Table:**

*D*—H···*A*	*D*—H	H···*A*	*D*···*A*	*D*—H···*A*
O1—H1*O*···O6^ii^	0.84	1.98	2.822 (4)	175
O5—H5···O4	1.13 (6)	1.29 (6)	2.417 (4)	169 (5)
N1—H1···O3	1.00	1.71	2.707 (4)	172
N1—H1···O4	1.00	2.49	3.174 (4)	125
C3—H3*A*···O6^ii^	0.99	2.57	3.308 (4)	131
C6—H6*B*···O4^iii^	0.98	2.61	3.257 (5)	124
O7—H7*A*···O3	0.84	2.31	3.139 (16)	172
O8—H8*A*···O7	0.94	2.13	3.02 (2)	158

Symmetry codes: (ii) *x*−1/2, *y*−1/2, *z*; (iii) −*x*+1, *y*, −*z*.

### [4-(6-bromo-2-methoxyquinolin-3-yl)-3-hydroxy-3-(naphthalen-1-yl)-4-phenylbutyl]dimethylazanium 3-carboxyprop-2-enoate tetrahydrofuran sesquihydrate (THF) Crystal data

**Table:**

2C_32_H_32_BrN_2_O_2_^+^·2C_4_H_3_O_4_^−^·3C_4_H_8_O	*F*(000) = 1632
*M**_r_* = 1559.45	*D*_x_ = 1.365 Mg m^−^^3^
Monoclinic, *C*2	Cu *K*α radiation, λ = 1.54178 Å
*a* = 16.4119 (6) Å	Cell parameters from 9944 reflections
*b* = 13.5643 (6) Å	θ = 2.6–78.6°
*c* = 17.8475 (8) Å	µ = 1.92 mm^−^^1^
β = 107.318 (3)°	*T* = 150 K
*V* = 3793.0 (3) Å^3^	Fragment, colourless
*Z* = 2	0.33 × 0.19 × 0.16 mm

### [4-(6-bromo-2-methoxyquinolin-3-yl)-3-hydroxy-3-(naphthalen-1-yl)-4-phenylbutyl]dimethylazanium 3-carboxyprop-2-enoate tetrahydrofuran sesquihydrate (THF) Data collection

**Table:**

Bruker AXS D8 Quest with PhotonIII_C14 CPAD diffractometer	7473 independent reflections
Radiation source: I-mu-S microsource X-ray tube	6615 reflections with *I* > 2σ(*I*)
Laterally graded multilayer (Goebel) mirror monochromator	*R*_int_ = 0.049
Detector resolution: 7.4074 pixels mm^-1^	θ_max_ = 79.9°, θ_min_ = 2.6°
ω and phi scans	*h* = −18→20
Absorption correction: multi-scan (SADABS; Krause *et al.*, 2015)	*k* = −16→17
*T*_min_ = 0.254, *T*_max_ = 0.391	*l* = −22→21
18660 measured reflections	

### [4-(6-bromo-2-methoxyquinolin-3-yl)-3-hydroxy-3-(naphthalen-1-yl)-4-phenylbutyl]dimethylazanium 3-carboxyprop-2-enoate tetrahydrofuran sesquihydrate (THF) Refinement

**Table:**

Refinement on *F*^2^	Hydrogen site location: mixed
Least-squares matrix: full	H atoms treated by a mixture of independent and constrained refinement
*R*[*F*^2^ > 2σ(*F*^2^)] = 0.054	*w* = 1/[σ^2^(*F*_o_^2^) + 6.5774*P*] where *P* = (*F*_o_^2^ + 2*F*_c_^2^)/3
*wR*(*F*^2^) = 0.127	(Δ/σ)_max_ = 0.001
*S* = 1.05	Δρ_max_ = 0.46 e Å^−^^3^
7473 reflections	Δρ_min_ = −0.52 e Å^−^^3^
551 parameters	Extinction correction: SHELXL2018/3 (Sheldrick, 2015b), Fc^*^=kFc[1+0.001xFc^2^λ^3^/sin(2θ)]^-1/4^
223 restraints	Extinction coefficient: 0.00079 (10)
Primary atom site location: isomorphous structure methods	Absolute structure: Refined as an inversion twin
Secondary atom site location: difference Fourier map	Absolute structure parameter: 0.03 (3)

### [4-(6-bromo-2-methoxyquinolin-3-yl)-3-hydroxy-3-(naphthalen-1-yl)-4-phenylbutyl]dimethylazanium 3-carboxyprop-2-enoate tetrahydrofuran sesquihydrate (THF) Special details

**Table:**

Geometry. All esds (except the esd in the dihedral angle between two l.s. planes) are estimated using the full covariance matrix. The cell esds are taken into account individually in the estimation of esds in distances, angles and torsion angles; correlations between esds in cell parameters are only used when they are defined by crystal symmetry. An approximate (isotropic) treatment of cell esds is used for estimating esds involving l.s. planes.
Refinement. Refined as a two-component inversion twinThe position of the hydromaleate acidic hydrogen atom was freely refined. The structure was solved from its 0.5 hydrate analogue by isomorphous replacement. Two THF molecules were refined as disordered. One in a 1:1 ratio around a two-fold axis, the other in a general position. The three disordered moieties were restrained to have similar geometries. Uij components of ADPs for disordered atoms closer to each other than 2.0 Angstrom were restrained to be similar. Subject to these conditions the occupancy ratio for the molecule in the general position refined to 0.587 (16) to 0.413 (16).

### [4-(6-bromo-2-methoxyquinolin-3-yl)-3-hydroxy-3-(naphthalen-1-yl)-4-phenylbutyl]dimethylazanium 3-carboxyprop-2-enoate tetrahydrofuran sesquihydrate (THF) Fractional atomic coordinates and isotropic or equivalent isotropic displacement parameters (Å^2^)

**Table:**

	*x*	*y*	*z*	*U*_iso_*/*U*_eq_	Occ. (<1)
Br1	0.26058 (6)	0.03336 (7)	0.44125 (5)	0.0900 (4)	
O1	0.4801 (2)	0.2596 (2)	0.1676 (2)	0.0393 (8)	
H1O	0.435393	0.274903	0.178628	0.047*	
O2	0.5566 (2)	0.4767 (3)	0.3637 (2)	0.0431 (8)	
O3	0.5052 (2)	0.6774 (3)	0.2100 (2)	0.0474 (9)	
O4	0.5714 (3)	0.6982 (4)	0.1204 (2)	0.0562 (11)	
O5	0.7069 (3)	0.7612 (4)	0.1131 (2)	0.0580 (11)	
H5	0.636 (5)	0.732 (6)	0.121 (4)	0.087*	
O6	0.8266 (2)	0.8159 (3)	0.1967 (3)	0.0542 (10)	
N1	0.4013 (2)	0.5583 (3)	0.1018 (2)	0.0354 (9)	
H1	0.438273	0.605699	0.139973	0.042*	
N2	0.4546 (3)	0.4009 (3)	0.4074 (2)	0.0393 (9)	
C1	0.5937 (3)	0.3139 (4)	0.2833 (3)	0.0346 (10)	
H1A	0.629984	0.373327	0.302215	0.042*	
C2	0.5429 (3)	0.3346 (3)	0.1955 (3)	0.0347 (10)	
C3	0.4961 (3)	0.4347 (4)	0.1881 (3)	0.0347 (10)	
H3A	0.452873	0.430504	0.216628	0.042*	
H3B	0.537920	0.485963	0.214137	0.042*	
C4	0.4524 (3)	0.4665 (4)	0.1047 (3)	0.0375 (11)	
H4A	0.495996	0.478198	0.077433	0.045*	
H4B	0.414420	0.413029	0.076740	0.045*	
C5	0.3232 (3)	0.5425 (5)	0.1264 (4)	0.0515 (14)	
H5A	0.288180	0.490667	0.093991	0.077*	
H5B	0.290307	0.603851	0.119812	0.077*	
H5C	0.339613	0.522452	0.181676	0.077*	
C6	0.3793 (4)	0.6057 (4)	0.0235 (3)	0.0452 (12)	
H6A	0.348841	0.558375	−0.016607	0.068*	
H6B	0.431630	0.626946	0.012547	0.068*	
H6C	0.342718	0.663002	0.022805	0.068*	
C7	0.6027 (3)	0.3295 (4)	0.1431 (3)	0.0356 (10)	
C8	0.5881 (4)	0.2595 (4)	0.0854 (3)	0.0423 (12)	
H8	0.540722	0.216667	0.078641	0.051*	
C9	0.6398 (4)	0.2478 (5)	0.0356 (3)	0.0514 (15)	
H9	0.627396	0.197259	−0.002969	0.062*	
C10	0.7074 (4)	0.3088 (5)	0.0429 (3)	0.0525 (15)	
H10	0.741843	0.301365	0.008919	0.063*	
C11	0.7262 (3)	0.3826 (5)	0.1004 (3)	0.0454 (13)	
C12	0.7952 (4)	0.4487 (5)	0.1066 (4)	0.0577 (16)	
H12	0.828900	0.440670	0.071970	0.069*	
C13	0.8147 (4)	0.5224 (6)	0.1599 (4)	0.0598 (16)	
H13	0.861375	0.565156	0.162841	0.072*	
C14	0.7653 (3)	0.5345 (5)	0.2101 (3)	0.0498 (13)	
H14	0.778767	0.586314	0.247614	0.060*	
C15	0.6974 (3)	0.4737 (4)	0.2071 (3)	0.0411 (11)	
H15	0.665097	0.484357	0.242605	0.049*	
C16	0.6744 (3)	0.3949 (4)	0.1517 (3)	0.0384 (11)	
C17	0.6542 (3)	0.2269 (4)	0.2958 (3)	0.0376 (11)	
C18	0.6305 (4)	0.1353 (4)	0.2593 (4)	0.0459 (13)	
H18	0.574583	0.126223	0.224728	0.055*	
C19	0.6887 (4)	0.0576 (4)	0.2735 (4)	0.0574 (16)	
H19	0.672835	−0.004031	0.248112	0.069*	
C20	0.7703 (4)	0.0711 (5)	0.3255 (4)	0.0644 (19)	
H20	0.809873	0.018029	0.335597	0.077*	
C21	0.7936 (4)	0.1583 (6)	0.3613 (4)	0.0618 (17)	
H21	0.849179	0.166675	0.396798	0.074*	
C22	0.7358 (3)	0.2363 (5)	0.3460 (3)	0.0488 (14)	
H22	0.753161	0.297922	0.371014	0.059*	
C23	0.5332 (3)	0.3070 (4)	0.3332 (3)	0.0341 (10)	
C24	0.4933 (3)	0.2239 (4)	0.3446 (3)	0.0383 (11)	
H24	0.506052	0.163620	0.323478	0.046*	
C25	0.4326 (3)	0.2237 (4)	0.3873 (3)	0.0403 (12)	
C26	0.3881 (4)	0.1378 (5)	0.3977 (3)	0.0494 (14)	
H26	0.400808	0.075482	0.379555	0.059*	
C27	0.3263 (4)	0.1467 (6)	0.4344 (4)	0.0595 (17)	
C28	0.3074 (4)	0.2364 (6)	0.4640 (3)	0.0587 (17)	
H28	0.264857	0.239537	0.490240	0.070*	
C29	0.3505 (3)	0.3197 (5)	0.4551 (3)	0.0513 (14)	
H29	0.337974	0.380839	0.475108	0.062*	
C30	0.4137 (3)	0.3149 (4)	0.4161 (3)	0.0415 (12)	
C31	0.5119 (3)	0.3938 (4)	0.3702 (3)	0.0369 (11)	
C32	0.5344 (5)	0.5659 (4)	0.3965 (4)	0.0616 (17)	
H32A	0.475980	0.584984	0.367499	0.092*	
H32B	0.573882	0.618559	0.392727	0.092*	
H32C	0.538402	0.554867	0.451767	0.092*	
C33	0.5672 (3)	0.7063 (4)	0.1903 (3)	0.0399 (11)	
C34	0.6399 (3)	0.7506 (4)	0.2525 (3)	0.0438 (12)	
H34	0.631293	0.755572	0.302730	0.053*	
C35	0.7151 (3)	0.7844 (4)	0.2489 (3)	0.0419 (12)	
H35	0.750474	0.811123	0.296725	0.050*	
C36	0.7533 (3)	0.7875 (4)	0.1829 (3)	0.0423 (12)	
O7	0.4370 (6)	0.0263 (8)	0.0209 (6)	0.075 (3)	0.5
C37	0.5084 (18)	−0.034 (3)	0.0604 (14)	0.100 (10)	0.5
H37A	0.552048	0.006461	0.097766	0.120*	0.5
H37B	0.489509	−0.086055	0.090748	0.120*	0.5
C38	0.5450 (14)	−0.080 (2)	0.0055 (13)	0.128 (8)	0.5
H38A	0.537402	−0.152082	0.004958	0.154*	0.5
H38B	0.606666	−0.064579	0.018760	0.154*	0.5
C39	0.4956 (13)	−0.034 (2)	−0.0747 (14)	0.073 (6)	0.5
H39A	0.525268	0.023994	−0.087447	0.087*	0.5
H39B	0.485523	−0.083061	−0.117722	0.087*	0.5
C40	0.4133 (8)	−0.0059 (9)	−0.0577 (8)	0.059 (3)	0.5
H40A	0.383893	0.047510	−0.093328	0.071*	0.5
H40B	0.374534	−0.063351	−0.064957	0.071*	0.5
O8	1.0606 (9)	0.3358 (13)	0.4220 (9)	0.126 (5)	0.587 (16)
C41	0.9722 (12)	0.3162 (15)	0.3956 (11)	0.094 (5)	0.587 (16)
H41A	0.944248	0.347343	0.431763	0.113*	0.587 (16)
H41B	0.962618	0.244206	0.396135	0.113*	0.587 (16)
C42	0.9349 (11)	0.3530 (12)	0.3185 (9)	0.082 (4)	0.587 (16)
H42A	0.881218	0.388515	0.315266	0.099*	0.587 (16)
H42B	0.921747	0.298130	0.280234	0.099*	0.587 (16)
C43	0.9983 (11)	0.4216 (12)	0.3012 (10)	0.078 (4)	0.587 (16)
H43A	0.971975	0.486075	0.282200	0.093*	0.587 (16)
H43B	1.022812	0.393146	0.261618	0.093*	0.587 (16)
C44	1.0658 (12)	0.4317 (14)	0.3806 (11)	0.090 (5)	0.587 (16)
H44A	1.123155	0.440687	0.374167	0.108*	0.587 (16)
H44B	1.053089	0.488380	0.410245	0.108*	0.587 (16)
O8B	0.9965 (13)	0.4595 (16)	0.3290 (13)	0.108 (6)	0.413 (16)
C41B	1.0619 (16)	0.4729 (15)	0.4031 (15)	0.082 (6)	0.413 (16)
H41C	1.108737	0.513132	0.394742	0.098*	0.413 (16)
H41D	1.037590	0.509340	0.439597	0.098*	0.413 (16)
C42B	1.0957 (17)	0.3804 (15)	0.4381 (15)	0.094 (6)	0.413 (16)
H42C	1.108883	0.382041	0.495929	0.112*	0.413 (16)
H42D	1.147525	0.361285	0.424051	0.112*	0.413 (16)
C43B	1.018 (2)	0.3092 (17)	0.3993 (18)	0.098 (6)	0.413 (16)
H43C	1.035681	0.256696	0.368663	0.117*	0.413 (16)
H43D	0.996716	0.278165	0.439884	0.117*	0.413 (16)
C44B	0.9520 (17)	0.374 (2)	0.347 (2)	0.115 (6)	0.413 (16)
H44C	0.921533	0.338300	0.298283	0.138*	0.413 (16)
H44D	0.909914	0.394339	0.373839	0.138*	0.413 (16)

### [4-(6-bromo-2-methoxyquinolin-3-yl)-3-hydroxy-3-(naphthalen-1-yl)-4-phenylbutyl]dimethylazanium 3-carboxyprop-2-enoate tetrahydrofuran sesquihydrate (THF) Atomic displacement parameters (Å^2^)

**Table:**

	*U*^11^	*U*^22^	*U*^33^	*U*^12^	*U*^13^	*U*^23^
Br1	0.0831 (6)	0.1175 (7)	0.0827 (6)	−0.0585 (5)	0.0450 (4)	−0.0131 (5)
O1	0.0297 (17)	0.0386 (18)	0.049 (2)	−0.0031 (14)	0.0107 (16)	−0.0029 (15)
O2	0.0391 (19)	0.0431 (19)	0.049 (2)	−0.0031 (16)	0.0167 (17)	−0.0042 (16)
O3	0.040 (2)	0.047 (2)	0.060 (3)	−0.0115 (17)	0.0223 (19)	−0.0055 (18)
O4	0.042 (2)	0.087 (3)	0.042 (2)	−0.021 (2)	0.0153 (18)	−0.012 (2)
O5	0.045 (2)	0.088 (3)	0.045 (2)	−0.017 (2)	0.0200 (19)	−0.006 (2)
O6	0.033 (2)	0.058 (2)	0.075 (3)	−0.0104 (17)	0.0207 (19)	0.003 (2)
N1	0.0256 (18)	0.035 (2)	0.045 (2)	0.0037 (15)	0.0094 (17)	0.0040 (17)
N2	0.026 (2)	0.054 (2)	0.038 (2)	−0.0008 (18)	0.0093 (18)	−0.0001 (19)
C1	0.024 (2)	0.041 (3)	0.040 (3)	0.0020 (19)	0.011 (2)	0.000 (2)
C2	0.022 (2)	0.035 (2)	0.047 (3)	−0.0028 (18)	0.010 (2)	−0.002 (2)
C3	0.026 (2)	0.039 (2)	0.039 (3)	0.0022 (19)	0.011 (2)	0.002 (2)
C4	0.031 (2)	0.038 (2)	0.042 (3)	0.006 (2)	0.008 (2)	0.000 (2)
C5	0.030 (2)	0.055 (3)	0.074 (4)	0.005 (2)	0.021 (3)	0.011 (3)
C6	0.039 (3)	0.048 (3)	0.045 (3)	0.003 (2)	0.007 (2)	0.010 (2)
C7	0.030 (2)	0.041 (3)	0.037 (3)	0.005 (2)	0.011 (2)	0.001 (2)
C8	0.043 (3)	0.045 (3)	0.039 (3)	0.008 (2)	0.012 (2)	−0.003 (2)
C9	0.057 (4)	0.060 (4)	0.040 (3)	0.025 (3)	0.018 (3)	0.001 (3)
C10	0.048 (3)	0.070 (4)	0.047 (3)	0.023 (3)	0.026 (3)	0.013 (3)
C11	0.035 (3)	0.062 (3)	0.046 (3)	0.016 (2)	0.022 (2)	0.009 (3)
C12	0.040 (3)	0.081 (4)	0.063 (4)	0.014 (3)	0.032 (3)	0.018 (3)
C13	0.035 (3)	0.074 (4)	0.074 (4)	−0.004 (3)	0.021 (3)	0.014 (4)
C14	0.030 (2)	0.055 (3)	0.061 (3)	−0.001 (3)	0.009 (2)	0.009 (3)
C15	0.035 (3)	0.046 (3)	0.043 (3)	0.003 (2)	0.014 (2)	0.002 (2)
C16	0.029 (2)	0.047 (3)	0.041 (3)	0.007 (2)	0.014 (2)	0.005 (2)
C17	0.031 (2)	0.043 (3)	0.042 (3)	0.004 (2)	0.016 (2)	0.006 (2)
C18	0.040 (3)	0.041 (3)	0.057 (3)	0.006 (2)	0.016 (3)	0.002 (2)
C19	0.061 (4)	0.044 (3)	0.076 (4)	0.014 (3)	0.034 (3)	0.011 (3)
C20	0.061 (4)	0.070 (4)	0.069 (4)	0.034 (3)	0.030 (3)	0.016 (3)
C21	0.038 (3)	0.086 (5)	0.060 (4)	0.022 (3)	0.013 (3)	0.010 (4)
C22	0.031 (3)	0.066 (4)	0.049 (3)	0.008 (2)	0.012 (2)	0.006 (3)
C23	0.026 (2)	0.043 (3)	0.035 (2)	0.0018 (19)	0.0109 (19)	0.004 (2)
C24	0.027 (2)	0.048 (3)	0.042 (3)	−0.003 (2)	0.013 (2)	−0.002 (2)
C25	0.029 (2)	0.060 (3)	0.033 (3)	−0.007 (2)	0.012 (2)	0.000 (2)
C26	0.039 (3)	0.065 (4)	0.046 (3)	−0.014 (3)	0.015 (3)	−0.001 (3)
C27	0.039 (3)	0.092 (5)	0.048 (3)	−0.027 (3)	0.014 (3)	−0.004 (3)
C28	0.038 (3)	0.102 (5)	0.040 (3)	−0.018 (3)	0.017 (3)	−0.006 (3)
C29	0.035 (3)	0.084 (4)	0.037 (3)	−0.010 (3)	0.013 (2)	−0.006 (3)
C30	0.026 (2)	0.064 (3)	0.033 (2)	−0.002 (2)	0.0067 (19)	0.001 (2)
C31	0.027 (2)	0.047 (3)	0.036 (3)	−0.001 (2)	0.007 (2)	0.001 (2)
C32	0.073 (4)	0.045 (3)	0.078 (5)	−0.004 (3)	0.041 (4)	−0.010 (3)
C33	0.031 (2)	0.041 (3)	0.048 (3)	−0.002 (2)	0.013 (2)	−0.004 (2)
C34	0.040 (3)	0.048 (3)	0.045 (3)	0.000 (2)	0.014 (2)	−0.004 (2)
C35	0.035 (3)	0.044 (3)	0.044 (3)	−0.002 (2)	0.008 (2)	−0.001 (2)
C36	0.032 (3)	0.044 (3)	0.051 (3)	−0.004 (2)	0.013 (2)	0.002 (2)
O7	0.081 (6)	0.071 (6)	0.084 (7)	0.018 (6)	0.039 (5)	0.017 (6)
C37	0.12 (2)	0.114 (17)	0.061 (12)	0.050 (15)	0.021 (12)	0.013 (12)
C38	0.110 (14)	0.167 (18)	0.125 (15)	0.086 (13)	0.060 (13)	0.063 (14)
C39	0.062 (11)	0.088 (13)	0.085 (13)	0.028 (10)	0.047 (10)	0.018 (11)
C40	0.054 (7)	0.049 (7)	0.081 (9)	−0.004 (6)	0.031 (7)	0.001 (6)
O8	0.104 (9)	0.139 (10)	0.121 (9)	−0.043 (8)	0.014 (7)	0.011 (8)
C41	0.075 (9)	0.096 (9)	0.100 (9)	−0.039 (9)	0.010 (9)	−0.002 (8)
C42	0.074 (8)	0.085 (8)	0.092 (9)	−0.027 (6)	0.032 (7)	−0.040 (7)
C43	0.060 (7)	0.074 (8)	0.095 (10)	0.004 (7)	0.018 (7)	−0.018 (7)
C44	0.069 (7)	0.092 (10)	0.099 (10)	−0.014 (8)	0.009 (7)	0.021 (8)
O8B	0.078 (8)	0.121 (11)	0.115 (11)	−0.026 (9)	0.014 (9)	0.008 (9)
C41B	0.062 (9)	0.064 (10)	0.101 (12)	−0.013 (9)	−0.002 (9)	0.010 (9)
C42B	0.102 (12)	0.065 (10)	0.105 (12)	−0.014 (10)	0.017 (11)	−0.011 (10)
C43B	0.094 (12)	0.075 (9)	0.116 (11)	−0.048 (10)	0.019 (11)	−0.007 (9)
C44B	0.092 (10)	0.105 (11)	0.128 (12)	−0.032 (10)	0.000 (10)	−0.007 (10)

### [4-(6-bromo-2-methoxyquinolin-3-yl)-3-hydroxy-3-(naphthalen-1-yl)-4-phenylbutyl]dimethylazanium 3-carboxyprop-2-enoate tetrahydrofuran sesquihydrate (THF) Geometric parameters (Å, º)

**Table:**

Br1—C27	1.903 (6)	C22—H22	0.9500
O1—C2	1.427 (5)	C23—C24	1.349 (7)
O1—H1O	0.8400	C23—C31	1.443 (7)
O2—C31	1.367 (6)	C24—C25	1.422 (7)
O2—C32	1.437 (7)	C24—H24	0.9500
O3—C33	1.234 (6)	C25—C30	1.409 (8)
O4—C33	1.275 (7)	C25—C26	1.418 (8)
O4—H5	1.15 (8)	C26—C27	1.365 (9)
O5—C36	1.301 (6)	C26—H26	0.9500
O5—H5	1.28 (8)	C27—C28	1.399 (10)
O6—C36	1.217 (6)	C28—C29	1.367 (9)
N1—C6	1.482 (7)	C28—H28	0.9500
N1—C5	1.490 (6)	C29—C30	1.411 (8)
N1—C4	1.493 (6)	C29—H29	0.9500
N1—H1	1.0000	C32—H32A	0.9800
N2—C31	1.305 (6)	C32—H32B	0.9800
N2—C30	1.378 (7)	C32—H32C	0.9800
C1—C17	1.516 (7)	C33—C34	1.493 (7)
C1—C23	1.521 (7)	C34—C35	1.337 (8)
C1—C2	1.566 (7)	C34—H34	0.9500
C1—H1A	1.0000	C35—C36	1.490 (8)
C2—C7	1.545 (7)	C35—H35	0.9500
C2—C3	1.546 (6)	O7—C40	1.408 (15)
C3—C4	1.511 (7)	O7—C37	1.43 (2)
C3—H3A	0.9900	C37—C38	1.43 (2)
C3—H3B	0.9900	C37—H37A	0.9900
C4—H4A	0.9900	C37—H37B	0.9900
C4—H4B	0.9900	C38—C39	1.55 (2)
C5—H5A	0.9800	C38—H38A	0.9900
C5—H5B	0.9800	C38—H38B	0.9900
C5—H5C	0.9800	C39—C40	1.52 (2)
C6—H6A	0.9800	C39—H39A	0.9900
C6—H6B	0.9800	C39—H39B	0.9900
C6—H6C	0.9800	C40—H40A	0.9900
C7—C8	1.368 (7)	C40—H40B	0.9900
C7—C16	1.444 (7)	O8—C41	1.411 (17)
C8—C9	1.408 (8)	O8—C44	1.510 (18)
C8—H8	0.9500	C41—C42	1.42 (2)
C9—C10	1.359 (9)	C41—H41A	0.9900
C9—H9	0.9500	C41—H41B	0.9900
C10—C11	1.400 (9)	C42—C43	1.494 (19)
C10—H10	0.9500	C42—H42A	0.9900
C11—C12	1.423 (9)	C42—H42B	0.9900
C11—C16	1.435 (7)	C43—C44	1.523 (19)
C12—C13	1.351 (10)	C43—H43A	0.9900
C12—H12	0.9500	C43—H43B	0.9900
C13—C14	1.385 (8)	C44—H44A	0.9900
C13—H13	0.9500	C44—H44B	0.9900
C14—C15	1.375 (8)	O8B—C41B	1.45 (2)
C14—H14	0.9500	O8B—C44B	1.46 (2)
C15—C16	1.428 (8)	C41B—C42B	1.44 (2)
C15—H15	0.9500	C41B—H41C	0.9900
C17—C22	1.378 (7)	C41B—H41D	0.9900
C17—C18	1.402 (8)	C42B—C43B	1.58 (2)
C18—C19	1.393 (8)	C42B—H42C	0.9900
C18—H18	0.9500	C42B—H42D	0.9900
C19—C20	1.396 (9)	C43B—C44B	1.49 (2)
C19—H19	0.9500	C43B—H43C	0.9900
C20—C21	1.345 (10)	C43B—H43D	0.9900
C20—H20	0.9500	C44B—H44C	0.9900
C21—C22	1.393 (8)	C44B—H44D	0.9900
C21—H21	0.9500		
			
C2—O1—H1O	109.5	C26—C27—Br1	118.4 (6)
C31—O2—C32	116.8 (4)	C28—C27—Br1	119.0 (5)
C33—O4—H5	106 (4)	C29—C28—C27	119.6 (6)
C36—O5—H5	106 (3)	C29—C28—H28	120.2
C6—N1—C5	110.5 (4)	C27—C28—H28	120.2
C6—N1—C4	111.8 (4)	C28—C29—C30	120.1 (6)
C5—N1—C4	113.2 (4)	C28—C29—H29	119.9
C6—N1—H1	107.0	C30—C29—H29	119.9
C5—N1—H1	107.0	N2—C30—C25	122.2 (5)
C4—N1—H1	107.0	N2—C30—C29	118.2 (5)
C31—N2—C30	116.4 (5)	C25—C30—C29	119.6 (5)
C17—C1—C23	112.3 (4)	N2—C31—O2	117.9 (5)
C17—C1—C2	114.7 (4)	N2—C31—C23	127.0 (5)
C23—C1—C2	110.6 (4)	O2—C31—C23	115.0 (4)
C17—C1—H1A	106.2	O2—C32—H32A	109.5
C23—C1—H1A	106.2	O2—C32—H32B	109.5
C2—C1—H1A	106.2	H32A—C32—H32B	109.5
O1—C2—C7	106.3 (4)	O2—C32—H32C	109.5
O1—C2—C3	107.9 (4)	H32A—C32—H32C	109.5
C7—C2—C3	112.1 (4)	H32B—C32—H32C	109.5
O1—C2—C1	109.5 (4)	O3—C33—O4	122.7 (5)
C7—C2—C1	110.6 (4)	O3—C33—C34	117.4 (5)
C3—C2—C1	110.2 (4)	O4—C33—C34	119.9 (5)
C4—C3—C2	114.4 (4)	C35—C34—C33	130.3 (5)
C4—C3—H3A	108.7	C35—C34—H34	114.8
C2—C3—H3A	108.7	C33—C34—H34	114.8
C4—C3—H3B	108.7	C34—C35—C36	131.3 (5)
C2—C3—H3B	108.7	C34—C35—H35	114.4
H3A—C3—H3B	107.6	C36—C35—H35	114.4
N1—C4—C3	111.7 (4)	O6—C36—O5	122.8 (5)
N1—C4—H4A	109.3	O6—C36—C35	118.1 (5)
C3—C4—H4A	109.3	O5—C36—C35	119.1 (4)
N1—C4—H4B	109.3	C40—O7—C37	104.8 (14)
C3—C4—H4B	109.3	C38—C37—O7	110.9 (16)
H4A—C4—H4B	107.9	C38—C37—H37A	109.5
N1—C5—H5A	109.5	O7—C37—H37A	109.5
N1—C5—H5B	109.5	C38—C37—H37B	109.5
H5A—C5—H5B	109.5	O7—C37—H37B	109.5
N1—C5—H5C	109.5	H37A—C37—H37B	108.0
H5A—C5—H5C	109.5	C37—C38—C39	104.5 (13)
H5B—C5—H5C	109.5	C37—C38—H38A	110.8
N1—C6—H6A	109.5	C39—C38—H38A	110.8
N1—C6—H6B	109.5	C37—C38—H38B	110.8
H6A—C6—H6B	109.5	C39—C38—H38B	110.8
N1—C6—H6C	109.5	H38A—C38—H38B	108.9
H6A—C6—H6C	109.5	C40—C39—C38	99.7 (15)
H6B—C6—H6C	109.5	C40—C39—H39A	111.8
C8—C7—C16	117.5 (5)	C38—C39—H39A	111.8
C8—C7—C2	119.1 (5)	C40—C39—H39B	111.8
C16—C7—C2	123.5 (4)	C38—C39—H39B	111.8
C7—C8—C9	123.4 (6)	H39A—C39—H39B	109.6
C7—C8—H8	118.3	O7—C40—C39	106.2 (13)
C9—C8—H8	118.3	O7—C40—H40A	110.5
C10—C9—C8	119.9 (6)	C39—C40—H40A	110.5
C10—C9—H9	120.0	O7—C40—H40B	110.5
C8—C9—H9	120.0	C39—C40—H40B	110.5
C9—C10—C11	120.1 (5)	H40A—C40—H40B	108.7
C9—C10—H10	120.0	C41—O8—C44	101.6 (13)
C11—C10—H10	120.0	O8—C41—C42	111.1 (14)
C10—C11—C12	120.3 (5)	O8—C41—H41A	109.4
C10—C11—C16	120.5 (5)	C42—C41—H41A	109.4
C12—C11—C16	119.1 (5)	O8—C41—H41B	109.4
C13—C12—C11	122.5 (6)	C42—C41—H41B	109.4
C13—C12—H12	118.7	H41A—C41—H41B	108.0
C11—C12—H12	118.7	C41—C42—C43	107.0 (12)
C12—C13—C14	118.8 (6)	C41—C42—H42A	110.3
C12—C13—H13	120.6	C43—C42—H42A	110.3
C14—C13—H13	120.6	C41—C42—H42B	110.3
C15—C14—C13	121.8 (6)	C43—C42—H42B	110.3
C15—C14—H14	119.1	H42A—C42—H42B	108.6
C13—C14—H14	119.1	C42—C43—C44	102.8 (13)
C14—C15—C16	121.3 (5)	C42—C43—H43A	111.2
C14—C15—H15	119.3	C44—C43—H43A	111.2
C16—C15—H15	119.3	C42—C43—H43B	111.2
C15—C16—C11	116.4 (5)	C44—C43—H43B	111.2
C15—C16—C7	125.0 (5)	H43A—C43—H43B	109.1
C11—C16—C7	118.6 (5)	O8—C44—C43	104.2 (13)
C22—C17—C18	117.7 (5)	O8—C44—H44A	110.9
C22—C17—C1	119.4 (5)	C43—C44—H44A	110.9
C18—C17—C1	122.8 (5)	O8—C44—H44B	110.9
C19—C18—C17	120.4 (5)	C43—C44—H44B	110.9
C19—C18—H18	119.8	H44A—C44—H44B	108.9
C17—C18—H18	119.8	C41B—O8B—C44B	100.5 (16)
C18—C19—C20	119.5 (6)	C42B—C41B—O8B	111.9 (16)
C18—C19—H19	120.3	C42B—C41B—H41C	109.2
C20—C19—H19	120.3	O8B—C41B—H41C	109.2
C21—C20—C19	120.8 (6)	C42B—C41B—H41D	109.2
C21—C20—H20	119.6	O8B—C41B—H41D	109.2
C19—C20—H20	119.6	H41C—C41B—H41D	107.9
C20—C21—C22	119.6 (6)	C41B—C42B—C43B	100.8 (16)
C20—C21—H21	120.2	C41B—C42B—H42C	111.6
C22—C21—H21	120.2	C43B—C42B—H42C	111.6
C17—C22—C21	122.0 (6)	C41B—C42B—H42D	111.6
C17—C22—H22	119.0	C43B—C42B—H42D	111.6
C21—C22—H22	119.0	H42C—C42B—H42D	109.4
C24—C23—C31	114.7 (4)	C44B—C43B—C42B	104.9 (17)
C24—C23—C1	124.8 (5)	C44B—C43B—H43C	110.8
C31—C23—C1	120.5 (4)	C42B—C43B—H43C	110.8
C23—C24—C25	122.1 (5)	C44B—C43B—H43D	110.8
C23—C24—H24	119.0	C42B—C43B—H43D	110.8
C25—C24—H24	119.0	H43C—C43B—H43D	108.8
C30—C25—C26	119.7 (5)	O8B—C44B—C43B	106.7 (18)
C30—C25—C24	117.4 (5)	O8B—C44B—H44C	110.4
C26—C25—C24	122.8 (5)	C43B—C44B—H44C	110.4
C27—C26—C25	118.4 (6)	O8B—C44B—H44D	110.4
C27—C26—H26	120.8	C43B—C44B—H44D	110.4
C25—C26—H26	120.8	H44C—C44B—H44D	108.6
C26—C27—C28	122.6 (6)		
			
C17—C1—C2—O1	71.7 (5)	C17—C1—C23—C24	−42.3 (6)
C23—C1—C2—O1	−56.5 (5)	C2—C1—C23—C24	87.2 (6)
C17—C1—C2—C7	−45.2 (5)	C17—C1—C23—C31	140.2 (5)
C23—C1—C2—C7	−173.4 (4)	C2—C1—C23—C31	−90.3 (5)
C17—C1—C2—C3	−169.8 (4)	C31—C23—C24—C25	2.3 (7)
C23—C1—C2—C3	62.0 (5)	C1—C23—C24—C25	−175.3 (4)
O1—C2—C3—C4	−66.4 (5)	C23—C24—C25—C30	1.6 (7)
C7—C2—C3—C4	50.4 (5)	C23—C24—C25—C26	177.9 (5)
C1—C2—C3—C4	174.1 (4)	C30—C25—C26—C27	1.3 (8)
C6—N1—C4—C3	163.5 (4)	C24—C25—C26—C27	−175.0 (5)
C5—N1—C4—C3	−70.9 (5)	C25—C26—C27—C28	−2.0 (9)
C2—C3—C4—N1	174.3 (4)	C25—C26—C27—Br1	175.3 (4)
O1—C2—C7—C8	−1.2 (6)	C26—C27—C28—C29	1.4 (10)
C3—C2—C7—C8	−118.9 (5)	Br1—C27—C28—C29	−175.9 (4)
C1—C2—C7—C8	117.6 (5)	C27—C28—C29—C30	0.0 (9)
O1—C2—C7—C16	179.0 (4)	C31—N2—C30—C25	0.4 (7)
C3—C2—C7—C16	61.3 (6)	C31—N2—C30—C29	−179.2 (5)
C1—C2—C7—C16	−62.1 (6)	C26—C25—C30—N2	−179.6 (5)
C16—C7—C8—C9	0.9 (8)	C24—C25—C30—N2	−3.2 (7)
C2—C7—C8—C9	−178.9 (5)	C26—C25—C30—C29	0.0 (7)
C7—C8—C9—C10	−1.0 (8)	C24—C25—C30—C29	176.5 (5)
C8—C9—C10—C11	0.7 (8)	C28—C29—C30—N2	179.0 (5)
C9—C10—C11—C12	−178.0 (5)	C28—C29—C30—C25	−0.7 (8)
C9—C10—C11—C16	−0.4 (8)	C30—N2—C31—O2	−177.0 (4)
C10—C11—C12—C13	178.4 (6)	C30—N2—C31—C23	4.2 (7)
C16—C11—C12—C13	0.8 (9)	C32—O2—C31—N2	−1.8 (7)
C11—C12—C13—C14	−0.4 (10)	C32—O2—C31—C23	177.2 (5)
C12—C13—C14—C15	0.0 (9)	C24—C23—C31—N2	−5.6 (7)
C13—C14—C15—C16	−0.1 (8)	C1—C23—C31—N2	172.1 (5)
C14—C15—C16—C11	0.5 (7)	C24—C23—C31—O2	175.6 (4)
C14—C15—C16—C7	−178.1 (5)	C1—C23—C31—O2	−6.7 (6)
C10—C11—C16—C15	−178.5 (5)	O3—C33—C34—C35	177.4 (6)
C12—C11—C16—C15	−0.8 (7)	O4—C33—C34—C35	−1.5 (9)
C10—C11—C16—C7	0.3 (8)	C33—C34—C35—C36	−1.7 (10)
C12—C11—C16—C7	177.9 (5)	C34—C35—C36—O6	−174.5 (6)
C8—C7—C16—C15	178.1 (5)	C34—C35—C36—O5	6.4 (9)
C2—C7—C16—C15	−2.2 (8)	C40—O7—C37—C38	18 (3)
C8—C7—C16—C11	−0.5 (7)	O7—C37—C38—C39	5 (3)
C2—C7—C16—C11	179.2 (5)	C37—C38—C39—C40	−25 (3)
C23—C1—C17—C22	−98.2 (5)	C37—O7—C40—C39	−35 (2)
C2—C1—C17—C22	134.4 (5)	C38—C39—C40—O7	37 (2)
C23—C1—C17—C18	80.6 (6)	C44—O8—C41—C42	32 (2)
C2—C1—C17—C18	−46.7 (7)	O8—C41—C42—C43	−14 (2)
C22—C17—C18—C19	−0.6 (8)	C41—C42—C43—C44	−9.4 (19)
C1—C17—C18—C19	−179.4 (5)	C41—O8—C44—C43	−36 (2)
C17—C18—C19—C20	1.1 (9)	C42—C43—C44—O8	27.9 (18)
C18—C19—C20—C21	−0.6 (10)	C44B—O8B—C41B—C42B	39 (3)
C19—C20—C21—C22	−0.4 (11)	O8B—C41B—C42B—C43B	−25 (3)
C18—C17—C22—C21	−0.4 (9)	C41B—C42B—C43B—C44B	0 (4)
C1—C17—C22—C21	178.5 (5)	C41B—O8B—C44B—C43B	−37 (3)
C20—C21—C22—C17	0.9 (10)	C42B—C43B—C44B—O8B	23 (4)

### [4-(6-bromo-2-methoxyquinolin-3-yl)-3-hydroxy-3-(naphthalen-1-yl)-4-phenylbutyl]dimethylazanium 3-carboxyprop-2-enoate tetrahydrofuran sesquihydrate (THF) Hydrogen-bond geometry (Å, º)

**Table:**

*D*—H···*A*	*D*—H	H···*A*	*D*···*A*	*D*—H···*A*
O1—H1*O*···O6^i^	0.84	1.99	2.824 (5)	176
O4—H5···O5	1.15 (8)	1.28 (8)	2.422 (5)	171 (7)
N1—H1···O3	1.00	1.70	2.699 (5)	175
N1—H1···O4	1.00	2.63	3.310 (6)	125
C3—H3*A*···O6^i^	0.99	2.53	3.258 (6)	130
C3—H3*B*···O3	0.99	2.65	3.314 (6)	125
C6—H6*B*···O4^ii^	0.98	2.55	3.173 (7)	122
C26—H26···O8*B*^i^	0.95	2.57	3.44 (3)	152
C42—H42*B*···O3^iii^	0.99	2.67	3.478 (17)	138

Symmetry codes: (i) *x*−1/2, *y*−1/2, *z*; (ii) −*x*+1, *y*, −*z*; (iii) *x*+1/2, *y*−1/2, *z*.

### [4-(6-bromo-2-methoxyquinolin-3-yl)-3-hydroxy-3-(naphthalen-1-yl)-4-phenylbutyl]dimethylazanium 3-carboxyprop-2-enoate ethyl acetate 0.821-solvate (ethyl_acetate) Crystal data

**Table:**

C_32_H_32_BrN_2_O_2_^+^·C_4_H_3_O_4_^−^·0.821C_4_H_8_O_2_	*F*(000) = 1550
*M**_r_* = 743.88	*D*_x_ = 1.326 Mg m^−^^3^
Monoclinic, *P*2_1_	Mo *K*α radiation, λ = 0.71073 Å
*a* = 16.1525 (10) Å	Cell parameters from 9891 reflections
*b* = 13.5353 (9) Å	θ = 2.5–30.9°
*c* = 17.8572 (11) Å	µ = 1.16 mm^−^^1^
β = 107.359 (2)°	*T* = 150 K
*V* = 3726.3 (4) Å^3^	Block, colourless
*Z* = 4	0.45 × 0.43 × 0.37 mm

### [4-(6-bromo-2-methoxyquinolin-3-yl)-3-hydroxy-3-(naphthalen-1-yl)-4-phenylbutyl]dimethylazanium 3-carboxyprop-2-enoate ethyl acetate 0.821-solvate (ethyl_acetate) Data collection

**Table:**

Bruker AXS D8 Quest with PhotonII CPAD diffractometer	28338 independent reflections
Radiation source: fine focus sealed tube X-ray source	15758 reflections with *I* > 2σ(*I*)
Triumph curved graphite crystal monochromator	*R*_int_ = 0.082
ω and phi scans	θ_max_ = 33.2°, θ_min_ = 2.4°
Absorption correction: multi-scan (SADABS; Krause *et al.*, 2015)	*h* = −22→24
*T*_min_ = 0.670, *T*_max_ = 0.742	*k* = −20→20
153291 measured reflections	*l* = −27→27

### [4-(6-bromo-2-methoxyquinolin-3-yl)-3-hydroxy-3-(naphthalen-1-yl)-4-phenylbutyl]dimethylazanium 3-carboxyprop-2-enoate ethyl acetate 0.821-solvate (ethyl_acetate) Refinement

**Table:**

Refinement on *F*^2^	Secondary atom site location: difference Fourier map
Least-squares matrix: full	Hydrogen site location: mixed
*R*[*F*^2^ > 2σ(*F*^2^)] = 0.062	H atoms treated by a mixture of independent and constrained refinement
*wR*(*F*^2^) = 0.200	*w* = 1/[σ^2^(*F*_o_^2^) + (0.0795*P*)^2^ + 0.9081*P*] where *P* = (*F*_o_^2^ + 2*F*_c_^2^)/3
*S* = 1.02	(Δ/σ)_max_ = 0.003
28338 reflections	Δρ_max_ = 0.88 e Å^−^^3^
1359 parameters	Δρ_min_ = −1.03 e Å^−^^3^
1870 restraints	Absolute structure: Flack *x* determined using 5601 quotients [(*I*^+^)-(*I*^-^)]/[(*I*^+^)+(*I*^-^)] (Parsons *et al.*, 2013).
Primary atom site location: structure-invariant direct methods	Absolute structure parameter: 0.020 (4)

### [4-(6-bromo-2-methoxyquinolin-3-yl)-3-hydroxy-3-(naphthalen-1-yl)-4-phenylbutyl]dimethylazanium 3-carboxyprop-2-enoate ethyl acetate 0.821-solvate (ethyl_acetate) Special details

**Table:**

Geometry. All esds (except the esd in the dihedral angle between two l.s. planes) are estimated using the full covariance matrix. The cell esds are taken into account individually in the estimation of esds in distances, angles and torsion angles; correlations between esds in cell parameters are only used when they are defined by crystal symmetry. An approximate (isotropic) treatment of cell esds is used for estimating esds involving l.s. planes.
Refinement. The structure exhibits pseudo C-centered symmetry emulating space group C2. C2 symmetry was observed for the related hemihydrate and acetone / hexane solvates. Exact translation and two-fold symmetry for the ethyl acetate solvate is broken by the solvate molecules and by a slight modulation of cations and anions. Mean intensity is 1.8 for reflections that should be systematically absent for C2, vs 6.9 for all reflections (2.2 vs 2.9 for mean intensity / sigma). Ethyl acetate molecules are arranged into two clusters with light and severe disorder. Solvate disorder induces disorder of a cation phenyl and a cation naphtyl group. The site associated with the ethyl acetate molecule of O1/O2 was refined as two-fold disordered and as fully occupied. The site associated with the ethyl acetate molecule of O3/O4 was refined as five-fold disordered and only partially occupied. One of the moieties of O3/O4 (suffix F) extends away from the main cluster. It induces the disorder of the O1/O2 ethyl acetate, and for the naphtyl group of cation A. A common occupancy ratio was used for these three entities. Disorder of the phenyl group of cation B is correlated with multiple disordered moieties of the severely disordered ethyl acetate and was refined independently. All ethyl acetate moieties were restrained to have similar geometries. The acetate sections were restrained to be close to planar. The ethyl C-C bond distances were restrained to a target value (1.55 (2) Angstrom). Disordered phenyl and naphtyl groups were restrained to have similar geometries as their not disordered counterparts in the other cation. Uij components of ADPs for disordered atoms closer to each other than 2.0 Angstrom were restrained to be similar. Subject to these conditions the occupancy rates refined to 0.874 (3) to 0.126 (3) for the two-fold disordered ethyl acetate of O1/O2 (shared with the naphtyl disorder of cation A). The occupancy rates for the partially occupied site refined to 0.171 (7), 0.183 (7), 0.126 (3) (same as minor moiety of O1/O2 ethyl acetate), 0.074 (6) and 0.087 (6), for a total occupancy of 0.641. The occupancy ratio of the phenyl disorder of cation B refined to 0.573 (17) to 0.427 (17).

### [4-(6-bromo-2-methoxyquinolin-3-yl)-3-hydroxy-3-(naphthalen-1-yl)-4-phenylbutyl]dimethylazanium 3-carboxyprop-2-enoate ethyl acetate 0.821-solvate (ethyl_acetate) Fractional atomic coordinates and isotropic or equivalent isotropic displacement parameters (Å^2^)

**Table:**

	*x*	*y*	*z*	*U*_iso_*/*U*_eq_	Occ. (<1)
Br1A	0.02899 (4)	−0.1368 (3)	0.47002 (5)	0.0853 (2)	
O1A	0.23412 (18)	0.0834 (4)	0.16595 (15)	0.0384 (6)	
H1AB	0.190 (4)	0.088 (5)	0.182 (3)	0.058*	
O2A	0.31927 (16)	0.2987 (4)	0.36367 (14)	0.0343 (5)	
O3A	0.25764 (18)	0.5025 (4)	0.21465 (17)	0.0430 (6)	
O4A	0.3323 (2)	0.5009 (5)	0.12996 (19)	0.0776 (14)	
O5A	0.4687 (3)	0.5653 (5)	0.1195 (2)	0.0789 (14)	
H5A	0.410 (6)	0.531 (7)	0.128 (5)	0.118*	
O6A	0.58605 (19)	0.6327 (4)	0.19870 (19)	0.0508 (7)	
N1A	0.15387 (17)	0.3824 (4)	0.10491 (16)	0.0290 (5)	
H1A	0.192127	0.429610	0.142926	0.035*	
N2A	0.21055 (18)	0.2293 (4)	0.40474 (16)	0.0309 (6)	
C1A	0.3501 (2)	0.1343 (4)	0.28290 (18)	0.0295 (6)	
H1AA	0.389428	0.191936	0.302046	0.035*	
C2A	0.2981 (2)	0.1569 (4)	0.19441 (19)	0.0305 (6)	
C3A	0.2523 (2)	0.2579 (4)	0.18924 (18)	0.0280 (6)	
H3AA	0.209997	0.254603	0.219490	0.034*	
H3AB	0.295907	0.308814	0.213855	0.034*	
C4A	0.2051 (2)	0.2892 (4)	0.10544 (19)	0.0320 (7)	
H4AA	0.247771	0.300417	0.076437	0.038*	
H4AB	0.165422	0.235709	0.078618	0.038*	
C5A	0.0750 (2)	0.3651 (5)	0.1305 (2)	0.0423 (8)	
H5AA	0.045311	0.428010	0.131294	0.063*	
H5AB	0.092183	0.336078	0.183165	0.063*	
H5AC	0.035812	0.319704	0.093730	0.063*	
C6A	0.1289 (3)	0.4300 (5)	0.0261 (2)	0.0406 (8)	
H6AA	0.181244	0.451969	0.013879	0.061*	
H6AB	0.091537	0.487025	0.026100	0.061*	
H6AC	0.097568	0.382291	−0.013531	0.061*	
C7A	0.3613 (3)	0.1571 (5)	0.1439 (3)	0.0343 (10)	0.874 (3)
C8A	0.3446 (4)	0.0912 (5)	0.0821 (3)	0.0432 (11)	0.874 (3)
H8A	0.295157	0.049649	0.072597	0.052*	0.874 (3)
C9A	0.3980 (4)	0.0828 (6)	0.0324 (3)	0.0508 (13)	0.874 (3)
H9A	0.384385	0.036339	−0.009333	0.061*	0.874 (3)
C10A	0.4694 (4)	0.1420 (6)	0.0449 (4)	0.0604 (14)	0.874 (3)
H10A	0.505210	0.136602	0.011493	0.072*	0.874 (3)
C11A	0.4905 (4)	0.2108 (6)	0.1068 (4)	0.0623 (14)	0.874 (3)
C12A	0.5640 (5)	0.2752 (8)	0.1180 (6)	0.091 (2)	0.874 (3)
H12A	0.599152	0.269222	0.084066	0.109*	0.874 (3)
C13A	0.5843 (5)	0.3433 (8)	0.1748 (6)	0.096 (2)	0.874 (3)
H13A	0.633602	0.384386	0.180943	0.115*	0.874 (3)
C14A	0.5335 (4)	0.3540 (7)	0.2248 (5)	0.0773 (17)	0.874 (3)
H14A	0.548379	0.401996	0.265419	0.093*	0.874 (3)
C15A	0.4603 (4)	0.2942 (6)	0.2155 (4)	0.0519 (12)	0.874 (3)
H15A	0.425838	0.303429	0.249735	0.062*	0.874 (3)
C16A	0.4361 (3)	0.2208 (6)	0.1569 (3)	0.0456 (11)	0.874 (3)
C7C	0.3427 (19)	0.132 (2)	0.1331 (17)	0.041 (4)	0.126 (3)
C8C	0.3206 (19)	0.056 (2)	0.0799 (16)	0.041 (4)	0.126 (3)
H8C	0.273649	0.013988	0.079459	0.049*	0.126 (3)
C9C	0.367 (2)	0.042 (2)	0.0258 (16)	0.042 (4)	0.126 (3)
H9C	0.350668	−0.009539	−0.012149	0.050*	0.126 (3)
C10C	0.437 (2)	0.100 (2)	0.0273 (19)	0.046 (4)	0.126 (3)
H10C	0.467759	0.090004	−0.009734	0.056*	0.126 (3)
C11C	0.463 (2)	0.176 (2)	0.0845 (19)	0.052 (3)	0.126 (3)
C12C	0.533 (2)	0.240 (3)	0.084 (2)	0.068 (4)	0.126 (3)
H12C	0.569295	0.221262	0.052987	0.081*	0.126 (3)
C13C	0.550 (3)	0.325 (3)	0.125 (3)	0.076 (4)	0.126 (3)
H13C	0.587427	0.373898	0.114413	0.091*	0.126 (3)
C14C	0.510 (3)	0.339 (3)	0.183 (3)	0.073 (3)	0.126 (3)
H14C	0.529544	0.390797	0.219989	0.088*	0.126 (3)
C15C	0.442 (3)	0.278 (3)	0.189 (2)	0.060 (4)	0.126 (3)
H15C	0.412938	0.293104	0.226547	0.072*	0.126 (3)
C16C	0.416 (2)	0.195 (3)	0.140 (2)	0.047 (3)	0.126 (3)
C17A	0.4079 (2)	0.0434 (4)	0.2939 (2)	0.0350 (7)	
C18A	0.3816 (3)	−0.0471 (5)	0.2559 (3)	0.0456 (9)	
H18A	0.325410	−0.053527	0.219538	0.055*	
C19A	0.4386 (3)	−0.1282 (5)	0.2718 (3)	0.0564 (11)	
H19A	0.420833	−0.189045	0.245421	0.068*	
C20A	0.5198 (3)	−0.1203 (5)	0.3249 (3)	0.0591 (13)	
H20A	0.557645	−0.175675	0.336163	0.071*	
C21A	0.5452 (3)	−0.0320 (6)	0.3613 (3)	0.0649 (15)	
H21A	0.601430	−0.026192	0.397750	0.078*	
C22A	0.4905 (3)	0.0499 (5)	0.3463 (2)	0.0504 (11)	
H22A	0.509958	0.110631	0.372267	0.060*	
C23A	0.2886 (2)	0.1306 (4)	0.33303 (17)	0.0275 (6)	
C24A	0.2493 (2)	0.0476 (4)	0.34742 (19)	0.0311 (6)	
H24A	0.262493	−0.014151	0.328403	0.037*	
C25A	0.1885 (2)	0.0516 (4)	0.39060 (19)	0.0316 (7)	
C26A	0.1467 (2)	−0.0334 (5)	0.4085 (2)	0.0401 (8)	
H26A	0.161054	−0.097165	0.394024	0.048*	
C27A	0.0858 (3)	−0.0227 (5)	0.4466 (2)	0.0442 (9)	
C28A	0.0625 (2)	0.0697 (5)	0.4696 (2)	0.0437 (9)	
H28A	0.019073	0.074902	0.495367	0.052*	
C29A	0.1037 (2)	0.1529 (5)	0.4540 (2)	0.0381 (8)	
H29A	0.088576	0.215925	0.469269	0.046*	
C30A	0.1683 (2)	0.1456 (4)	0.41547 (18)	0.0307 (7)	
C31A	0.2695 (2)	0.2206 (4)	0.36887 (18)	0.0287 (6)	
C32A	0.3048 (3)	0.3891 (4)	0.4001 (2)	0.0456 (9)	
H32A	0.349358	0.437409	0.398264	0.068*	
H32B	0.307834	0.376197	0.454834	0.068*	
H32C	0.247392	0.415309	0.371967	0.068*	
C33A	0.3246 (3)	0.5217 (5)	0.1966 (2)	0.0427 (9)	
C34A	0.3976 (2)	0.5695 (4)	0.2579 (2)	0.0389 (8)	
H34A	0.388111	0.578612	0.307481	0.047*	
C35A	0.4743 (3)	0.6012 (4)	0.2532 (2)	0.0408 (8)	
H35A	0.510715	0.629067	0.300418	0.049*	
C36A	0.5130 (3)	0.6005 (5)	0.1868 (2)	0.0432 (9)	
Br1B	0.54606 (5)	0.3491 (3)	0.47124 (6)	0.0999 (3)	
O1B	0.74520 (18)	0.5862 (4)	0.17331 (16)	0.0383 (5)	
H1BB	0.694 (4)	0.594 (5)	0.186 (3)	0.057*	
O2B	0.81300 (16)	0.8084 (4)	0.36580 (14)	0.0355 (5)	
O3B	0.7527 (2)	1.0064 (4)	0.21261 (19)	0.0519 (7)	
O4B	0.8241 (2)	1.0126 (5)	0.12531 (18)	0.0597 (9)	
O5B	0.9612 (2)	1.0754 (4)	0.11490 (18)	0.0575 (9)	
H5B	0.901 (5)	1.044 (6)	0.130 (4)	0.086*	
O6B	1.0803 (2)	1.1377 (4)	0.19574 (19)	0.0508 (7)	
N1B	0.65378 (17)	0.8812 (4)	0.10484 (16)	0.0319 (6)	
H1B	0.690381	0.930731	0.141697	0.038*	
N2B	0.70866 (19)	0.7295 (4)	0.40698 (16)	0.0342 (6)	
C1B	0.8576 (2)	0.6475 (4)	0.28897 (19)	0.0314 (7)	
H1BA	0.888029	0.711712	0.306093	0.038*	
C2B	0.8061 (2)	0.6645 (4)	0.1995 (2)	0.0303 (6)	
C3B	0.7553 (2)	0.7625 (4)	0.19154 (18)	0.0275 (6)	
H3BA	0.712441	0.756992	0.220996	0.033*	
H3BB	0.796240	0.816130	0.215834	0.033*	
C4B	0.7083 (2)	0.7905 (4)	0.10731 (19)	0.0322 (7)	
H4BA	0.751121	0.803333	0.078703	0.039*	
H4BB	0.670834	0.735065	0.081039	0.039*	
C5B	0.5752 (2)	0.8621 (5)	0.1307 (3)	0.0468 (9)	
H5BA	0.544387	0.924353	0.131085	0.070*	
H5BB	0.593043	0.833766	0.183616	0.070*	
H5BC	0.536861	0.815679	0.094354	0.070*	
C6B	0.6272 (3)	0.9261 (5)	0.0248 (2)	0.0501 (10)	
H6BA	0.678893	0.947245	0.011197	0.075*	
H6BB	0.589832	0.983341	0.024186	0.075*	
H6BC	0.595340	0.877140	−0.013430	0.075*	
C7B	0.8673 (2)	0.6619 (4)	0.1480 (2)	0.0336 (7)	
C8B	0.8527 (3)	0.5916 (5)	0.0892 (2)	0.0406 (8)	
H8B	0.805953	0.546601	0.082820	0.049*	
C9B	0.9045 (3)	0.5840 (5)	0.0383 (2)	0.0471 (10)	
H9B	0.891313	0.535741	−0.002152	0.057*	
C10B	0.9730 (3)	0.6453 (5)	0.0468 (2)	0.0484 (10)	
H10B	1.007915	0.639193	0.012572	0.058*	
C11B	0.9932 (3)	0.7192 (5)	0.1068 (3)	0.0457 (9)	
C12B	1.0638 (3)	0.7849 (5)	0.1143 (3)	0.0582 (12)	
H12B	1.097921	0.778083	0.079533	0.070*	
C13B	1.0839 (3)	0.8564 (6)	0.1691 (3)	0.0614 (13)	
H13B	1.132210	0.898513	0.173600	0.074*	
C14B	1.0325 (3)	0.8681 (5)	0.2194 (3)	0.0497 (9)	
H14B	1.045339	0.919472	0.257373	0.060*	
C15B	0.9629 (2)	0.8052 (4)	0.2145 (2)	0.0408 (8)	
H15B	0.930025	0.813986	0.250104	0.049*	
C16B	0.9394 (2)	0.7285 (4)	0.1579 (2)	0.0363 (7)	
C17B	0.9295 (9)	0.5716 (10)	0.3094 (10)	0.035 (2)	0.573 (17)
C18B	0.9119 (7)	0.4729 (10)	0.2814 (8)	0.050 (2)	0.573 (17)
H18B	0.855748	0.455349	0.248841	0.060*	0.573 (17)
C19B	0.9782 (8)	0.4019 (9)	0.3025 (8)	0.057 (2)	0.573 (17)
H19B	0.967212	0.336462	0.282785	0.068*	0.573 (17)
C20B	1.0582 (7)	0.4260 (9)	0.3508 (8)	0.048 (2)	0.573 (17)
H20B	1.102266	0.377141	0.365400	0.057*	0.573 (17)
C21B	1.0752 (6)	0.5208 (9)	0.3784 (7)	0.046 (2)	0.573 (17)
H21B	1.130996	0.536881	0.412502	0.055*	0.573 (17)
C22B	1.0118 (7)	0.5936 (9)	0.3570 (7)	0.0351 (19)	0.573 (17)
H22B	1.025205	0.659266	0.375475	0.042*	0.573 (17)
C17D	0.9151 (10)	0.5534 (13)	0.2970 (11)	0.030 (3)	0.427 (17)
C18D	0.8899 (10)	0.4626 (11)	0.2593 (9)	0.038 (2)	0.427 (17)
H18D	0.833858	0.453834	0.223011	0.045*	0.427 (17)
C19D	0.9505 (10)	0.3852 (11)	0.2772 (9)	0.047 (2)	0.427 (17)
H19D	0.933942	0.322169	0.254171	0.057*	0.427 (17)
C20D	1.0328 (11)	0.3976 (12)	0.3271 (8)	0.047 (3)	0.427 (17)
H20D	1.071394	0.342941	0.339189	0.056*	0.427 (17)
C21D	1.0601 (10)	0.4885 (13)	0.3596 (9)	0.049 (3)	0.427 (17)
H21D	1.118237	0.499151	0.390961	0.059*	0.427 (17)
C22D	0.9989 (10)	0.5646 (12)	0.3448 (10)	0.040 (3)	0.427 (17)
H22D	1.015872	0.626967	0.368802	0.048*	0.427 (17)
C23B	0.7955 (2)	0.6383 (4)	0.33776 (19)	0.0316 (7)	
C24B	0.7586 (2)	0.5520 (4)	0.3511 (2)	0.0359 (7)	
H24B	0.774592	0.491703	0.331895	0.043*	
C25B	0.6964 (2)	0.5511 (5)	0.3935 (2)	0.0386 (8)	
C26B	0.6586 (3)	0.4623 (5)	0.4101 (3)	0.0504 (10)	
H26B	0.676312	0.399991	0.395642	0.060*	
C27B	0.5956 (3)	0.4685 (5)	0.4476 (3)	0.0554 (11)	
C28B	0.5681 (3)	0.5580 (5)	0.4700 (2)	0.0495 (10)	
H28B	0.523840	0.559434	0.495026	0.059*	
C29B	0.6057 (2)	0.6449 (5)	0.4557 (2)	0.0419 (9)	
H29B	0.587413	0.706191	0.471219	0.050*	
C30B	0.6717 (2)	0.6430 (5)	0.41775 (18)	0.0350 (7)	
C31B	0.7691 (2)	0.7254 (4)	0.37173 (18)	0.0306 (6)	
C32B	0.7903 (3)	0.8970 (5)	0.3988 (3)	0.0513 (10)	
H32D	0.831311	0.949565	0.396650	0.077*	
H32E	0.792681	0.885069	0.453525	0.077*	
H32F	0.731492	0.917139	0.368821	0.077*	
C33B	0.8175 (3)	1.0299 (4)	0.1929 (2)	0.0432 (9)	
C34B	0.8909 (3)	1.0788 (4)	0.2532 (2)	0.0437 (9)	
H34B	0.881485	1.088332	0.302744	0.052*	
C35B	0.9672 (3)	1.1112 (4)	0.2490 (2)	0.0412 (8)	
H35B	1.002838	1.140866	0.295786	0.049*	
C36B	1.0061 (3)	1.1087 (4)	0.1827 (2)	0.0413 (8)	
O1	0.6549 (4)	0.1187 (5)	0.5572 (4)	0.118 (2)	0.874 (3)
O2	0.7608 (4)	0.1996 (6)	0.5280 (4)	0.0972 (16)	0.874 (3)
C1	0.7171 (5)	0.1724 (6)	0.5806 (5)	0.088 (2)	0.874 (3)
C2	0.7566 (7)	0.2127 (8)	0.6606 (5)	0.119 (3)	0.874 (3)
H2A	0.758100	0.161079	0.699532	0.178*	0.874 (3)
H2B	0.722034	0.268592	0.669280	0.178*	0.874 (3)
H2C	0.815851	0.234903	0.665979	0.178*	0.874 (3)
C3	0.7233 (6)	0.1654 (8)	0.4500 (5)	0.110 (3)	0.874 (3)
H3A	0.666865	0.198517	0.426215	0.131*	0.874 (3)
H3B	0.713123	0.093288	0.450041	0.131*	0.874 (3)
C4	0.7838 (7)	0.1883 (10)	0.4041 (6)	0.129 (4)	0.874 (3)
H4A	0.841661	0.162956	0.431855	0.155*	0.874 (3)
H4B	0.786716	0.259958	0.397655	0.155*	0.874 (3)
H4C	0.762979	0.156936	0.352343	0.155*	0.874 (3)
O1E	0.8165 (19)	0.1948 (18)	0.5815 (15)	0.109 (8)	0.126 (3)
O2E	0.7400 (17)	0.0863 (19)	0.4952 (10)	0.112 (5)	0.126 (3)
C1E	0.7688 (14)	0.1239 (16)	0.5705 (9)	0.107 (5)	0.126 (3)
C2E	0.729 (2)	0.075 (3)	0.6260 (13)	0.124 (9)	0.126 (3)
H2EA	0.693651	0.019149	0.599679	0.186*	0.126 (3)
H2EB	0.775379	0.050678	0.671552	0.186*	0.126 (3)
H2EC	0.693092	0.122312	0.643309	0.186*	0.126 (3)
C3E	0.7575 (15)	0.147 (3)	0.4366 (10)	0.106 (5)	0.126 (3)
H3EA	0.749241	0.217106	0.447395	0.127*	0.126 (3)
H3EB	0.717256	0.129871	0.384435	0.127*	0.126 (3)
C4E	0.8485 (17)	0.129 (4)	0.437 (2)	0.108 (11)	0.126 (3)
H4EA	0.861717	0.170158	0.397286	0.129*	0.126 (3)
H4EB	0.887824	0.146197	0.489146	0.129*	0.126 (3)
H4EC	0.855948	0.059258	0.426400	0.129*	0.126 (3)
O3	0.8705 (10)	0.2818 (19)	0.0451 (13)	0.081 (6)	0.171 (7)
O4	0.7393 (9)	0.2293 (13)	0.0417 (9)	0.072 (4)	0.171 (7)
C5	0.8128 (9)	0.2850 (15)	0.0752 (10)	0.075 (4)	0.171 (7)
C6	0.8104 (16)	0.338 (2)	0.1475 (12)	0.077 (6)	0.171 (7)
H6A	0.754381	0.326023	0.156728	0.115*	0.171 (7)
H6B	0.857174	0.313172	0.192275	0.115*	0.171 (7)
H6C	0.818105	0.408710	0.141113	0.115*	0.171 (7)
C7	0.7350 (15)	0.1865 (12)	−0.0320 (10)	0.075 (5)	0.171 (7)
H7A	0.693641	0.130522	−0.043205	0.090*	0.171 (7)
H7B	0.792785	0.161219	−0.031220	0.090*	0.171 (7)
C8	0.706 (3)	0.264 (2)	−0.0934 (11)	0.077 (6)	0.171 (7)
H8D	0.702569	0.235315	−0.144721	0.116*	0.171 (7)
H8E	0.648570	0.287848	−0.093929	0.116*	0.171 (7)
H8F	0.747379	0.318442	−0.081984	0.116*	0.171 (7)
O3E	0.8603 (8)	0.3188 (19)	0.0493 (12)	0.081 (5)	0.183 (7)
O4E	0.7167 (8)	0.3265 (17)	0.0171 (8)	0.077 (4)	0.183 (7)
C5E	0.7968 (8)	0.3108 (17)	0.0715 (8)	0.075 (4)	0.183 (7)
C6E	0.7921 (15)	0.294 (2)	0.1523 (8)	0.078 (6)	0.183 (7)
H6EA	0.731200	0.291174	0.151394	0.118*	0.183 (7)
H6EB	0.821006	0.232008	0.172636	0.118*	0.183 (7)
H6EC	0.820974	0.348891	0.186134	0.118*	0.183 (7)
C7E	0.7173 (13)	0.3305 (12)	−0.0622 (8)	0.072 (4)	0.183 (7)
H7EA	0.771446	0.362205	−0.065366	0.087*	0.183 (7)
H7EB	0.667511	0.369919	−0.093834	0.087*	0.183 (7)
C8E	0.712 (3)	0.2280 (15)	−0.0930 (14)	0.072 (6)	0.183 (7)
H8EA	0.712015	0.229364	−0.147725	0.108*	0.183 (7)
H8EB	0.761364	0.189668	−0.061530	0.108*	0.183 (7)
H8EC	0.657780	0.197356	−0.089902	0.108*	0.183 (7)
O3F	0.7477 (16)	0.2910 (16)	0.2656 (11)	0.091 (10)	0.126 (3)
O4F	0.7156 (12)	0.2037 (12)	0.1554 (8)	0.067 (5)	0.126 (3)
C5F	0.7074 (12)	0.2223 (13)	0.2290 (9)	0.066 (7)	0.126 (3)
C6F	0.6446 (14)	0.1559 (15)	0.2500 (14)	0.066 (9)	0.126 (3)
H6FA	0.622651	0.107216	0.208215	0.099*	0.126 (3)
H6FB	0.673559	0.121739	0.299276	0.099*	0.126 (3)
H6FC	0.596121	0.195014	0.256537	0.099*	0.126 (3)
C7F	0.7750 (13)	0.266 (2)	0.1332 (10)	0.073 (4)	0.126 (3)
H7FA	0.828425	0.229001	0.135149	0.088*	0.126 (3)
H7FB	0.791067	0.322807	0.169679	0.088*	0.126 (3)
C8F	0.733 (2)	0.302 (3)	0.0522 (14)	0.077 (5)	0.126 (3)
H8FA	0.772882	0.345160	0.035791	0.116*	0.126 (3)
H8FB	0.717414	0.245391	0.016407	0.116*	0.126 (3)
H8FC	0.680182	0.338881	0.050821	0.116*	0.126 (3)
O3G	0.744 (3)	0.1657 (14)	0.0058 (19)	0.081 (8)	0.074 (6)
O4G	0.7632 (14)	0.3233 (17)	−0.0192 (14)	0.074 (4)	0.074 (6)
C5G	0.765 (2)	0.2471 (13)	0.0328 (11)	0.076 (4)	0.074 (6)
C6G	0.797 (5)	0.278 (3)	0.1163 (11)	0.073 (6)	0.074 (6)
H6GA	0.808301	0.348988	0.119276	0.109*	0.074 (6)
H6GB	0.753939	0.261724	0.142733	0.109*	0.074 (6)
H6GC	0.851423	0.242428	0.141943	0.109*	0.074 (6)
C7G	0.6795 (16)	0.348 (3)	−0.0692 (17)	0.076 (5)	0.074 (6)
H7GA	0.650069	0.393308	−0.041708	0.092*	0.074 (6)
H7GB	0.643780	0.287739	−0.084069	0.092*	0.074 (6)
C8G	0.690 (3)	0.397 (4)	−0.1404 (16)	0.082 (10)	0.074 (6)
H8GA	0.632482	0.414596	−0.175604	0.123*	0.074 (6)
H8GB	0.724840	0.456788	−0.125167	0.123*	0.074 (6)
H8GC	0.718572	0.351576	−0.167385	0.123*	0.074 (6)
O3H	0.7694 (17)	0.1976 (16)	−0.010 (2)	0.076 (6)	0.087 (6)
O4H	0.7174 (14)	0.3438 (16)	0.009 (2)	0.077 (4)	0.087 (6)
C5H	0.7824 (12)	0.2734 (14)	0.0270 (15)	0.078 (4)	0.087 (6)
C6H	0.8594 (16)	0.302 (3)	0.093 (2)	0.064 (6)	0.087 (6)
H6HA	0.850449	0.368699	0.110952	0.097*	0.087 (6)
H6HB	0.867769	0.255193	0.135774	0.097*	0.087 (6)
H6HC	0.910939	0.302734	0.074322	0.097*	0.087 (6)
C7H	0.6405 (15)	0.3158 (19)	−0.050 (3)	0.073 (5)	0.087 (6)
H7HA	0.612914	0.259028	−0.031591	0.088*	0.087 (6)
H7HB	0.654585	0.295866	−0.097881	0.088*	0.087 (6)
C8H	0.5805 (18)	0.402 (2)	−0.067 (3)	0.083 (11)	0.087 (6)
H8HA	0.527044	0.383567	−0.107310	0.124*	0.087 (6)
H8HB	0.566700	0.420525	−0.018669	0.124*	0.087 (6)
H8HC	0.608230	0.457238	−0.084736	0.124*	0.087 (6)

### [4-(6-bromo-2-methoxyquinolin-3-yl)-3-hydroxy-3-(naphthalen-1-yl)-4-phenylbutyl]dimethylazanium 3-carboxyprop-2-enoate ethyl acetate 0.821-solvate (ethyl_acetate) Atomic displacement parameters (Å^2^)

**Table:**

	*U*^11^	*U*^22^	*U*^33^	*U*^12^	*U*^13^	*U*^23^
Br1A	0.0836 (4)	0.0720 (5)	0.1236 (5)	−0.0269 (4)	0.0664 (4)	−0.0048 (4)
O1A	0.0426 (14)	0.0320 (12)	0.0367 (13)	0.0050 (11)	0.0058 (11)	−0.0062 (10)
O2A	0.0394 (13)	0.0299 (12)	0.0345 (12)	0.0012 (10)	0.0123 (10)	−0.0056 (10)
O3A	0.0420 (14)	0.0427 (15)	0.0509 (15)	−0.0125 (12)	0.0238 (12)	−0.0111 (12)
O4A	0.062 (2)	0.134 (4)	0.0467 (17)	−0.056 (2)	0.0310 (16)	−0.046 (2)
O5A	0.065 (2)	0.134 (4)	0.0478 (18)	−0.052 (2)	0.0327 (16)	−0.028 (2)
O6A	0.0372 (14)	0.0554 (18)	0.0620 (18)	−0.0131 (13)	0.0181 (13)	0.0024 (14)
N1A	0.0275 (12)	0.0277 (13)	0.0323 (13)	0.0048 (10)	0.0097 (10)	0.0043 (11)
N2A	0.0322 (13)	0.0332 (14)	0.0268 (13)	0.0064 (11)	0.0082 (10)	−0.0030 (11)
C1A	0.0289 (15)	0.0336 (16)	0.0262 (14)	0.0083 (12)	0.0082 (12)	0.0034 (12)
C2A	0.0335 (16)	0.0327 (16)	0.0265 (14)	0.0090 (13)	0.0106 (12)	−0.0002 (12)
C3A	0.0281 (14)	0.0293 (15)	0.0273 (14)	0.0058 (12)	0.0095 (12)	−0.0007 (12)
C4A	0.0350 (16)	0.0327 (16)	0.0286 (15)	0.0101 (13)	0.0099 (13)	0.0025 (13)
C5A	0.0288 (15)	0.043 (2)	0.060 (2)	0.0067 (16)	0.0202 (15)	0.0150 (18)
C6A	0.0410 (19)	0.043 (2)	0.0377 (18)	0.0074 (16)	0.0113 (15)	0.0130 (16)
C7A	0.040 (2)	0.038 (2)	0.0272 (19)	0.0172 (18)	0.0143 (17)	0.0034 (16)
C8A	0.054 (3)	0.047 (3)	0.0301 (19)	0.021 (2)	0.0143 (18)	0.0014 (19)
C9A	0.069 (3)	0.058 (3)	0.030 (2)	0.030 (3)	0.022 (2)	0.005 (2)
C10A	0.072 (3)	0.067 (3)	0.056 (3)	0.028 (3)	0.042 (3)	0.010 (3)
C11A	0.063 (3)	0.068 (3)	0.073 (3)	0.013 (3)	0.047 (3)	0.001 (3)
C12A	0.078 (4)	0.106 (5)	0.115 (5)	−0.011 (4)	0.070 (4)	−0.017 (4)
C13A	0.078 (4)	0.107 (5)	0.128 (5)	−0.028 (4)	0.069 (4)	−0.028 (4)
C14A	0.064 (3)	0.083 (4)	0.101 (4)	−0.023 (3)	0.048 (3)	−0.024 (4)
C15A	0.044 (3)	0.055 (3)	0.066 (3)	−0.005 (2)	0.032 (2)	−0.009 (2)
C16A	0.044 (2)	0.052 (3)	0.050 (3)	0.008 (2)	0.029 (2)	0.002 (2)
C7C	0.046 (7)	0.044 (7)	0.039 (6)	0.019 (6)	0.022 (6)	0.003 (6)
C8C	0.050 (7)	0.045 (7)	0.031 (6)	0.024 (6)	0.017 (6)	0.004 (6)
C9C	0.055 (8)	0.047 (8)	0.027 (6)	0.028 (7)	0.017 (6)	0.008 (7)
C10C	0.059 (8)	0.054 (8)	0.037 (7)	0.029 (7)	0.031 (7)	0.007 (7)
C11C	0.058 (6)	0.061 (6)	0.049 (6)	0.019 (5)	0.037 (5)	0.000 (6)
C12C	0.066 (7)	0.083 (7)	0.076 (7)	0.002 (6)	0.053 (6)	−0.004 (7)
C13C	0.063 (6)	0.090 (7)	0.099 (6)	−0.014 (6)	0.060 (6)	−0.011 (6)
C14C	0.062 (6)	0.081 (6)	0.091 (6)	−0.013 (6)	0.047 (6)	−0.011 (6)
C15C	0.053 (6)	0.068 (7)	0.072 (7)	−0.005 (6)	0.037 (6)	−0.010 (7)
C16C	0.050 (5)	0.053 (5)	0.049 (5)	0.010 (5)	0.031 (5)	0.000 (5)
C17A	0.0354 (17)	0.0407 (19)	0.0325 (16)	0.0158 (15)	0.0157 (13)	0.0082 (14)
C18A	0.047 (2)	0.043 (2)	0.049 (2)	0.0174 (17)	0.0167 (18)	0.0028 (17)
C19A	0.065 (3)	0.041 (2)	0.071 (3)	0.022 (2)	0.032 (2)	0.010 (2)
C20A	0.062 (3)	0.065 (3)	0.059 (3)	0.040 (3)	0.031 (2)	0.023 (2)
C21A	0.042 (2)	0.089 (4)	0.061 (3)	0.031 (3)	0.012 (2)	0.015 (3)
C22A	0.0366 (19)	0.070 (3)	0.045 (2)	0.022 (2)	0.0127 (16)	0.005 (2)
C23A	0.0287 (14)	0.0304 (15)	0.0226 (13)	0.0070 (12)	0.0062 (11)	−0.0004 (12)
C24A	0.0322 (16)	0.0330 (16)	0.0282 (15)	0.0058 (13)	0.0093 (12)	−0.0006 (13)
C25A	0.0284 (15)	0.0373 (17)	0.0284 (15)	0.0025 (13)	0.0074 (12)	−0.0011 (13)
C26A	0.0352 (18)	0.043 (2)	0.0417 (19)	−0.0009 (15)	0.0105 (15)	−0.0024 (16)
C27A	0.0369 (19)	0.054 (2)	0.044 (2)	−0.0100 (17)	0.0156 (16)	0.0009 (18)
C28A	0.0356 (18)	0.065 (3)	0.0338 (18)	−0.0029 (18)	0.0156 (14)	−0.0026 (17)
C29A	0.0305 (16)	0.056 (2)	0.0291 (16)	0.0044 (15)	0.0108 (13)	−0.0076 (15)
C30A	0.0254 (14)	0.0433 (19)	0.0220 (14)	0.0052 (13)	0.0049 (11)	−0.0009 (13)
C31A	0.0299 (15)	0.0303 (16)	0.0254 (14)	0.0060 (12)	0.0073 (12)	−0.0018 (12)
C32A	0.057 (2)	0.0321 (19)	0.049 (2)	0.0020 (16)	0.0189 (18)	−0.0102 (16)
C33A	0.046 (2)	0.047 (2)	0.0398 (19)	−0.0182 (17)	0.0204 (16)	−0.0123 (16)
C34A	0.0427 (19)	0.043 (2)	0.0336 (17)	−0.0117 (16)	0.0160 (14)	−0.0074 (15)
C35A	0.0419 (19)	0.044 (2)	0.0361 (18)	−0.0135 (16)	0.0111 (15)	−0.0076 (15)
C36A	0.0405 (19)	0.048 (2)	0.043 (2)	−0.0133 (17)	0.0145 (16)	−0.0021 (17)
Br1B	0.0847 (4)	0.0803 (6)	0.1601 (7)	−0.0043 (4)	0.0751 (5)	0.0374 (5)
O1B	0.0402 (13)	0.0300 (12)	0.0476 (14)	−0.0008 (11)	0.0175 (11)	−0.0049 (11)
O2B	0.0367 (13)	0.0376 (13)	0.0344 (12)	0.0057 (10)	0.0139 (10)	−0.0007 (10)
O3B	0.0568 (18)	0.0490 (17)	0.0620 (18)	−0.0169 (14)	0.0362 (15)	−0.0149 (14)
O4B	0.0549 (19)	0.084 (3)	0.0479 (17)	−0.0317 (17)	0.0275 (14)	−0.0251 (16)
O5B	0.0510 (17)	0.085 (2)	0.0420 (15)	−0.0259 (17)	0.0226 (13)	−0.0086 (15)
O6B	0.0419 (15)	0.0460 (16)	0.0649 (18)	−0.0101 (13)	0.0162 (14)	0.0042 (14)
N1B	0.0288 (12)	0.0358 (15)	0.0325 (13)	0.0077 (11)	0.0115 (10)	0.0083 (12)
N2B	0.0304 (13)	0.0457 (17)	0.0252 (13)	0.0082 (12)	0.0065 (10)	0.0014 (12)
C1B	0.0300 (15)	0.0336 (17)	0.0320 (16)	0.0095 (13)	0.0114 (13)	0.0069 (13)
C2B	0.0326 (16)	0.0279 (15)	0.0333 (16)	0.0071 (12)	0.0142 (13)	0.0016 (12)
C3B	0.0273 (14)	0.0295 (15)	0.0257 (14)	0.0077 (12)	0.0081 (11)	0.0014 (12)
C4B	0.0336 (16)	0.0310 (16)	0.0317 (16)	0.0066 (13)	0.0094 (13)	−0.0012 (13)
C5B	0.0339 (17)	0.052 (2)	0.061 (2)	0.0102 (18)	0.0239 (16)	0.016 (2)
C6B	0.048 (2)	0.061 (3)	0.042 (2)	0.016 (2)	0.0154 (17)	0.0232 (19)
C7B	0.0347 (17)	0.0351 (17)	0.0338 (17)	0.0110 (14)	0.0146 (14)	0.0031 (13)
C8B	0.047 (2)	0.041 (2)	0.0349 (18)	0.0100 (17)	0.0148 (15)	−0.0038 (15)
C9B	0.056 (2)	0.050 (2)	0.0381 (19)	0.021 (2)	0.0189 (17)	−0.0019 (17)
C10B	0.054 (2)	0.055 (2)	0.047 (2)	0.025 (2)	0.0320 (19)	0.0110 (18)
C11B	0.0411 (19)	0.048 (2)	0.056 (2)	0.0145 (17)	0.0265 (18)	0.0106 (18)
C12B	0.048 (2)	0.067 (3)	0.071 (3)	0.006 (2)	0.035 (2)	0.016 (3)
C13B	0.043 (2)	0.066 (3)	0.084 (3)	−0.003 (2)	0.031 (2)	0.008 (3)
C14B	0.044 (2)	0.046 (2)	0.060 (2)	−0.0028 (19)	0.0175 (18)	0.004 (2)
C15B	0.0354 (18)	0.043 (2)	0.046 (2)	0.0044 (16)	0.0153 (15)	0.0038 (17)
C16B	0.0343 (16)	0.0372 (18)	0.0420 (19)	0.0118 (14)	0.0185 (14)	0.0074 (15)
C17B	0.030 (4)	0.034 (4)	0.043 (5)	0.008 (3)	0.014 (3)	0.009 (4)
C18B	0.036 (4)	0.043 (4)	0.062 (5)	0.011 (3)	0.001 (4)	0.001 (4)
C19B	0.045 (5)	0.043 (4)	0.074 (5)	0.014 (4)	0.004 (4)	−0.003 (4)
C20B	0.036 (4)	0.044 (5)	0.064 (5)	0.015 (4)	0.016 (4)	0.013 (4)
C21B	0.034 (4)	0.047 (5)	0.055 (5)	0.014 (3)	0.011 (3)	0.007 (4)
C22B	0.025 (3)	0.036 (4)	0.043 (4)	0.006 (3)	0.009 (3)	0.007 (3)
C17D	0.029 (5)	0.034 (5)	0.031 (5)	0.019 (4)	0.016 (4)	0.008 (4)
C18D	0.041 (5)	0.032 (4)	0.042 (5)	0.020 (4)	0.014 (4)	0.000 (4)
C19D	0.048 (5)	0.045 (5)	0.052 (5)	0.025 (4)	0.018 (4)	0.003 (4)
C20D	0.045 (6)	0.046 (5)	0.049 (5)	0.025 (4)	0.013 (4)	0.006 (4)
C21D	0.041 (5)	0.046 (6)	0.056 (5)	0.022 (5)	0.007 (4)	−0.003 (5)
C22D	0.035 (5)	0.043 (6)	0.045 (5)	0.014 (4)	0.014 (4)	0.003 (5)
C23B	0.0289 (15)	0.0360 (17)	0.0308 (15)	0.0129 (13)	0.0101 (12)	0.0070 (13)
C24B	0.0342 (17)	0.0365 (18)	0.0382 (18)	0.0105 (14)	0.0125 (14)	0.0094 (14)
C25B	0.0307 (16)	0.051 (2)	0.0335 (17)	0.0069 (15)	0.0086 (13)	0.0112 (16)
C26B	0.040 (2)	0.052 (2)	0.061 (3)	0.0063 (18)	0.0179 (18)	0.018 (2)
C27B	0.048 (2)	0.068 (3)	0.051 (2)	−0.005 (2)	0.0160 (19)	0.022 (2)
C28B	0.0349 (19)	0.078 (3)	0.0365 (19)	0.005 (2)	0.0121 (15)	0.011 (2)
C29B	0.0323 (17)	0.066 (3)	0.0276 (16)	0.0048 (17)	0.0095 (13)	0.0051 (16)
C30B	0.0279 (15)	0.052 (2)	0.0231 (14)	0.0088 (14)	0.0044 (12)	0.0075 (14)
C31B	0.0268 (14)	0.0378 (17)	0.0252 (14)	0.0088 (13)	0.0047 (11)	0.0037 (13)
C32B	0.058 (3)	0.046 (2)	0.054 (2)	0.0049 (19)	0.023 (2)	−0.0095 (19)
C33B	0.054 (2)	0.0350 (18)	0.048 (2)	−0.0104 (17)	0.0260 (18)	−0.0118 (16)
C34B	0.056 (2)	0.041 (2)	0.0410 (19)	−0.0078 (17)	0.0252 (17)	−0.0093 (16)
C35B	0.048 (2)	0.0385 (19)	0.0367 (18)	−0.0057 (16)	0.0117 (15)	−0.0069 (15)
C36B	0.045 (2)	0.0349 (18)	0.047 (2)	−0.0070 (16)	0.0173 (16)	0.0031 (15)
O1	0.110 (4)	0.072 (4)	0.160 (6)	−0.016 (3)	0.024 (4)	0.036 (4)
O2	0.081 (3)	0.084 (3)	0.140 (4)	−0.010 (3)	0.054 (3)	−0.014 (3)
C1	0.078 (4)	0.048 (3)	0.143 (6)	−0.010 (3)	0.043 (4)	0.013 (4)
C2	0.125 (7)	0.096 (6)	0.149 (8)	−0.048 (6)	0.061 (6)	0.008 (6)
C3	0.097 (5)	0.098 (6)	0.144 (7)	0.001 (5)	0.052 (5)	−0.020 (5)
C4	0.113 (7)	0.128 (8)	0.167 (9)	−0.012 (6)	0.072 (7)	−0.047 (7)
O1E	0.084 (13)	0.096 (13)	0.150 (14)	−0.017 (13)	0.037 (13)	−0.001 (13)
O2E	0.101 (8)	0.090 (8)	0.149 (9)	−0.001 (8)	0.045 (8)	−0.009 (8)
C1E	0.092 (8)	0.079 (8)	0.153 (9)	−0.006 (8)	0.041 (8)	−0.003 (8)
C2E	0.118 (14)	0.084 (14)	0.163 (15)	−0.010 (14)	0.031 (14)	0.009 (14)
C3E	0.094 (9)	0.096 (9)	0.143 (9)	−0.004 (8)	0.057 (9)	−0.020 (8)
C4E	0.101 (19)	0.094 (19)	0.137 (19)	0.007 (17)	0.050 (18)	−0.051 (18)
O3	0.045 (8)	0.070 (10)	0.130 (10)	−0.002 (8)	0.027 (8)	−0.004 (9)
O4	0.049 (6)	0.057 (6)	0.109 (7)	−0.003 (5)	0.021 (5)	0.006 (6)
C5	0.050 (6)	0.059 (7)	0.110 (7)	0.002 (6)	0.019 (6)	0.002 (6)
C6	0.059 (10)	0.067 (11)	0.099 (11)	0.003 (10)	0.014 (10)	0.008 (10)
C7	0.052 (7)	0.061 (8)	0.109 (9)	−0.003 (7)	0.018 (7)	0.006 (7)
C8	0.050 (9)	0.058 (11)	0.110 (11)	0.006 (10)	0.003 (9)	0.017 (10)
O3E	0.038 (7)	0.057 (9)	0.143 (11)	−0.005 (7)	0.022 (8)	−0.014 (9)
O4E	0.053 (5)	0.061 (6)	0.111 (7)	−0.003 (5)	0.014 (5)	0.008 (6)
C5E	0.050 (6)	0.061 (7)	0.111 (7)	0.004 (5)	0.018 (6)	0.000 (6)
C6E	0.053 (9)	0.050 (10)	0.115 (11)	0.004 (9)	−0.002 (10)	−0.011 (10)
C7E	0.048 (6)	0.063 (7)	0.104 (8)	0.003 (6)	0.020 (6)	0.005 (7)
C8E	0.050 (9)	0.050 (10)	0.106 (11)	−0.004 (10)	0.007 (9)	0.002 (9)
O3F	0.067 (17)	0.083 (19)	0.11 (2)	−0.020 (15)	0.000 (16)	−0.005 (17)
O4F	0.052 (8)	0.063 (10)	0.095 (10)	0.013 (8)	0.035 (8)	0.006 (9)
C5F	0.045 (11)	0.065 (13)	0.090 (14)	0.015 (11)	0.025 (11)	−0.001 (12)
C6F	0.058 (18)	0.057 (18)	0.09 (2)	0.019 (15)	0.032 (17)	0.004 (17)
C7F	0.050 (6)	0.059 (7)	0.109 (8)	0.006 (6)	0.020 (6)	0.005 (7)
C8F	0.054 (8)	0.062 (9)	0.112 (10)	−0.004 (8)	0.019 (8)	0.004 (9)
O3G	0.053 (12)	0.068 (13)	0.113 (13)	−0.003 (11)	0.013 (12)	−0.002 (12)
O4G	0.050 (6)	0.060 (7)	0.109 (7)	0.001 (5)	0.019 (6)	0.005 (6)
C5G	0.050 (6)	0.061 (7)	0.112 (7)	0.000 (5)	0.018 (6)	0.005 (6)
C6G	0.048 (8)	0.059 (9)	0.111 (10)	0.004 (8)	0.023 (9)	0.005 (9)
C7G	0.053 (7)	0.062 (8)	0.108 (9)	0.000 (7)	0.014 (7)	0.005 (8)
C8G	0.055 (16)	0.065 (17)	0.121 (18)	−0.002 (16)	0.020 (16)	0.014 (17)
O3H	0.048 (10)	0.058 (10)	0.112 (11)	−0.008 (9)	0.011 (9)	0.003 (10)
O4H	0.053 (6)	0.062 (7)	0.110 (8)	−0.001 (6)	0.014 (6)	0.005 (6)
C5H	0.053 (6)	0.062 (7)	0.114 (7)	0.002 (5)	0.016 (6)	0.003 (6)
C6H	0.036 (9)	0.054 (10)	0.110 (11)	−0.005 (9)	0.032 (9)	−0.001 (10)
C7H	0.049 (7)	0.059 (8)	0.107 (9)	0.002 (7)	0.016 (7)	0.005 (7)
C8H	0.057 (18)	0.07 (2)	0.11 (2)	−0.019 (17)	0.019 (18)	0.002 (19)

### [4-(6-bromo-2-methoxyquinolin-3-yl)-3-hydroxy-3-(naphthalen-1-yl)-4-phenylbutyl]dimethylazanium 3-carboxyprop-2-enoate ethyl acetate 0.821-solvate (ethyl_acetate) Geometric parameters (Å, º)

**Table:**

Br1A—C27A	1.906 (4)	C10B—H10B	0.9500
O1A—C2A	1.415 (5)	C11B—C12B	1.420 (7)
O1A—H1AB	0.84 (6)	C11B—C16B	1.441 (5)
O2A—C31A	1.348 (4)	C12B—C13B	1.346 (8)
O2A—C32A	1.437 (4)	C12B—H12B	0.9500
O3A—C33A	1.245 (5)	C13B—C14B	1.403 (6)
O4A—C33A	1.265 (5)	C13B—H13B	0.9500
O4A—H5A	1.33 (10)	C14B—C15B	1.391 (6)
O5A—C36A	1.292 (5)	C14B—H14B	0.9500
O5A—H5A	1.11 (10)	C15B—C16B	1.420 (6)
O6A—C36A	1.216 (5)	C15B—H15B	0.9500
N1A—C6A	1.490 (4)	C17B—C22B	1.379 (11)
N1A—C5A	1.494 (4)	C17B—C18B	1.424 (13)
N1A—C4A	1.508 (4)	C18B—C19B	1.405 (12)
N1A—H1A	1.0000	C18B—H18B	0.9500
N2A—C31A	1.302 (4)	C19B—C20B	1.361 (12)
N2A—C30A	1.365 (5)	C19B—H19B	0.9500
C1A—C17A	1.521 (5)	C20B—C21B	1.373 (12)
C1A—C23A	1.523 (4)	C20B—H20B	0.9500
C1A—C2A	1.582 (4)	C21B—C22B	1.390 (10)
C1A—H1AA	1.0000	C21B—H21B	0.9500
C2A—C7C	1.52 (2)	C22B—H22B	0.9500
C2A—C3A	1.544 (4)	C17D—C22D	1.375 (14)
C2A—C7A	1.550 (5)	C17D—C18D	1.402 (15)
C3A—C4A	1.523 (4)	C18D—C19D	1.405 (12)
C3A—H3AA	0.9900	C18D—H18D	0.9500
C3A—H3AB	0.9900	C19D—C20D	1.372 (15)
C4A—H4AA	0.9900	C19D—H19D	0.9500
C4A—H4AB	0.9900	C20D—C21D	1.376 (16)
C5A—H5AA	0.9800	C20D—H20D	0.9500
C5A—H5AB	0.9800	C21D—C22D	1.397 (14)
C5A—H5AC	0.9800	C21D—H21D	0.9500
C6A—H6AA	0.9800	C22D—H22D	0.9500
C6A—H6AB	0.9800	C23B—C24B	1.364 (5)
C6A—H6AC	0.9800	C23B—C31B	1.447 (5)
C7A—C8A	1.382 (6)	C24B—C25B	1.429 (5)
C7A—C16A	1.445 (7)	C24B—H24B	0.9500
C8A—C9A	1.414 (7)	C25B—C30B	1.412 (6)
C8A—H8A	0.9500	C25B—C26B	1.419 (6)
C9A—C10A	1.366 (10)	C26B—C27B	1.377 (6)
C9A—H9A	0.9500	C26B—H26B	0.9500
C10A—C11A	1.407 (9)	C27B—C28B	1.389 (8)
C10A—H10A	0.9500	C28B—C29B	1.382 (7)
C11A—C16A	1.436 (6)	C28B—H28B	0.9500
C11A—C12A	1.438 (10)	C29B—C30B	1.423 (5)
C12A—C13A	1.336 (12)	C29B—H29B	0.9500
C12A—H12A	0.9500	C32B—H32D	0.9800
C13A—C14A	1.390 (9)	C32B—H32E	0.9800
C13A—H13A	0.9500	C32B—H32F	0.9800
C14A—C15A	1.401 (8)	C33B—C34B	1.497 (6)
C14A—H14A	0.9500	C34B—C35B	1.331 (6)
C15A—C16A	1.410 (8)	C34B—H34B	0.9500
C15A—H15A	0.9500	C35B—C36B	1.497 (6)
C7C—C8C	1.37 (2)	C35B—H35B	0.9500
C7C—C16C	1.42 (2)	O1—C1	1.209 (8)
C8C—C9C	1.41 (2)	O2—C1	1.382 (8)
C8C—H8C	0.9500	O2—C3	1.421 (10)
C9C—C10C	1.37 (2)	C1—C2	1.484 (10)
C9C—H9C	0.9500	C2—H2A	0.9800
C10C—C11C	1.42 (2)	C2—H2B	0.9800
C10C—H10C	0.9500	C2—H2C	0.9800
C11C—C12C	1.42 (2)	C3—C4	1.484 (8)
C11C—C16C	1.44 (2)	C3—H3A	0.9900
C12C—C13C	1.36 (2)	C3—H3B	0.9900
C12C—H12C	0.9500	C4—H4A	0.9800
C13C—C14C	1.39 (2)	C4—H4B	0.9800
C13C—H13C	0.9500	C4—H4C	0.9800
C14C—C15C	1.40 (2)	O1E—C1E	1.209 (8)
C14C—H14C	0.9500	O2E—C1E	1.383 (8)
C15C—C16C	1.41 (2)	O2E—C3E	1.421 (10)
C15C—H15C	0.9500	C1E—C2E	1.485 (10)
C17A—C22A	1.384 (6)	C2E—H2EA	0.9800
C17A—C18A	1.403 (6)	C2E—H2EB	0.9800
C18A—C19A	1.406 (5)	C2E—H2EC	0.9800
C18A—H18A	0.9500	C3E—C4E	1.485 (8)
C19A—C20A	1.374 (7)	C3E—H3EA	0.9900
C19A—H19A	0.9500	C3E—H3EB	0.9900
C20A—C21A	1.365 (8)	C4E—H4EA	0.9800
C20A—H20A	0.9500	C4E—H4EB	0.9800
C21A—C22A	1.392 (6)	C4E—H4EC	0.9800
C21A—H21A	0.9500	O3—C5	1.209 (8)
C22A—H22A	0.9500	O4—C5	1.383 (8)
C23A—C24A	1.353 (5)	O4—C7	1.421 (10)
C23A—C31A	1.451 (4)	C5—C6	1.484 (10)
C24A—C25A	1.419 (5)	C6—H6A	0.9800
C24A—H24A	0.9500	C6—H6B	0.9800
C25A—C30A	1.416 (5)	C6—H6C	0.9800
C25A—C26A	1.418 (5)	C7—C8	1.485 (8)
C26A—C27A	1.360 (6)	C7—H7A	0.9900
C26A—H26A	0.9500	C7—H7B	0.9900
C27A—C28A	1.402 (7)	C8—H8D	0.9800
C28A—C29A	1.378 (6)	C8—H8E	0.9800
C28A—H28A	0.9500	C8—H8F	0.9800
C29A—C30A	1.413 (5)	O3E—C5E	1.209 (8)
C29A—H29A	0.9500	O4E—C5E	1.383 (8)
C32A—H32A	0.9800	O4E—C7E	1.421 (10)
C32A—H32B	0.9800	C5E—C6E	1.485 (10)
C32A—H32C	0.9800	C6E—H6EA	0.9800
C33A—C34A	1.495 (5)	C6E—H6EB	0.9800
C34A—C35A	1.337 (5)	C6E—H6EC	0.9800
C34A—H34A	0.9500	C7E—C8E	1.485 (8)
C35A—C36A	1.497 (5)	C7E—H7EA	0.9900
C35A—H35A	0.9500	C7E—H7EB	0.9900
Br1B—C27B	1.906 (5)	C8E—H8EA	0.9800
O1B—C2B	1.427 (4)	C8E—H8EB	0.9800
O1B—H1BB	0.92 (6)	C8E—H8EC	0.9800
O2B—C31B	1.350 (4)	O3F—C5F	1.209 (8)
O2B—C32B	1.432 (5)	O4F—C5F	1.383 (8)
O3B—C33B	1.241 (5)	O4F—C7F	1.421 (10)
O4B—C33B	1.266 (5)	C5F—C6F	1.484 (10)
O4B—H5B	1.29 (8)	C6F—H6FA	0.9800
O5B—C36B	1.293 (5)	C6F—H6FB	0.9800
O5B—H5B	1.17 (8)	C6F—H6FC	0.9800
O6B—C36B	1.216 (5)	C7F—C8F	1.485 (8)
N1B—C6B	1.493 (5)	C7F—H7FA	0.9900
N1B—C5B	1.496 (4)	C7F—H7FB	0.9900
N1B—C4B	1.504 (4)	C8F—H8FA	0.9800
N1B—H1B	1.0000	C8F—H8FB	0.9800
N2B—C31B	1.311 (4)	C8F—H8FC	0.9800
N2B—C30B	1.354 (5)	O3G—C5G	1.209 (8)
C1B—C17B	1.512 (14)	O4G—C5G	1.383 (8)
C1B—C23B	1.518 (5)	O4G—C7G	1.421 (10)
C1B—C17D	1.558 (16)	C5G—C6G	1.484 (10)
C1B—C2B	1.582 (5)	C6G—H6GA	0.9800
C1B—H1BA	1.0000	C6G—H6GB	0.9800
C2B—C7B	1.539 (5)	C6G—H6GC	0.9800
C2B—C3B	1.543 (4)	C7G—C8G	1.485 (8)
C3B—C4B	1.517 (4)	C7G—H7GA	0.9900
C3B—H3BA	0.9900	C7G—H7GB	0.9900
C3B—H3BB	0.9900	C8G—H8GA	0.9800
C4B—H4BA	0.9900	C8G—H8GB	0.9800
C4B—H4BB	0.9900	C8G—H8GC	0.9800
C5B—H5BA	0.9800	O3H—C5H	1.209 (8)
C5B—H5BB	0.9800	O4H—C5H	1.383 (8)
C5B—H5BC	0.9800	O4H—C7H	1.421 (10)
C6B—H6BA	0.9800	C5H—C6H	1.484 (10)
C6B—H6BB	0.9800	C6H—H6HA	0.9800
C6B—H6BC	0.9800	C6H—H6HB	0.9800
C7B—C8B	1.385 (5)	C6H—H6HC	0.9800
C7B—C16B	1.440 (5)	C7H—C8H	1.485 (8)
C8B—C9B	1.411 (5)	C7H—H7HA	0.9900
C8B—H8B	0.9500	C7H—H7HB	0.9900
C9B—C10B	1.354 (7)	C8H—H8HA	0.9800
C9B—H9B	0.9500	C8H—H8HB	0.9800
C10B—C11B	1.431 (7)	C8H—H8HC	0.9800
			
C2A—O1A—H1AB	115 (4)	C15B—C14B—H14B	119.6
C31A—O2A—C32A	117.1 (3)	C13B—C14B—H14B	119.6
C33A—O4A—H5A	109 (4)	C14B—C15B—C16B	122.2 (4)
C36A—O5A—H5A	107 (4)	C14B—C15B—H15B	118.9
C6A—N1A—C5A	110.0 (3)	C16B—C15B—H15B	118.9
C6A—N1A—C4A	111.2 (3)	C15B—C16B—C7B	125.4 (3)
C5A—N1A—C4A	112.5 (3)	C15B—C16B—C11B	115.5 (4)
C6A—N1A—H1A	107.7	C7B—C16B—C11B	119.1 (4)
C5A—N1A—H1A	107.7	C22B—C17B—C18B	118.3 (9)
C4A—N1A—H1A	107.7	C22B—C17B—C1B	122.1 (9)
C31A—N2A—C30A	117.6 (3)	C18B—C17B—C1B	119.6 (8)
C17A—C1A—C23A	112.3 (3)	C19B—C18B—C17B	119.4 (9)
C17A—C1A—C2A	114.1 (3)	C19B—C18B—H18B	120.3
C23A—C1A—C2A	110.4 (3)	C17B—C18B—H18B	120.3
C17A—C1A—H1AA	106.5	C20B—C19B—C18B	120.6 (9)
C23A—C1A—H1AA	106.5	C20B—C19B—H19B	119.7
C2A—C1A—H1AA	106.5	C18B—C19B—H19B	119.7
O1A—C2A—C7C	92.3 (11)	C19B—C20B—C21B	120.2 (8)
O1A—C2A—C3A	108.3 (3)	C19B—C20B—H20B	119.9
C7C—C2A—C3A	118.3 (13)	C21B—C20B—H20B	119.9
O1A—C2A—C7A	109.2 (3)	C20B—C21B—C22B	120.8 (8)
C3A—C2A—C7A	110.6 (3)	C20B—C21B—H21B	119.6
O1A—C2A—C1A	109.6 (3)	C22B—C21B—H21B	119.6
C7C—C2A—C1A	117.0 (13)	C17B—C22B—C21B	120.8 (9)
C3A—C2A—C1A	109.5 (3)	C17B—C22B—H22B	119.6
C7A—C2A—C1A	109.6 (3)	C21B—C22B—H22B	119.6
C4A—C3A—C2A	113.3 (3)	C22D—C17D—C18D	119.1 (11)
C4A—C3A—H3AA	108.9	C22D—C17D—C1B	114.6 (11)
C2A—C3A—H3AA	108.9	C18D—C17D—C1B	126.3 (11)
C4A—C3A—H3AB	108.9	C17D—C18D—C19D	117.5 (11)
C2A—C3A—H3AB	108.9	C17D—C18D—H18D	121.3
H3AA—C3A—H3AB	107.7	C19D—C18D—H18D	121.3
N1A—C4A—C3A	110.6 (2)	C20D—C19D—C18D	122.1 (11)
N1A—C4A—H4AA	109.5	C20D—C19D—H19D	119.0
C3A—C4A—H4AA	109.5	C18D—C19D—H19D	119.0
N1A—C4A—H4AB	109.5	C19D—C20D—C21D	120.7 (10)
C3A—C4A—H4AB	109.5	C19D—C20D—H20D	119.7
H4AA—C4A—H4AB	108.1	C21D—C20D—H20D	119.7
N1A—C5A—H5AA	109.5	C20D—C21D—C22D	117.4 (11)
N1A—C5A—H5AB	109.5	C20D—C21D—H21D	121.3
H5AA—C5A—H5AB	109.5	C22D—C21D—H21D	121.3
N1A—C5A—H5AC	109.5	C17D—C22D—C21D	123.1 (12)
H5AA—C5A—H5AC	109.5	C17D—C22D—H22D	118.4
H5AB—C5A—H5AC	109.5	C21D—C22D—H22D	118.4
N1A—C6A—H6AA	109.5	C24B—C23B—C31B	115.2 (3)
N1A—C6A—H6AB	109.5	C24B—C23B—C1B	124.6 (3)
H6AA—C6A—H6AB	109.5	C31B—C23B—C1B	120.1 (3)
N1A—C6A—H6AC	109.5	C23B—C24B—C25B	120.8 (3)
H6AA—C6A—H6AC	109.5	C23B—C24B—H24B	119.6
H6AB—C6A—H6AC	109.5	C25B—C24B—H24B	119.6
C8A—C7A—C16A	117.9 (4)	C30B—C25B—C26B	120.3 (4)
C8A—C7A—C2A	117.3 (4)	C30B—C25B—C24B	117.6 (4)
C16A—C7A—C2A	124.8 (4)	C26B—C25B—C24B	122.1 (4)
C7A—C8A—C9A	122.9 (6)	C27B—C26B—C25B	118.3 (5)
C7A—C8A—H8A	118.6	C27B—C26B—H26B	120.8
C9A—C8A—H8A	118.6	C25B—C26B—H26B	120.8
C10A—C9A—C8A	119.6 (5)	C26B—C27B—C28B	122.7 (4)
C10A—C9A—H9A	120.2	C26B—C27B—Br1B	118.4 (4)
C8A—C9A—H9A	120.2	C28B—C27B—Br1B	118.9 (3)
C9A—C10A—C11A	120.8 (5)	C29B—C28B—C27B	119.5 (4)
C9A—C10A—H10A	119.6	C29B—C28B—H28B	120.3
C11A—C10A—H10A	119.6	C27B—C28B—H28B	120.3
C10A—C11A—C16A	120.1 (6)	C28B—C29B—C30B	120.4 (4)
C10A—C11A—C12A	120.5 (5)	C28B—C29B—H29B	119.8
C16A—C11A—C12A	119.3 (6)	C30B—C29B—H29B	119.8
C13A—C12A—C11A	122.0 (6)	N2B—C30B—C25B	122.7 (3)
C13A—C12A—H12A	119.0	N2B—C30B—C29B	118.5 (4)
C11A—C12A—H12A	119.0	C25B—C30B—C29B	118.8 (4)
C12A—C13A—C14A	120.0 (7)	N2B—C31B—O2B	119.1 (3)
C12A—C13A—H13A	120.0	N2B—C31B—C23B	126.2 (3)
C14A—C13A—H13A	120.0	O2B—C31B—C23B	114.6 (3)
C13A—C14A—C15A	120.2 (7)	O2B—C32B—H32D	109.5
C13A—C14A—H14A	119.9	O2B—C32B—H32E	109.5
C15A—C14A—H14A	119.9	H32D—C32B—H32E	109.5
C14A—C15A—C16A	122.2 (5)	O2B—C32B—H32F	109.5
C14A—C15A—H15A	118.9	H32D—C32B—H32F	109.5
C16A—C15A—H15A	118.9	H32E—C32B—H32F	109.5
C15A—C16A—C11A	116.3 (5)	O3B—C33B—O4B	122.8 (4)
C15A—C16A—C7A	125.0 (4)	O3B—C33B—C34B	117.7 (4)
C11A—C16A—C7A	118.8 (5)	O4B—C33B—C34B	119.5 (4)
C8C—C7C—C16C	123 (2)	C35B—C34B—C33B	130.8 (4)
C8C—C7C—C2A	126 (2)	C35B—C34B—H34B	114.6
C16C—C7C—C2A	111.5 (18)	C33B—C34B—H34B	114.6
C7C—C8C—C9C	120 (2)	C34B—C35B—C36B	130.6 (4)
C7C—C8C—H8C	120.0	C34B—C35B—H35B	114.7
C9C—C8C—H8C	120.0	C36B—C35B—H35B	114.7
C10C—C9C—C8C	121 (2)	O6B—C36B—O5B	122.5 (4)
C10C—C9C—H9C	119.7	O6B—C36B—C35B	117.6 (4)
C8C—C9C—H9C	119.7	O5B—C36B—C35B	119.9 (3)
C9C—C10C—C11C	120 (2)	C1—O2—C3	115.1 (6)
C9C—C10C—H10C	120.1	O1—C1—O2	117.7 (7)
C11C—C10C—H10C	120.1	O1—C1—C2	128.9 (7)
C12C—C11C—C10C	120 (2)	O2—C1—C2	113.3 (6)
C12C—C11C—C16C	119 (2)	C1—C2—H2A	109.5
C10C—C11C—C16C	121 (2)	C1—C2—H2B	109.5
C13C—C12C—C11C	123 (2)	H2A—C2—H2B	109.5
C13C—C12C—H12C	118.6	C1—C2—H2C	109.5
C11C—C12C—H12C	118.6	H2A—C2—H2C	109.5
C12C—C13C—C14C	117 (3)	H2B—C2—H2C	109.5
C12C—C13C—H13C	121.5	O2—C3—C4	108.4 (7)
C14C—C13C—H13C	121.5	O2—C3—H3A	110.0
C13C—C14C—C15C	122 (3)	C4—C3—H3A	110.0
C13C—C14C—H14C	119.1	O2—C3—H3B	110.0
C15C—C14C—H14C	119.1	C4—C3—H3B	110.0
C14C—C15C—C16C	121 (2)	H3A—C3—H3B	108.4
C14C—C15C—H15C	119.3	C3—C4—H4A	109.5
C16C—C15C—H15C	119.3	C3—C4—H4B	109.5
C15C—C16C—C7C	128 (2)	H4A—C4—H4B	109.5
C15C—C16C—C11C	116 (2)	C3—C4—H4C	109.5
C7C—C16C—C11C	116 (2)	H4A—C4—H4C	109.5
C22A—C17A—C18A	118.3 (4)	H4B—C4—H4C	109.5
C22A—C17A—C1A	117.8 (4)	C1E—O2E—C3E	114.9 (6)
C18A—C17A—C1A	123.9 (3)	O1E—C1E—O2E	117.6 (7)
C17A—C18A—C19A	119.9 (4)	O1E—C1E—C2E	128.8 (7)
C17A—C18A—H18A	120.1	O2E—C1E—C2E	113.4 (6)
C19A—C18A—H18A	120.1	C1E—C2E—H2EA	109.5
C20A—C19A—C18A	120.8 (5)	C1E—C2E—H2EB	109.5
C20A—C19A—H19A	119.6	H2EA—C2E—H2EB	109.5
C18A—C19A—H19A	119.6	C1E—C2E—H2EC	109.5
C21A—C20A—C19A	119.1 (4)	H2EA—C2E—H2EC	109.5
C21A—C20A—H20A	120.5	H2EB—C2E—H2EC	109.5
C19A—C20A—H20A	120.5	O2E—C3E—C4E	108.3 (7)
C20A—C21A—C22A	121.5 (5)	O2E—C3E—H3EA	110.0
C20A—C21A—H21A	119.2	C4E—C3E—H3EA	110.0
C22A—C21A—H21A	119.2	O2E—C3E—H3EB	110.0
C17A—C22A—C21A	120.5 (5)	C4E—C3E—H3EB	110.0
C17A—C22A—H22A	119.8	H3EA—C3E—H3EB	108.4
C21A—C22A—H22A	119.8	C3E—C4E—H4EA	109.5
C24A—C23A—C31A	116.1 (3)	C3E—C4E—H4EB	109.5
C24A—C23A—C1A	124.4 (3)	H4EA—C4E—H4EB	109.5
C31A—C23A—C1A	119.6 (3)	C3E—C4E—H4EC	109.5
C23A—C24A—C25A	120.8 (3)	H4EA—C4E—H4EC	109.5
C23A—C24A—H24A	119.6	H4EB—C4E—H4EC	109.5
C25A—C24A—H24A	119.6	C5—O4—C7	115.0 (6)
C30A—C25A—C26A	119.2 (3)	O3—C5—O4	117.7 (7)
C30A—C25A—C24A	117.8 (3)	O3—C5—C6	128.8 (7)
C26A—C25A—C24A	123.0 (3)	O4—C5—C6	113.4 (6)
C27A—C26A—C25A	119.4 (4)	C5—C6—H6A	109.5
C27A—C26A—H26A	120.3	C5—C6—H6B	109.5
C25A—C26A—H26A	120.3	H6A—C6—H6B	109.5
C26A—C27A—C28A	122.5 (4)	C5—C6—H6C	109.5
C26A—C27A—Br1A	119.4 (3)	H6A—C6—H6C	109.5
C28A—C27A—Br1A	118.1 (3)	H6B—C6—H6C	109.5
C29A—C28A—C27A	118.8 (3)	O4—C7—C8	108.3 (7)
C29A—C28A—H28A	120.6	O4—C7—H7A	110.0
C27A—C28A—H28A	120.6	C8—C7—H7A	110.0
C28A—C29A—C30A	120.8 (4)	O4—C7—H7B	110.0
C28A—C29A—H29A	119.6	C8—C7—H7B	110.0
C30A—C29A—H29A	119.6	H7A—C7—H7B	108.4
N2A—C30A—C29A	118.8 (3)	C7—C8—H8D	109.5
N2A—C30A—C25A	122.0 (3)	C7—C8—H8E	109.5
C29A—C30A—C25A	119.2 (3)	H8D—C8—H8E	109.5
N2A—C31A—O2A	120.1 (3)	C7—C8—H8F	109.5
N2A—C31A—C23A	125.2 (3)	H8D—C8—H8F	109.5
O2A—C31A—C23A	114.7 (3)	H8E—C8—H8F	109.5
O2A—C32A—H32A	109.5	C5E—O4E—C7E	114.9 (6)
O2A—C32A—H32B	109.5	O3E—C5E—O4E	117.6 (7)
H32A—C32A—H32B	109.5	O3E—C5E—C6E	128.8 (7)
O2A—C32A—H32C	109.5	O4E—C5E—C6E	113.4 (6)
H32A—C32A—H32C	109.5	C5E—C6E—H6EA	109.5
H32B—C32A—H32C	109.5	C5E—C6E—H6EB	109.5
O3A—C33A—O4A	122.3 (4)	H6EA—C6E—H6EB	109.5
O3A—C33A—C34A	117.2 (3)	C5E—C6E—H6EC	109.5
O4A—C33A—C34A	120.5 (4)	H6EA—C6E—H6EC	109.5
C35A—C34A—C33A	129.4 (3)	H6EB—C6E—H6EC	109.5
C35A—C34A—H34A	115.3	O4E—C7E—C8E	108.3 (7)
C33A—C34A—H34A	115.3	O4E—C7E—H7EA	110.0
C34A—C35A—C36A	131.4 (3)	C8E—C7E—H7EA	110.0
C34A—C35A—H35A	114.3	O4E—C7E—H7EB	110.0
C36A—C35A—H35A	114.3	C8E—C7E—H7EB	110.0
O6A—C36A—O5A	122.3 (4)	H7EA—C7E—H7EB	108.4
O6A—C36A—C35A	118.1 (4)	C7E—C8E—H8EA	109.5
O5A—C36A—C35A	119.5 (3)	C7E—C8E—H8EB	109.5
C2B—O1B—H1BB	115 (4)	H8EA—C8E—H8EB	109.5
C31B—O2B—C32B	117.4 (3)	C7E—C8E—H8EC	109.5
C33B—O4B—H5B	103 (3)	H8EA—C8E—H8EC	109.5
C36B—O5B—H5B	101 (3)	H8EB—C8E—H8EC	109.5
C6B—N1B—C5B	109.7 (3)	C5F—O4F—C7F	115.0 (6)
C6B—N1B—C4B	111.0 (3)	O3F—C5F—O4F	117.7 (7)
C5B—N1B—C4B	113.1 (3)	O3F—C5F—C6F	128.8 (7)
C6B—N1B—H1B	107.6	O4F—C5F—C6F	113.4 (6)
C5B—N1B—H1B	107.6	C5F—C6F—H6FA	109.5
C4B—N1B—H1B	107.6	C5F—C6F—H6FB	109.5
C31B—N2B—C30B	117.0 (3)	H6FA—C6F—H6FB	109.5
C17B—C1B—C23B	113.4 (8)	C5F—C6F—H6FC	109.5
C23B—C1B—C17D	111.0 (9)	H6FA—C6F—H6FC	109.5
C17B—C1B—C2B	118.5 (7)	H6FB—C6F—H6FC	109.5
C23B—C1B—C2B	110.7 (3)	O4F—C7F—C8F	108.3 (7)
C17D—C1B—C2B	109.3 (7)	O4F—C7F—H7FA	110.0
C17B—C1B—H1BA	104.2	C8F—C7F—H7FA	110.0
C23B—C1B—H1BA	104.2	O4F—C7F—H7FB	110.0
C2B—C1B—H1BA	104.2	C8F—C7F—H7FB	110.0
O1B—C2B—C7B	106.8 (3)	H7FA—C7F—H7FB	108.4
O1B—C2B—C3B	108.2 (3)	C7F—C8F—H8FA	109.5
C7B—C2B—C3B	112.5 (3)	C7F—C8F—H8FB	109.5
O1B—C2B—C1B	109.1 (3)	H8FA—C8F—H8FB	109.5
C7B—C2B—C1B	111.2 (3)	C7F—C8F—H8FC	109.5
C3B—C2B—C1B	108.9 (3)	H8FA—C8F—H8FC	109.5
C4B—C3B—C2B	113.7 (3)	H8FB—C8F—H8FC	109.5
C4B—C3B—H3BA	108.8	C5G—O4G—C7G	114.9 (6)
C2B—C3B—H3BA	108.8	O3G—C5G—O4G	117.6 (7)
C4B—C3B—H3BB	108.8	O3G—C5G—C6G	128.9 (7)
C2B—C3B—H3BB	108.8	O4G—C5G—C6G	113.4 (6)
H3BA—C3B—H3BB	107.7	C5G—C6G—H6GA	109.5
N1B—C4B—C3B	110.4 (3)	C5G—C6G—H6GB	109.5
N1B—C4B—H4BA	109.6	H6GA—C6G—H6GB	109.5
C3B—C4B—H4BA	109.6	C5G—C6G—H6GC	109.5
N1B—C4B—H4BB	109.6	H6GA—C6G—H6GC	109.5
C3B—C4B—H4BB	109.6	H6GB—C6G—H6GC	109.5
H4BA—C4B—H4BB	108.1	O4G—C7G—C8G	108.3 (7)
N1B—C5B—H5BA	109.5	O4G—C7G—H7GA	110.0
N1B—C5B—H5BB	109.5	C8G—C7G—H7GA	110.0
H5BA—C5B—H5BB	109.5	O4G—C7G—H7GB	110.0
N1B—C5B—H5BC	109.5	C8G—C7G—H7GB	110.0
H5BA—C5B—H5BC	109.5	H7GA—C7G—H7GB	108.4
H5BB—C5B—H5BC	109.5	C7G—C8G—H8GA	109.5
N1B—C6B—H6BA	109.5	C7G—C8G—H8GB	109.5
N1B—C6B—H6BB	109.5	H8GA—C8G—H8GB	109.5
H6BA—C6B—H6BB	109.5	C7G—C8G—H8GC	109.5
N1B—C6B—H6BC	109.5	H8GA—C8G—H8GC	109.5
H6BA—C6B—H6BC	109.5	H8GB—C8G—H8GC	109.5
H6BB—C6B—H6BC	109.5	C5H—O4H—C7H	114.9 (6)
C8B—C7B—C16B	118.1 (3)	O3H—C5H—O4H	117.6 (7)
C8B—C7B—C2B	118.3 (3)	O3H—C5H—C6H	128.9 (7)
C16B—C7B—C2B	123.5 (3)	O4H—C5H—C6H	113.4 (6)
C7B—C8B—C9B	122.6 (4)	C5H—C6H—H6HA	109.5
C7B—C8B—H8B	118.7	C5H—C6H—H6HB	109.5
C9B—C8B—H8B	118.7	H6HA—C6H—H6HB	109.5
C10B—C9B—C8B	120.4 (4)	C5H—C6H—H6HC	109.5
C10B—C9B—H9B	119.8	H6HA—C6H—H6HC	109.5
C8B—C9B—H9B	119.8	H6HB—C6H—H6HC	109.5
C9B—C10B—C11B	120.5 (3)	O4H—C7H—C8H	108.4 (7)
C9B—C10B—H10B	119.7	O4H—C7H—H7HA	110.0
C11B—C10B—H10B	119.7	C8H—C7H—H7HA	110.0
C12B—C11B—C10B	120.3 (4)	O4H—C7H—H7HB	110.0
C12B—C11B—C16B	120.3 (4)	C8H—C7H—H7HB	110.0
C10B—C11B—C16B	119.3 (4)	H7HA—C7H—H7HB	108.4
C13B—C12B—C11B	122.1 (4)	C7H—C8H—H8HA	109.5
C13B—C12B—H12B	118.9	C7H—C8H—H8HB	109.5
C11B—C12B—H12B	118.9	H8HA—C8H—H8HB	109.5
C12B—C13B—C14B	119.1 (4)	C7H—C8H—H8HC	109.5
C12B—C13B—H13B	120.5	H8HA—C8H—H8HC	109.5
C14B—C13B—H13B	120.5	H8HB—C8H—H8HC	109.5
C15B—C14B—C13B	120.8 (5)		
			
C17A—C1A—C2A—O1A	69.3 (4)	C23B—C1B—C2B—C7B	−173.4 (3)
C23A—C1A—C2A—O1A	−58.3 (3)	C17D—C1B—C2B—C7B	−50.8 (8)
C17A—C1A—C2A—C7C	−33.9 (13)	C17B—C1B—C2B—C3B	−164.4 (7)
C23A—C1A—C2A—C7C	−161.5 (13)	C23B—C1B—C2B—C3B	62.1 (4)
C17A—C1A—C2A—C3A	−172.0 (3)	C17D—C1B—C2B—C3B	−175.3 (8)
C23A—C1A—C2A—C3A	60.3 (4)	O1B—C2B—C3B—C4B	−65.7 (4)
C17A—C1A—C2A—C7A	−50.6 (4)	C7B—C2B—C3B—C4B	52.1 (4)
C23A—C1A—C2A—C7A	−178.2 (3)	C1B—C2B—C3B—C4B	175.9 (3)
O1A—C2A—C3A—C4A	−64.2 (4)	C6B—N1B—C4B—C3B	164.1 (3)
C7C—C2A—C3A—C4A	38.9 (14)	C5B—N1B—C4B—C3B	−72.0 (4)
C7A—C2A—C3A—C4A	55.5 (4)	C2B—C3B—C4B—N1B	174.6 (3)
C1A—C2A—C3A—C4A	176.4 (3)	O1B—C2B—C7B—C8B	1.3 (4)
C6A—N1A—C4A—C3A	163.4 (3)	C3B—C2B—C7B—C8B	−117.3 (4)
C5A—N1A—C4A—C3A	−72.8 (4)	C1B—C2B—C7B—C8B	120.2 (3)
C2A—C3A—C4A—N1A	173.9 (3)	O1B—C2B—C7B—C16B	−178.4 (3)
O1A—C2A—C7A—C8A	1.4 (5)	C3B—C2B—C7B—C16B	63.0 (4)
C3A—C2A—C7A—C8A	−117.7 (4)	C1B—C2B—C7B—C16B	−59.5 (4)
C1A—C2A—C7A—C8A	121.5 (4)	C16B—C7B—C8B—C9B	−1.1 (6)
O1A—C2A—C7A—C16A	−178.5 (4)	C2B—C7B—C8B—C9B	179.2 (3)
C3A—C2A—C7A—C16A	62.4 (5)	C7B—C8B—C9B—C10B	1.6 (6)
C1A—C2A—C7A—C16A	−58.4 (5)	C8B—C9B—C10B—C11B	−0.7 (6)
C16A—C7A—C8A—C9A	1.0 (7)	C9B—C10B—C11B—C12B	−178.4 (4)
C2A—C7A—C8A—C9A	−178.9 (4)	C9B—C10B—C11B—C16B	−0.6 (6)
C7A—C8A—C9A—C10A	−0.2 (7)	C10B—C11B—C12B—C13B	178.8 (5)
C8A—C9A—C10A—C11A	0.2 (8)	C16B—C11B—C12B—C13B	1.0 (7)
C9A—C10A—C11A—C16A	−1.0 (9)	C11B—C12B—C13B—C14B	−1.4 (8)
C9A—C10A—C11A—C12A	−177.9 (7)	C12B—C13B—C14B—C15B	1.6 (8)
C10A—C11A—C12A—C13A	178.1 (9)	C13B—C14B—C15B—C16B	−1.4 (7)
C16A—C11A—C12A—C13A	1.2 (14)	C14B—C15B—C16B—C7B	−178.6 (4)
C11A—C12A—C13A—C14A	−0.5 (16)	C14B—C15B—C16B—C11B	0.9 (6)
C12A—C13A—C14A—C15A	−0.6 (15)	C8B—C7B—C16B—C15B	179.3 (4)
C13A—C14A—C15A—C16A	1.0 (12)	C2B—C7B—C16B—C15B	−1.0 (6)
C14A—C15A—C16A—C11A	−0.3 (10)	C8B—C7B—C16B—C11B	−0.2 (5)
C14A—C15A—C16A—C7A	−179.7 (6)	C2B—C7B—C16B—C11B	179.4 (3)
C10A—C11A—C16A—C15A	−177.6 (6)	C12B—C11B—C16B—C15B	−0.7 (6)
C12A—C11A—C16A—C15A	−0.8 (10)	C10B—C11B—C16B—C15B	−178.5 (4)
C10A—C11A—C16A—C7A	1.8 (9)	C12B—C11B—C16B—C7B	178.9 (4)
C12A—C11A—C16A—C7A	178.7 (7)	C10B—C11B—C16B—C7B	1.1 (5)
C8A—C7A—C16A—C15A	177.7 (5)	C23B—C1B—C17B—C22B	−99.8 (14)
C2A—C7A—C16A—C15A	−2.5 (8)	C2B—C1B—C17B—C22B	127.9 (12)
C8A—C7A—C16A—C11A	−1.7 (7)	C23B—C1B—C17B—C18B	77.7 (15)
C2A—C7A—C16A—C11A	178.2 (5)	C2B—C1B—C17B—C18B	−54.6 (16)
O1A—C2A—C7C—C8C	−4.6 (19)	C22B—C17B—C18B—C19B	0 (2)
C3A—C2A—C7C—C8C	−116.8 (16)	C1B—C17B—C18B—C19B	−178.1 (13)
C1A—C2A—C7C—C8C	108.8 (17)	C17B—C18B—C19B—C20B	1.7 (19)
O1A—C2A—C7C—C16C	179 (3)	C18B—C19B—C20B—C21B	−1.2 (17)
C3A—C2A—C7C—C16C	67 (3)	C19B—C20B—C21B—C22B	−0.6 (16)
C1A—C2A—C7C—C16C	−68 (3)	C18B—C17B—C22B—C21B	−1 (2)
C16C—C7C—C8C—C9C	−4 (2)	C1B—C17B—C22B—C21B	176.3 (13)
C2A—C7C—C8C—C9C	180 (3)	C20B—C21B—C22B—C17B	1.8 (17)
C7C—C8C—C9C—C10C	2 (2)	C23B—C1B—C17D—C22D	−103.6 (14)
C8C—C9C—C10C—C11C	1 (4)	C2B—C1B—C17D—C22D	134.0 (13)
C9C—C10C—C11C—C12C	−177 (3)	C23B—C1B—C17D—C18D	78.0 (18)
C9C—C10C—C11C—C16C	−2 (6)	C2B—C1B—C17D—C18D	−44.4 (19)
C10C—C11C—C12C—C13C	166 (4)	C22D—C17D—C18D—C19D	5 (2)
C16C—C11C—C12C—C13C	−10 (6)	C1B—C17D—C18D—C19D	−177.1 (15)
C11C—C12C—C13C—C14C	15 (7)	C17D—C18D—C19D—C20D	−3 (2)
C12C—C13C—C14C—C15C	−13 (7)	C18D—C19D—C20D—C21D	−2 (2)
C13C—C14C—C15C—C16C	6 (8)	C19D—C20D—C21D—C22D	5 (2)
C14C—C15C—C16C—C7C	−173 (4)	C18D—C17D—C22D—C21D	−2 (3)
C14C—C15C—C16C—C11C	0 (7)	C1B—C17D—C22D—C21D	179.6 (15)
C8C—C7C—C16C—C15C	176 (4)	C20D—C21D—C22D—C17D	−3 (2)
C2A—C7C—C16C—C15C	−7 (6)	C17B—C1B—C23B—C24B	−47.9 (7)
C8C—C7C—C16C—C11C	3 (5)	C17D—C1B—C23B—C24B	−33.5 (8)
C2A—C7C—C16C—C11C	180 (3)	C2B—C1B—C23B—C24B	88.1 (4)
C12C—C11C—C16C—C15C	2 (6)	C17B—C1B—C23B—C31B	134.3 (6)
C10C—C11C—C16C—C15C	−174 (4)	C17D—C1B—C23B—C31B	148.7 (7)
C12C—C11C—C16C—C7C	176 (4)	C2B—C1B—C23B—C31B	−89.7 (4)
C10C—C11C—C16C—C7C	0 (6)	C31B—C23B—C24B—C25B	2.1 (5)
C23A—C1A—C17A—C22A	−96.5 (4)	C1B—C23B—C24B—C25B	−175.8 (3)
C2A—C1A—C17A—C22A	136.9 (4)	C23B—C24B—C25B—C30B	2.9 (5)
C23A—C1A—C17A—C18A	81.6 (4)	C23B—C24B—C25B—C26B	−178.3 (4)
C2A—C1A—C17A—C18A	−45.1 (5)	C30B—C25B—C26B—C27B	2.3 (6)
C22A—C17A—C18A—C19A	0.2 (6)	C24B—C25B—C26B—C27B	−176.5 (4)
C1A—C17A—C18A—C19A	−177.8 (4)	C25B—C26B—C27B—C28B	−0.3 (7)
C17A—C18A—C19A—C20A	0.8 (7)	C25B—C26B—C27B—Br1B	−179.1 (3)
C18A—C19A—C20A—C21A	−1.2 (7)	C26B—C27B—C28B—C29B	−1.0 (7)
C19A—C20A—C21A—C22A	0.6 (8)	Br1B—C27B—C28B—C29B	177.7 (3)
C18A—C17A—C22A—C21A	−0.8 (6)	C27B—C28B—C29B—C30B	0.4 (6)
C1A—C17A—C22A—C21A	177.4 (4)	C31B—N2B—C30B—C25B	0.7 (5)
C20A—C21A—C22A—C17A	0.4 (8)	C31B—N2B—C30B—C29B	−179.8 (3)
C17A—C1A—C23A—C24A	−37.5 (4)	C26B—C25B—C30B—N2B	176.6 (3)
C2A—C1A—C23A—C24A	91.1 (4)	C24B—C25B—C30B—N2B	−4.5 (5)
C17A—C1A—C23A—C31A	142.3 (3)	C26B—C25B—C30B—C29B	−2.8 (5)
C2A—C1A—C23A—C31A	−89.1 (3)	C24B—C25B—C30B—C29B	176.0 (3)
C31A—C23A—C24A—C25A	4.3 (4)	C28B—C29B—C30B—N2B	−178.0 (3)
C1A—C23A—C24A—C25A	−175.9 (3)	C28B—C29B—C30B—C25B	1.5 (5)
C23A—C24A—C25A—C30A	2.2 (5)	C30B—N2B—C31B—O2B	−174.3 (3)
C23A—C24A—C25A—C26A	−178.8 (3)	C30B—N2B—C31B—C23B	5.1 (5)
C30A—C25A—C26A—C27A	2.4 (5)	C32B—O2B—C31B—N2B	−0.1 (5)
C24A—C25A—C26A—C27A	−176.6 (3)	C32B—O2B—C31B—C23B	−179.6 (3)
C25A—C26A—C27A—C28A	−0.2 (6)	C24B—C23B—C31B—N2B	−6.5 (5)
C25A—C26A—C27A—Br1A	179.5 (3)	C1B—C23B—C31B—N2B	171.5 (3)
C26A—C27A—C28A—C29A	−1.0 (6)	C24B—C23B—C31B—O2B	172.9 (3)
Br1A—C27A—C28A—C29A	179.2 (3)	C1B—C23B—C31B—O2B	−9.1 (4)
C27A—C28A—C29A—C30A	0.1 (5)	O3B—C33B—C34B—C35B	179.8 (5)
C31A—N2A—C30A—C29A	−179.8 (3)	O4B—C33B—C34B—C35B	1.0 (8)
C31A—N2A—C30A—C25A	1.5 (4)	C33B—C34B—C35B—C36B	−0.9 (8)
C28A—C29A—C30A—N2A	−176.7 (3)	C34B—C35B—C36B—O6B	−175.1 (5)
C28A—C29A—C30A—C25A	2.0 (5)	C34B—C35B—C36B—O5B	4.1 (7)
C26A—C25A—C30A—N2A	175.4 (3)	C3—O2—C1—O1	3.9 (11)
C24A—C25A—C30A—N2A	−5.5 (5)	C3—O2—C1—C2	−177.9 (8)
C26A—C25A—C30A—C29A	−3.3 (5)	C1—O2—C3—C4	−172.1 (8)
C24A—C25A—C30A—C29A	175.8 (3)	C3E—O2E—C1E—O1E	−11 (3)
C30A—N2A—C31A—O2A	−173.3 (3)	C3E—O2E—C1E—C2E	164 (3)
C30A—N2A—C31A—C23A	5.9 (5)	C1E—O2E—C3E—C4E	81 (3)
C32A—O2A—C31A—N2A	1.0 (5)	C7—O4—C5—O3	−9 (3)
C32A—O2A—C31A—C23A	−178.3 (3)	C7—O4—C5—C6	173.5 (18)
C24A—C23A—C31A—N2A	−8.9 (5)	C5—O4—C7—C8	−80 (3)
C1A—C23A—C31A—N2A	171.2 (3)	C7E—O4E—C5E—O3E	12 (3)
C24A—C23A—C31A—O2A	170.4 (3)	C7E—O4E—C5E—C6E	−172.6 (19)
C1A—C23A—C31A—O2A	−9.5 (4)	C5E—O4E—C7E—C8E	85 (2)
O3A—C33A—C34A—C35A	−177.0 (4)	C7F—O4F—C5F—O3F	3 (3)
O4A—C33A—C34A—C35A	4.0 (8)	C7F—O4F—C5F—C6F	180 (2)
C33A—C34A—C35A—C36A	0.3 (8)	C5F—O4F—C7F—C8F	−133 (3)
C34A—C35A—C36A—O6A	−178.6 (5)	C7G—O4G—C5G—O3G	66 (4)
C34A—C35A—C36A—O5A	0.8 (8)	C7G—O4G—C5G—C6G	−117 (4)
C17B—C1B—C2B—O1B	77.7 (7)	C5G—O4G—C7G—C8G	−156 (3)
C23B—C1B—C2B—O1B	−55.8 (3)	C7H—O4H—C5H—O3H	2 (5)
C17D—C1B—C2B—O1B	66.8 (8)	C7H—O4H—C5H—C6H	−176 (4)
C17B—C1B—C2B—C7B	−39.9 (8)	C5H—O4H—C7H—C8H	−175 (4)

### [4-(6-bromo-2-methoxyquinolin-3-yl)-3-hydroxy-3-(naphthalen-1-yl)-4-phenylbutyl]dimethylazanium 3-carboxyprop-2-enoate ethyl acetate 0.821-solvate (ethyl_acetate) Hydrogen-bond geometry (Å, º)

**Table:**

*D*—H···*A*	*D*—H	H···*A*	*D*···*A*	*D*—H···*A*
O1*A*—H1*AB*···O6*B*^i^	0.84 (6)	1.98 (6)	2.791 (4)	160 (6)
O5*A*—H5*A*···O4*A*	1.11 (10)	1.33 (10)	2.426 (5)	171 (9)
N1*A*—H1*A*···O3*A*	1.00	1.71	2.707 (4)	175
N1*A*—H1*A*···O4*A*	1.00	2.54	3.211 (4)	125
O1*B*—H1*BB*···O6*A*	0.92 (6)	1.91 (6)	2.812 (4)	168 (6)
O5*B*—H5*B*···O4*B*	1.17 (8)	1.29 (8)	2.429 (4)	164 (6)
N1*B*—H1*B*···O3*B*	1.00	1.70	2.701 (4)	174
N1*B*—H1*B*···O4*B*	1.00	2.52	3.204 (4)	125
C3*A*—H3*AA*···O6*B*^i^	0.99	2.56	3.251 (4)	127
C5*A*—H5*AC*···O3^ii^	0.98	2.60	3.391 (15)	138
C6*A*—H6*AA*···O4*B*^iii^	0.98	2.59	3.218 (5)	122
C32*A*—H32*A*···O1^iv^	0.98	2.59	3.220 (7)	122
C1*B*—H1*BA*···O2*B*	1.00	2.26	2.780 (4)	111
C3*B*—H3*BA*···O6*A*	0.99	2.58	3.284 (4)	128
C6*B*—H6*BA*···O4*A*^v^	0.98	2.58	3.190 (5)	121
C2*E*—H2*EB*···O3*A*^vi^	0.98	2.34	2.96 (2)	120
C4*E*—H4*EB*···O1*E*	0.98	2.37	2.91 (5)	114
C7—H7*A*···O4*A*^iii^	0.99	2.29	3.068 (15)	134
C8*E*—H8*EC*···O5*A*^iii^	0.98	2.65	3.57 (3)	157
C8*G*—H8*GB*···O1*A*^v^	0.98	2.05	2.90 (5)	145

Symmetry codes: (i) *x*−1, *y*−1, *z*; (ii) *x*−1, *y*, *z*; (iii) −*x*+1, *y*−1/2, −*z*; (iv) −*x*+1, *y*+1/2, −*z*+1; (v) −*x*+1, *y*+1/2, −*z*; (vi) −*x*+1, *y*−1/2, −*z*+1.

### [4-(6-bromo-2-methoxyquinolin-3-yl)-3-hydroxy-3-(naphthalen-1-yl)-4-phenylbutyl]dimethylazanium 3-carboxyprop-2-enoate–hexane–acetone (1/0.248/1) (acetone_hexane) Crystal data

**Table:**

2C_32_H_32_BrN_2_O_2_^+^·2C_4_H_3_O_4_^−^·0.495C_6_H_14_·2.01C_3_H_6_O	*F*(000) = 1570.1
*M**_r_* = 1502.51	*D*_x_ = 1.336 Mg m^−^^3^
Monoclinic, *C*2	Mo *K*α radiation, λ = 0.71073 Å
*a* = 16.0678 (9) Å	Cell parameters from 9698 reflections
*b* = 13.6440 (8) Å	θ = 2.5–31.7°
*c* = 17.8720 (8) Å	µ = 1.15 mm^−^^1^
β = 107.6347 (18)°	*T* = 150 K
*V* = 3733.9 (3) Å^3^	Block, colourless
*Z* = 2	0.48 × 0.35 × 0.21 mm

### [4-(6-bromo-2-methoxyquinolin-3-yl)-3-hydroxy-3-(naphthalen-1-yl)-4-phenylbutyl]dimethylazanium 3-carboxyprop-2-enoate–hexane–acetone (1/0.248/1) (acetone_hexane) Data collection

**Table:**

Bruker AXS D8 Quest with PhotonII CPAD diffractometer	14074 independent reflections
Radiation source: fine focus sealed tube X-ray source	9700 reflections with *I* > 2σ(*I*)
Triumph curved graphite crystal monochromator	*R*_int_ = 0.059
Detector resolution: 7.4074 pixels mm^-1^	θ_max_ = 33.1°, θ_min_ = 2.4°
ω and phi scans	*h* = −24→24
Absorption correction: multi-scan (SADABS; Krause *et al.*, 2015)	*k* = −20→20
*T*_min_ = 0.439, *T*_max_ = 0.498	*l* = −27→22
73087 measured reflections	

### [4-(6-bromo-2-methoxyquinolin-3-yl)-3-hydroxy-3-(naphthalen-1-yl)-4-phenylbutyl]dimethylazanium 3-carboxyprop-2-enoate–hexane–acetone (1/0.248/1) (acetone_hexane) Refinement

**Table:**

Refinement on *F*^2^	Hydrogen site location: mixed
Least-squares matrix: full	H atoms treated by a mixture of independent and constrained refinement
*R*[*F*^2^ > 2σ(*F*^2^)] = 0.047	*w* = 1/[σ^2^(*F*_o_^2^) + (0.0357*P*)^2^ + 1.6623*P*] where *P* = (*F*_o_^2^ + 2*F*_c_^2^)/3
*wR*(*F*^2^) = 0.126	(Δ/σ)_max_ < 0.001
*S* = 1.03	Δρ_max_ = 0.61 e Å^−^^3^
14074 reflections	Δρ_min_ = −0.62 e Å^−^^3^
558 parameters	Extinction correction: SHELXL2018/3 (Sheldrick, 2015b), Fc^*^=kFc[1+0.001xFc^2^λ^3^/sin(2θ)]^-1/4^
407 restraints	Extinction coefficient: 0.0034 (10)
Primary atom site location: isomorphous structure methods	Absolute structure: Flack *x* determined using 3753 quotients [(*I*^+^)-(*I*^-^)]/[(*I*^+^)+(*I*^-^)] (Parsons *et al.*, 2013)
Secondary atom site location: difference Fourier map	Absolute structure parameter: 0.006 (3)

### [4-(6-bromo-2-methoxyquinolin-3-yl)-3-hydroxy-3-(naphthalen-1-yl)-4-phenylbutyl]dimethylazanium 3-carboxyprop-2-enoate–hexane–acetone (1/0.248/1) (acetone_hexane) Special details

**Table:**

Geometry. All esds (except the esd in the dihedral angle between two l.s. planes) are estimated using the full covariance matrix. The cell esds are taken into account individually in the estimation of esds in distances, angles and torsion angles; correlations between esds in cell parameters are only used when they are defined by crystal symmetry. An approximate (isotropic) treatment of cell esds is used for estimating esds involving l.s. planes.
Refinement. The structure was solved by isomorphous replacement from its hemi- hydrate analogue (structure code RPI_46_1_2). Two symmetry equivalent acetone molecules are disordered with a hexane molecule located on a two-fold axis. Another acetone molecule is located on and disordered around a two-fold axis and additionally disordered by a slight tilt of the molecule. All acetone moieties were restrained to have similar geometries and to be close to planar. The two C-C bond distances within all acetone were restrained to be similar. C-C bond distances of the hexane molecule were restrained to target values (1.55 (1) Angstrom) and hexane C-C-C angles were restrained to be similar. Uij components of ADPs for disordered atoms closer to each other than 2.0 Angstrom were restrained to be similar. Subject to these conditions the occupancy rates refined to 0.505 (9) and 0.495 (9) for the acetone / hexane disorder, and two times 0.230 (11) and two times 0.270 (11) for the disordered acetone. Acetone and hexane molecules are located in infinite channels and slowly vacate the crystal lattice. Crystals become milky overnight when taken out of mother liquor solution and left to dry in air, but retain crystallinity. Data collection reveals a solvent free structure (see dataset 91_2_dry).

### [4-(6-bromo-2-methoxyquinolin-3-yl)-3-hydroxy-3-(naphthalen-1-yl)-4-phenylbutyl]dimethylazanium 3-carboxyprop-2-enoate–hexane–acetone (1/0.248/1) (acetone_hexane) Fractional atomic coordinates and isotropic or equivalent isotropic displacement parameters (Å^2^)

**Table:**

	*x*	*y*	*z*	*U*_iso_*/*U*_eq_	Occ. (<1)
Br1	0.28108 (4)	0.03150 (4)	0.45830 (4)	0.1050 (2)	
O1	0.48565 (12)	0.26573 (13)	0.16549 (10)	0.0360 (4)	
H1O	0.440644	0.279937	0.177824	0.043*	
O2	0.56438 (12)	0.47869 (14)	0.36251 (10)	0.0366 (4)	
O3	0.50234 (13)	0.68019 (15)	0.21207 (12)	0.0437 (4)	
O4	0.57291 (17)	0.6900 (3)	0.12449 (13)	0.0702 (8)	
O5	0.70949 (16)	0.7561 (2)	0.11557 (13)	0.0668 (8)	
H5	0.642 (3)	0.727 (4)	0.122 (3)	0.080*	
O6	0.82960 (13)	0.81698 (18)	0.19657 (14)	0.0513 (5)	
N1	0.40093 (12)	0.55897 (14)	0.10162 (11)	0.0292 (4)	
H1	0.437687	0.607063	0.139756	0.035*	
N2	0.45948 (13)	0.40362 (16)	0.40492 (11)	0.0328 (4)	
C1	0.60196 (15)	0.31733 (18)	0.28231 (13)	0.0309 (4)	
H1A	0.640880	0.374982	0.301923	0.037*	
C2	0.54990 (15)	0.33968 (17)	0.19372 (12)	0.0293 (4)	
C3	0.50225 (15)	0.43881 (17)	0.18800 (12)	0.0282 (4)	
H3A	0.459176	0.434483	0.217451	0.034*	
H3B	0.545234	0.490092	0.213099	0.034*	
C4	0.45543 (16)	0.46892 (18)	0.10393 (13)	0.0312 (4)	
H4A	0.498951	0.482057	0.076072	0.037*	
H4B	0.417505	0.414569	0.076535	0.037*	
C5	0.32133 (17)	0.5395 (2)	0.12599 (18)	0.0437 (6)	
H5A	0.284267	0.491520	0.090222	0.066*	
H5B	0.288771	0.600620	0.124098	0.066*	
H5C	0.338747	0.513454	0.179618	0.066*	
C6	0.37641 (18)	0.6054 (2)	0.02278 (15)	0.0388 (5)	
H6A	0.345648	0.557539	−0.016892	0.058*	
H6B	0.429230	0.627598	0.011272	0.058*	
H6C	0.338160	0.661574	0.021921	0.058*	
C7	0.61170 (16)	0.33633 (18)	0.14255 (13)	0.0334 (5)	
C8	0.5954 (2)	0.2673 (2)	0.08362 (14)	0.0426 (6)	
H8	0.546889	0.224460	0.075988	0.051*	
C9	0.6497 (2)	0.2587 (3)	0.03370 (15)	0.0533 (8)	
H9	0.636836	0.210335	−0.006425	0.064*	
C10	0.7186 (2)	0.3182 (3)	0.04292 (17)	0.0529 (8)	
H10	0.754151	0.311643	0.009299	0.063*	
C11	0.7386 (2)	0.3900 (3)	0.10180 (17)	0.0477 (7)	
C12	0.8104 (2)	0.4549 (3)	0.1104 (2)	0.0638 (10)	
H12	0.845434	0.447108	0.076433	0.077*	
C13	0.8306 (2)	0.5266 (4)	0.1644 (2)	0.0657 (9)	
H13	0.878384	0.569256	0.168070	0.079*	
C14	0.7799 (2)	0.5372 (3)	0.2152 (2)	0.0537 (7)	
H14	0.794272	0.587019	0.254109	0.064*	
C15	0.70973 (18)	0.4770 (2)	0.20992 (16)	0.0415 (6)	
H15	0.676695	0.486501	0.245324	0.050*	
C16	0.68507 (16)	0.4007 (2)	0.15300 (14)	0.0360 (5)	
C17	0.66129 (17)	0.2277 (2)	0.29397 (14)	0.0374 (5)	
C18	0.6378 (2)	0.1398 (2)	0.2548 (2)	0.0508 (7)	
H18	0.581932	0.133510	0.216972	0.061*	
C19	0.6951 (3)	0.0602 (3)	0.2700 (2)	0.0641 (9)	
H19	0.678486	0.000948	0.241496	0.077*	
C20	0.7750 (3)	0.0670 (3)	0.3256 (2)	0.0708 (11)	
H20	0.813448	0.012276	0.337027	0.085*	
C21	0.7987 (3)	0.1538 (4)	0.3645 (3)	0.0800 (13)	
H21	0.854203	0.159268	0.403026	0.096*	
C22	0.7429 (2)	0.2343 (3)	0.34874 (19)	0.0566 (8)	
H22	0.761007	0.294280	0.375823	0.068*	
C23	0.54033 (15)	0.31153 (18)	0.33221 (12)	0.0298 (4)	
C24	0.50170 (16)	0.22692 (18)	0.34487 (13)	0.0321 (4)	
H24	0.515631	0.166907	0.324504	0.039*	
C25	0.44064 (16)	0.2275 (2)	0.38831 (13)	0.0341 (5)	
C26	0.39966 (19)	0.1409 (2)	0.40278 (17)	0.0440 (6)	
H26	0.414743	0.079158	0.385947	0.053*	
C27	0.3380 (2)	0.1469 (3)	0.4413 (2)	0.0521 (7)	
C28	0.31484 (19)	0.2374 (3)	0.46714 (17)	0.0495 (7)	
H28	0.271320	0.240153	0.493110	0.059*	
C29	0.35541 (16)	0.3215 (2)	0.45464 (14)	0.0404 (6)	
H29	0.340300	0.382411	0.472727	0.048*	
C30	0.41953 (15)	0.3185 (2)	0.41512 (12)	0.0326 (5)	
C31	0.51833 (15)	0.39829 (18)	0.36816 (12)	0.0302 (4)	
C32	0.5439 (2)	0.5672 (2)	0.39697 (19)	0.0495 (7)	
H32A	0.484998	0.589046	0.367337	0.074*	
H32B	0.586226	0.618203	0.395465	0.074*	
H32C	0.546336	0.554523	0.451582	0.074*	
C33	0.56682 (18)	0.7043 (2)	0.19291 (15)	0.0391 (5)	
C34	0.64121 (18)	0.7502 (2)	0.25429 (14)	0.0388 (5)	
H34	0.632657	0.756615	0.304375	0.047*	
C35	0.71764 (18)	0.7835 (2)	0.25022 (15)	0.0392 (5)	
H35	0.754338	0.810422	0.297846	0.047*	
C36	0.75529 (18)	0.7859 (2)	0.18370 (16)	0.0398 (5)	
O7	0.5989 (5)	0.8015 (5)	0.4370 (5)	0.097 (3)	0.505 (9)
C37	0.5409 (12)	0.8497 (16)	0.3963 (12)	0.119 (4)	0.505 (9)
C38	0.4480 (8)	0.8322 (11)	0.3707 (11)	0.128 (4)	0.505 (9)
H38A	0.429939	0.809616	0.316077	0.193*	0.505 (9)
H38B	0.417134	0.893012	0.374591	0.193*	0.505 (9)
H38C	0.433707	0.781911	0.404054	0.193*	0.505 (9)
C39	0.5660 (10)	0.9345 (9)	0.3579 (11)	0.125 (5)	0.505 (9)
H39A	0.626967	0.927640	0.358730	0.187*	0.505 (9)
H39B	0.559451	0.994471	0.385823	0.187*	0.505 (9)
H39C	0.528346	0.938335	0.303383	0.187*	0.505 (9)
C43	0.4921 (13)	0.9070 (14)	0.3204 (10)	0.147 (6)	0.495 (9)
H43A	0.532067	0.903050	0.288555	0.220*	0.495 (9)
H43B	0.482284	0.975965	0.330675	0.220*	0.495 (9)
H43C	0.436329	0.876333	0.292035	0.220*	0.495 (9)
C44	0.5325 (15)	0.853 (2)	0.3990 (11)	0.131 (4)	0.495 (9)
H44A	0.536158	0.782358	0.388301	0.157*	0.495 (9)
H44B	0.592752	0.877463	0.423074	0.157*	0.495 (9)
C45	0.4823 (10)	0.8658 (17)	0.4569 (4)	0.151 (5)	0.495 (9)
H45A	0.450816	0.928805	0.443022	0.181*	0.495 (9)
H45B	0.436974	0.814058	0.443951	0.181*	0.495 (9)
O8A	0.4373 (18)	1.0370 (17)	−0.0198 (16)	0.134 (7)	0.230 (11)
C40A	0.489 (2)	0.9735 (18)	−0.0008 (19)	0.087 (5)	0.230 (11)
C41A	0.482 (3)	0.8753 (17)	−0.038 (2)	0.089 (6)	0.230 (11)
H41A	0.451893	0.881229	−0.094492	0.133*	0.230 (11)
H41B	0.449048	0.831225	−0.014304	0.133*	0.230 (11)
H41C	0.540733	0.848755	−0.030175	0.133*	0.230 (11)
C42A	0.563 (2)	0.979 (3)	0.071 (2)	0.103 (8)	0.230 (11)
H42A	0.615091	0.952339	0.060829	0.155*	0.230 (11)
H42B	0.550070	0.941609	0.112743	0.155*	0.230 (11)
H42C	0.573559	1.047975	0.087216	0.155*	0.230 (11)
O8B	0.4818 (16)	1.0511 (9)	0.0406 (11)	0.120 (6)	0.270 (11)
C40B	0.4889 (18)	0.9844 (15)	0.0016 (16)	0.085 (5)	0.270 (11)
C41B	0.532 (3)	0.896 (2)	0.044 (2)	0.107 (7)	0.270 (11)
H41D	0.592218	0.893131	0.043466	0.160*	0.270 (11)
H41E	0.500248	0.837423	0.018815	0.160*	0.270 (11)
H41F	0.530423	0.899033	0.098710	0.160*	0.270 (11)
C42B	0.454 (2)	0.973 (3)	−0.0838 (13)	0.092 (7)	0.270 (11)
H42D	0.496337	0.937141	−0.103146	0.138*	0.270 (11)
H42E	0.442785	1.037169	−0.108707	0.138*	0.270 (11)
H42F	0.399223	0.935305	−0.096539	0.138*	0.270 (11)

### [4-(6-bromo-2-methoxyquinolin-3-yl)-3-hydroxy-3-(naphthalen-1-yl)-4-phenylbutyl]dimethylazanium 3-carboxyprop-2-enoate–hexane–acetone (1/0.248/1) (acetone_hexane) Atomic displacement parameters (Å^2^)

**Table:**

	*U*^11^	*U*^22^	*U*^33^	*U*^12^	*U*^13^	*U*^23^
Br1	0.1091 (4)	0.0780 (3)	0.1620 (6)	−0.0348 (3)	0.0922 (4)	−0.0104 (3)
O1	0.0413 (9)	0.0297 (8)	0.0340 (8)	0.0002 (7)	0.0066 (7)	−0.0033 (7)
O2	0.0459 (10)	0.0336 (9)	0.0320 (8)	0.0020 (7)	0.0145 (7)	−0.0046 (7)
O3	0.0448 (10)	0.0444 (11)	0.0476 (10)	−0.0094 (8)	0.0225 (8)	−0.0078 (8)
O4	0.0614 (14)	0.117 (2)	0.0389 (10)	−0.0493 (15)	0.0251 (10)	−0.0328 (13)
O5	0.0608 (13)	0.105 (2)	0.0407 (10)	−0.0419 (14)	0.0249 (10)	−0.0199 (12)
O6	0.0368 (10)	0.0504 (12)	0.0654 (13)	−0.0100 (9)	0.0135 (9)	0.0034 (10)
N1	0.0278 (8)	0.0297 (9)	0.0293 (8)	0.0043 (7)	0.0073 (7)	0.0050 (7)
N2	0.0332 (10)	0.0397 (11)	0.0237 (8)	0.0079 (8)	0.0058 (7)	−0.0006 (8)
C1	0.0319 (11)	0.0335 (11)	0.0272 (9)	0.0072 (9)	0.0090 (8)	0.0021 (8)
C2	0.0347 (11)	0.0293 (10)	0.0228 (9)	0.0059 (8)	0.0068 (8)	0.0002 (8)
C3	0.0305 (10)	0.0282 (10)	0.0243 (9)	0.0062 (8)	0.0060 (8)	0.0012 (8)
C4	0.0359 (11)	0.0309 (11)	0.0263 (9)	0.0075 (9)	0.0084 (8)	0.0019 (8)
C5	0.0320 (11)	0.0484 (15)	0.0547 (15)	0.0064 (11)	0.0192 (11)	0.0153 (13)
C6	0.0430 (13)	0.0387 (13)	0.0335 (11)	0.0078 (11)	0.0098 (10)	0.0118 (10)
C7	0.0415 (12)	0.0341 (12)	0.0250 (9)	0.0138 (10)	0.0107 (9)	0.0016 (8)
C8	0.0583 (16)	0.0399 (13)	0.0279 (10)	0.0186 (12)	0.0105 (10)	−0.0007 (10)
C9	0.079 (2)	0.0529 (17)	0.0287 (11)	0.0348 (16)	0.0167 (12)	0.0011 (11)
C10	0.0640 (18)	0.0645 (19)	0.0388 (13)	0.0340 (16)	0.0285 (13)	0.0141 (13)
C11	0.0468 (14)	0.0612 (17)	0.0418 (13)	0.0246 (13)	0.0233 (11)	0.0156 (12)
C12	0.0468 (16)	0.089 (3)	0.068 (2)	0.0153 (17)	0.0352 (15)	0.020 (2)
C13	0.0440 (16)	0.085 (2)	0.076 (2)	−0.0010 (17)	0.0295 (16)	0.010 (2)
C14	0.0432 (14)	0.0616 (18)	0.0577 (17)	−0.0046 (14)	0.0175 (13)	0.0035 (15)
C15	0.0381 (13)	0.0490 (15)	0.0399 (12)	0.0044 (11)	0.0156 (10)	0.0013 (11)
C16	0.0359 (11)	0.0444 (13)	0.0295 (10)	0.0149 (10)	0.0124 (9)	0.0067 (9)
C17	0.0384 (12)	0.0451 (14)	0.0321 (10)	0.0159 (10)	0.0159 (9)	0.0099 (10)
C18	0.0550 (17)	0.0400 (14)	0.0583 (17)	0.0217 (12)	0.0185 (14)	0.0082 (13)
C19	0.081 (2)	0.0482 (18)	0.072 (2)	0.0319 (17)	0.0356 (19)	0.0179 (16)
C20	0.075 (2)	0.077 (2)	0.069 (2)	0.052 (2)	0.0355 (18)	0.0317 (19)
C21	0.055 (2)	0.111 (3)	0.067 (2)	0.049 (2)	0.0085 (17)	0.010 (2)
C22	0.0408 (14)	0.080 (2)	0.0472 (15)	0.0257 (15)	0.0108 (12)	0.0031 (15)
C23	0.0309 (10)	0.0346 (11)	0.0229 (9)	0.0086 (8)	0.0063 (8)	0.0029 (8)
C24	0.0342 (11)	0.0330 (11)	0.0285 (10)	0.0069 (9)	0.0083 (8)	0.0011 (8)
C25	0.0311 (11)	0.0431 (13)	0.0257 (9)	0.0041 (9)	0.0050 (8)	0.0019 (9)
C26	0.0408 (14)	0.0491 (15)	0.0427 (14)	−0.0008 (11)	0.0133 (11)	0.0000 (12)
C27	0.0450 (15)	0.0615 (19)	0.0522 (16)	−0.0118 (13)	0.0185 (13)	−0.0012 (14)
C28	0.0369 (13)	0.074 (2)	0.0393 (13)	−0.0030 (13)	0.0145 (11)	−0.0012 (13)
C29	0.0313 (11)	0.0598 (17)	0.0290 (10)	0.0045 (11)	0.0075 (9)	−0.0051 (11)
C30	0.0279 (10)	0.0456 (13)	0.0204 (8)	0.0058 (9)	0.0015 (7)	0.0009 (9)
C31	0.0308 (10)	0.0340 (11)	0.0225 (9)	0.0076 (8)	0.0034 (8)	0.0016 (8)
C32	0.0688 (19)	0.0355 (13)	0.0488 (15)	0.0022 (13)	0.0248 (14)	−0.0090 (12)
C33	0.0454 (13)	0.0387 (13)	0.0357 (11)	−0.0106 (10)	0.0160 (10)	−0.0075 (10)
C34	0.0463 (13)	0.0410 (13)	0.0304 (10)	−0.0052 (11)	0.0136 (10)	−0.0033 (9)
C35	0.0437 (13)	0.0387 (13)	0.0312 (10)	−0.0066 (10)	0.0054 (9)	−0.0032 (9)
C36	0.0394 (12)	0.0355 (13)	0.0438 (13)	−0.0078 (10)	0.0117 (10)	0.0004 (10)
O7	0.093 (5)	0.061 (4)	0.142 (7)	0.014 (3)	0.045 (4)	−0.007 (4)
C37	0.115 (7)	0.095 (6)	0.166 (8)	0.002 (6)	0.071 (6)	−0.022 (6)
C38	0.102 (7)	0.100 (8)	0.198 (11)	0.003 (7)	0.068 (8)	−0.023 (8)
C39	0.109 (8)	0.066 (6)	0.206 (13)	0.016 (6)	0.058 (9)	0.005 (7)
C43	0.130 (10)	0.128 (10)	0.167 (11)	0.064 (8)	0.021 (9)	−0.019 (9)
C44	0.123 (7)	0.102 (7)	0.174 (8)	0.011 (6)	0.057 (7)	−0.028 (7)
C45	0.125 (8)	0.147 (8)	0.189 (10)	0.011 (8)	0.059 (8)	−0.016 (8)
O8A	0.165 (13)	0.079 (10)	0.129 (12)	0.018 (10)	0.005 (11)	0.007 (10)
C40A	0.111 (10)	0.044 (7)	0.105 (8)	−0.016 (8)	0.030 (8)	0.015 (9)
C41A	0.109 (13)	0.041 (9)	0.100 (11)	−0.003 (9)	0.006 (10)	−0.007 (9)
C42A	0.120 (14)	0.058 (11)	0.115 (13)	0.006 (12)	0.013 (12)	0.003 (12)
O8B	0.180 (13)	0.047 (7)	0.119 (10)	−0.007 (7)	0.022 (9)	−0.023 (6)
C40B	0.112 (9)	0.039 (6)	0.101 (7)	−0.013 (8)	0.027 (8)	0.011 (9)
C41B	0.114 (12)	0.082 (13)	0.109 (11)	−0.007 (12)	0.010 (11)	0.021 (12)
C42B	0.107 (12)	0.066 (11)	0.091 (11)	0.001 (10)	0.011 (10)	0.013 (10)

### [4-(6-bromo-2-methoxyquinolin-3-yl)-3-hydroxy-3-(naphthalen-1-yl)-4-phenylbutyl]dimethylazanium 3-carboxyprop-2-enoate–hexane–acetone (1/0.248/1) (acetone_hexane) Geometric parameters (Å, º)

**Table:**

Br1—C27	1.891 (3)	C21—H21	0.9500
O1—C2	1.422 (3)	C22—H22	0.9500
O1—H1O	0.8400	C23—C24	1.362 (4)
O2—C31	1.344 (3)	C23—C31	1.441 (3)
O2—C32	1.438 (3)	C24—C25	1.424 (3)
O3—C33	1.230 (3)	C24—H24	0.9500
O4—C33	1.271 (3)	C25—C30	1.410 (4)
O4—H5	1.24 (5)	C25—C26	1.414 (4)
O5—C36	1.283 (3)	C26—C27	1.370 (4)
O5—H5	1.18 (5)	C26—H26	0.9500
O6—C36	1.222 (3)	C27—C28	1.407 (5)
N1—C6	1.485 (3)	C28—C29	1.371 (5)
N1—C5	1.495 (3)	C28—H28	0.9500
N1—C4	1.502 (3)	C29—C30	1.415 (3)
N1—H1	1.0000	C29—H29	0.9500
N2—C31	1.307 (3)	C32—H32A	0.9800
N2—C30	1.365 (3)	C32—H32B	0.9800
C1—C23	1.522 (3)	C32—H32C	0.9800
C1—C17	1.526 (3)	C33—C34	1.493 (4)
C1—C2	1.580 (3)	C34—C35	1.332 (4)
C1—H1A	1.0000	C34—H34	0.9500
C2—C7	1.541 (3)	C35—C36	1.489 (4)
C2—C3	1.542 (3)	C35—H35	0.9500
C3—C4	1.519 (3)	O7—C37	1.19 (2)
C3—H3A	0.9900	C37—C38	1.443 (19)
C3—H3B	0.9900	C37—C39	1.463 (19)
C4—H4A	0.9900	C38—H38A	0.9800
C4—H4B	0.9900	C38—H38B	0.9800
C5—H5A	0.9800	C38—H38C	0.9800
C5—H5B	0.9800	C39—H39A	0.9800
C5—H5C	0.9800	C39—H39B	0.9800
C6—H6A	0.9800	C39—H39C	0.9800
C6—H6B	0.9800	C43—C44	1.544 (13)
C6—H6C	0.9800	C43—H43A	0.9800
C7—C8	1.377 (4)	C43—H43B	0.9800
C7—C16	1.436 (4)	C43—H43C	0.9800
C8—C9	1.429 (4)	C44—C45	1.501 (13)
C8—H8	0.9500	C44—H44A	0.9900
C9—C10	1.343 (6)	C44—H44B	0.9900
C9—H9	0.9500	C45—C45^i^	1.473 (13)
C10—C11	1.402 (5)	C45—H45A	0.9900
C10—H10	0.9500	C45—H45B	0.9900
C11—C12	1.426 (5)	O8A—C40A	1.17 (2)
C11—C16	1.440 (3)	C40A—C42A	1.47 (2)
C12—C13	1.344 (7)	C40A—C41A	1.486 (19)
C12—H12	0.9500	C41A—H41A	0.9800
C13—C14	1.398 (5)	C41A—H41B	0.9800
C13—H13	0.9500	C41A—H41C	0.9800
C14—C15	1.375 (4)	C42A—H42A	0.9800
C14—H14	0.9500	C42A—H42B	0.9800
C15—C16	1.425 (4)	C42A—H42C	0.9800
C15—H15	0.9500	O8B—C40B	1.17 (2)
C17—C22	1.381 (4)	C40B—C42B	1.47 (2)
C17—C18	1.382 (5)	C40B—C41B	1.479 (19)
C18—C19	1.396 (4)	C41B—H41D	0.9800
C18—H18	0.9500	C41B—H41E	0.9800
C19—C20	1.369 (6)	C41B—H41F	0.9800
C19—H19	0.9500	C42B—H42D	0.9800
C20—C21	1.368 (7)	C42B—H42E	0.9800
C20—H20	0.9500	C42B—H42F	0.9800
C21—C22	1.392 (5)		
			
C2—O1—H1O	109.5	C30—C25—C24	117.8 (2)
C31—O2—C32	116.7 (2)	C26—C25—C24	122.1 (2)
C33—O4—H5	108 (2)	C27—C26—C25	119.3 (3)
C36—O5—H5	107 (2)	C27—C26—H26	120.3
C6—N1—C5	110.35 (19)	C25—C26—H26	120.3
C6—N1—C4	111.08 (18)	C26—C27—C28	121.4 (3)
C5—N1—C4	112.93 (19)	C26—C27—Br1	119.3 (3)
C6—N1—H1	107.4	C28—C27—Br1	119.3 (2)
C5—N1—H1	107.4	C29—C28—C27	119.7 (3)
C4—N1—H1	107.4	C29—C28—H28	120.2
C31—N2—C30	117.4 (2)	C27—C28—H28	120.2
C23—C1—C17	111.64 (19)	C28—C29—C30	120.8 (3)
C23—C1—C2	110.87 (18)	C28—C29—H29	119.6
C17—C1—C2	114.25 (19)	C30—C29—H29	119.6
C23—C1—H1A	106.5	N2—C30—C25	122.2 (2)
C17—C1—H1A	106.5	N2—C30—C29	119.0 (2)
C2—C1—H1A	106.5	C25—C30—C29	118.8 (3)
O1—C2—C7	107.30 (18)	N2—C31—O2	119.4 (2)
O1—C2—C3	107.82 (18)	N2—C31—C23	125.6 (2)
C7—C2—C3	112.52 (19)	O2—C31—C23	115.0 (2)
O1—C2—C1	108.99 (19)	O2—C32—H32A	109.5
C7—C2—C1	110.39 (18)	O2—C32—H32B	109.5
C3—C2—C1	109.70 (17)	H32A—C32—H32B	109.5
C4—C3—C2	112.94 (18)	O2—C32—H32C	109.5
C4—C3—H3A	109.0	H32A—C32—H32C	109.5
C2—C3—H3A	109.0	H32B—C32—H32C	109.5
C4—C3—H3B	109.0	O3—C33—O4	122.9 (3)
C2—C3—H3B	109.0	O3—C33—C34	117.5 (2)
H3A—C3—H3B	107.8	O4—C33—C34	119.6 (2)
N1—C4—C3	110.99 (18)	C35—C34—C33	130.5 (2)
N1—C4—H4A	109.4	C35—C34—H34	114.8
C3—C4—H4A	109.4	C33—C34—H34	114.8
N1—C4—H4B	109.4	C34—C35—C36	130.9 (2)
C3—C4—H4B	109.4	C34—C35—H35	114.5
H4A—C4—H4B	108.0	C36—C35—H35	114.5
N1—C5—H5A	109.5	O6—C36—O5	122.4 (3)
N1—C5—H5B	109.5	O6—C36—C35	117.9 (3)
H5A—C5—H5B	109.5	O5—C36—C35	119.8 (2)
N1—C5—H5C	109.5	O7—C37—C38	130.1 (18)
H5A—C5—H5C	109.5	O7—C37—C39	116.6 (15)
H5B—C5—H5C	109.5	C38—C37—C39	112.9 (17)
N1—C6—H6A	109.5	C37—C38—H38A	109.5
N1—C6—H6B	109.5	C37—C38—H38B	109.5
H6A—C6—H6B	109.5	H38A—C38—H38B	109.5
N1—C6—H6C	109.5	C37—C38—H38C	109.5
H6A—C6—H6C	109.5	H38A—C38—H38C	109.5
H6B—C6—H6C	109.5	H38B—C38—H38C	109.5
C8—C7—C16	118.5 (2)	C37—C39—H39A	109.5
C8—C7—C2	117.8 (2)	C37—C39—H39B	109.5
C16—C7—C2	123.8 (2)	H39A—C39—H39B	109.5
C7—C8—C9	121.5 (3)	C37—C39—H39C	109.5
C7—C8—H8	119.3	H39A—C39—H39C	109.5
C9—C8—H8	119.3	H39B—C39—H39C	109.5
C10—C9—C8	120.7 (3)	C44—C43—H43A	109.5
C10—C9—H9	119.6	C44—C43—H43B	109.5
C8—C9—H9	119.6	H43A—C43—H43B	109.5
C9—C10—C11	120.5 (3)	C44—C43—H43C	109.5
C9—C10—H10	119.8	H43A—C43—H43C	109.5
C11—C10—H10	119.8	H43B—C43—H43C	109.5
C10—C11—C12	120.5 (3)	C45—C44—C43	114.0 (15)
C10—C11—C16	120.2 (3)	C45—C44—H44A	108.8
C12—C11—C16	119.2 (3)	C43—C44—H44A	108.8
C13—C12—C11	122.7 (3)	C45—C44—H44B	108.8
C13—C12—H12	118.6	C43—C44—H44B	108.8
C11—C12—H12	118.6	H44A—C44—H44B	107.6
C12—C13—C14	118.7 (4)	C45^i^—C45—C44	127.1 (17)
C12—C13—H13	120.6	C45^i^—C45—H45A	105.5
C14—C13—H13	120.6	C44—C45—H45A	105.5
C15—C14—C13	121.4 (4)	C45^i^—C45—H45B	105.5
C15—C14—H14	119.3	C44—C45—H45B	105.5
C13—C14—H14	119.3	H45A—C45—H45B	106.1
C14—C15—C16	122.0 (3)	O8A—C40A—C42A	122 (3)
C14—C15—H15	119.0	O8A—C40A—C41A	126 (3)
C16—C15—H15	119.0	C42A—C40A—C41A	112 (2)
C15—C16—C7	125.4 (2)	C40A—C41A—H41A	109.5
C15—C16—C11	115.9 (3)	C40A—C41A—H41B	109.5
C7—C16—C11	118.7 (3)	H41A—C41A—H41B	109.5
C22—C17—C18	118.1 (3)	C40A—C41A—H41C	109.5
C22—C17—C1	117.6 (3)	H41A—C41A—H41C	109.5
C18—C17—C1	124.3 (2)	H41B—C41A—H41C	109.5
C17—C18—C19	120.9 (3)	C40A—C42A—H42A	109.5
C17—C18—H18	119.6	C40A—C42A—H42B	109.5
C19—C18—H18	119.6	H42A—C42A—H42B	109.5
C20—C19—C18	120.5 (4)	C40A—C42A—H42C	109.5
C20—C19—H19	119.7	H42A—C42A—H42C	109.5
C18—C19—H19	119.7	H42B—C42A—H42C	109.5
C21—C20—C19	118.8 (3)	O8B—C40B—C42B	129 (2)
C21—C20—H20	120.6	O8B—C40B—C41B	116 (3)
C19—C20—H20	120.6	C42B—C40B—C41B	115 (2)
C20—C21—C22	121.2 (4)	C40B—C41B—H41D	109.5
C20—C21—H21	119.4	C40B—C41B—H41E	109.5
C22—C21—H21	119.4	H41D—C41B—H41E	109.5
C17—C22—C21	120.4 (4)	C40B—C41B—H41F	109.5
C17—C22—H22	119.8	H41D—C41B—H41F	109.5
C21—C22—H22	119.8	H41E—C41B—H41F	109.5
C24—C23—C31	115.9 (2)	C40B—C42B—H42D	109.5
C24—C23—C1	123.5 (2)	C40B—C42B—H42E	109.5
C31—C23—C1	120.6 (2)	H42D—C42B—H42E	109.5
C23—C24—C25	120.7 (2)	C40B—C42B—H42F	109.5
C23—C24—H24	119.7	H42D—C42B—H42F	109.5
C25—C24—H24	119.7	H42E—C42B—H42F	109.5
C30—C25—C26	120.1 (2)		
			
C23—C1—C2—O1	−57.4 (2)	C1—C17—C18—C19	−178.5 (3)
C17—C1—C2—O1	69.8 (3)	C17—C18—C19—C20	1.7 (5)
C23—C1—C2—C7	−175.0 (2)	C18—C19—C20—C21	−1.7 (6)
C17—C1—C2—C7	−47.8 (3)	C19—C20—C21—C22	0.4 (7)
C23—C1—C2—C3	60.5 (3)	C18—C17—C22—C21	−1.1 (5)
C17—C1—C2—C3	−172.3 (2)	C1—C17—C22—C21	177.3 (3)
O1—C2—C3—C4	−65.2 (2)	C20—C21—C22—C17	1.1 (6)
C7—C2—C3—C4	52.9 (3)	C17—C1—C23—C24	−38.3 (3)
C1—C2—C3—C4	176.2 (2)	C2—C1—C23—C24	90.3 (3)
C6—N1—C4—C3	163.3 (2)	C17—C1—C23—C31	142.8 (2)
C5—N1—C4—C3	−72.1 (3)	C2—C1—C23—C31	−88.6 (2)
C2—C3—C4—N1	172.86 (19)	C31—C23—C24—C25	2.7 (3)
O1—C2—C7—C8	−0.4 (3)	C1—C23—C24—C25	−176.2 (2)
C3—C2—C7—C8	−118.9 (2)	C23—C24—C25—C30	2.2 (3)
C1—C2—C7—C8	118.2 (2)	C23—C24—C25—C26	−179.7 (2)
O1—C2—C7—C16	179.7 (2)	C30—C25—C26—C27	1.5 (4)
C3—C2—C7—C16	61.3 (3)	C24—C25—C26—C27	−176.5 (3)
C1—C2—C7—C16	−61.6 (3)	C25—C26—C27—C28	−0.3 (5)
C16—C7—C8—C9	0.2 (4)	C25—C26—C27—Br1	178.4 (2)
C2—C7—C8—C9	−179.6 (2)	C26—C27—C28—C29	−0.9 (5)
C7—C8—C9—C10	−0.1 (4)	Br1—C27—C28—C29	−179.6 (2)
C8—C9—C10—C11	0.0 (4)	C27—C28—C29—C30	0.8 (4)
C9—C10—C11—C12	−178.4 (3)	C31—N2—C30—C25	0.7 (3)
C9—C10—C11—C16	−0.1 (4)	C31—N2—C30—C29	−180.0 (2)
C10—C11—C12—C13	178.1 (4)	C26—C25—C30—N2	177.7 (2)
C16—C11—C12—C13	−0.2 (5)	C24—C25—C30—N2	−4.1 (3)
C11—C12—C13—C14	1.0 (6)	C26—C25—C30—C29	−1.6 (3)
C12—C13—C14—C15	−1.0 (6)	C24—C25—C30—C29	176.5 (2)
C13—C14—C15—C16	0.2 (5)	C28—C29—C30—N2	−178.9 (2)
C14—C15—C16—C7	−178.5 (3)	C28—C29—C30—C25	0.4 (3)
C14—C15—C16—C11	0.5 (4)	C30—N2—C31—O2	−174.54 (19)
C8—C7—C16—C15	178.7 (2)	C30—N2—C31—C23	4.9 (3)
C2—C7—C16—C15	−1.5 (4)	C32—O2—C31—N2	−1.1 (3)
C8—C7—C16—C11	−0.3 (3)	C32—O2—C31—C23	179.4 (2)
C2—C7—C16—C11	179.5 (2)	C24—C23—C31—N2	−6.6 (3)
C10—C11—C16—C15	−178.8 (3)	C1—C23—C31—N2	172.3 (2)
C12—C11—C16—C15	−0.6 (4)	C24—C23—C31—O2	172.82 (19)
C10—C11—C16—C7	0.3 (4)	C1—C23—C31—O2	−8.2 (3)
C12—C11—C16—C7	178.6 (3)	O3—C33—C34—C35	−179.5 (3)
C23—C1—C17—C22	−94.4 (3)	O4—C33—C34—C35	1.9 (5)
C2—C1—C17—C22	138.8 (3)	C33—C34—C35—C36	−0.6 (5)
C23—C1—C17—C18	83.9 (3)	C34—C35—C36—O6	−176.7 (3)
C2—C1—C17—C18	−42.9 (4)	C34—C35—C36—O5	3.6 (5)
C22—C17—C18—C19	−0.2 (5)	C43—C44—C45—C45^i^	−149.8 (15)

Symmetry code: (i) −*x*+1, *y*, −*z*+1.

### [4-(6-bromo-2-methoxyquinolin-3-yl)-3-hydroxy-3-(naphthalen-1-yl)-4-phenylbutyl]dimethylazanium 3-carboxyprop-2-enoate–hexane–acetone (1/0.248/1) (acetone_hexane) Hydrogen-bond geometry (Å, º)

**Table:**

*D*—H···*A*	*D*—H	H···*A*	*D*···*A*	*D*—H···*A*
O1—H1*O*···O6^ii^	0.84	1.98	2.816 (3)	175
O5—H5···O4	1.18 (5)	1.24 (5)	2.422 (3)	175 (5)
N1—H1···O3	1.00	1.71	2.709 (3)	174
N1—H1···O4	1.00	2.54	3.212 (3)	125
C32—H32*B*···O7	0.98	2.60	3.337 (8)	132
C34—H34···O7	0.95	2.66	3.600 (9)	171

Symmetry code: (ii) *x*−1/2, *y*−1/2, *z*.

### [4-(6-bromo-2-methoxyquinolin-3-yl)-3-hydroxy-3-(naphthalen-1-yl)-4-phenylbutyl]dimethylazanium 3-carboxyprop-2-enoate (desolvated) Crystal data

**Table:**

C_32_H_32_BrN_2_O_2_^+^·C_4_H_3_O_4_^−^	*F*(000) = 1392
*M**_r_* = 671.56	*D*_x_ = 1.238 Mg m^−^^3^
Monoclinic, *C*2	Mo *K*α radiation, λ = 0.71073 Å
*a* = 15.7494 (12) Å	Cell parameters from 9787 reflections
*b* = 13.3568 (11) Å	θ = 2.5–31.6°
*c* = 17.8634 (14) Å	µ = 1.19 mm^−^^1^
β = 106.500 (3)°	*T* = 150 K
*V* = 3603.0 (5) Å^3^	Block, white
*Z* = 4	0.48 × 0.35 × 0.21 mm

### [4-(6-bromo-2-methoxyquinolin-3-yl)-3-hydroxy-3-(naphthalen-1-yl)-4-phenylbutyl]dimethylazanium 3-carboxyprop-2-enoate (desolvated) Data collection

**Table:**

Bruker AXS D8 Quest with PhotonII CPAD diffractometer	13641 independent reflections
Radiation source: fine focus sealed tube X-ray source	10348 reflections with *I* > 2σ(*I*)
Triumph curved graphite crystal monochromator	*R*_int_ = 0.049
Detector resolution: 7.4074 pixels mm^-1^	θ_max_ = 33.1°, θ_min_ = 2.5°
ω and phi scans	*h* = −24→24
Absorption correction: multi-scan (SADABS; Krause *et al.*, 2015)	*k* = −20→20
*T*_min_ = 0.678, *T*_max_ = 0.740	*l* = −27→27
78980 measured reflections	

### [4-(6-bromo-2-methoxyquinolin-3-yl)-3-hydroxy-3-(naphthalen-1-yl)-4-phenylbutyl]dimethylazanium 3-carboxyprop-2-enoate (desolvated) Refinement

**Table:**

Refinement on *F*^2^	H atoms treated by a mixture of independent and constrained refinement
Least-squares matrix: full	*w* = 1/[σ^2^(*F*_o_^2^) + (0.0725*P*)^2^ + 0.899*P*] where *P* = (*F*_o_^2^ + 2*F*_c_^2^)/3
*R*[*F*^2^ > 2σ(*F*^2^)] = 0.048	(Δ/σ)_max_ = 0.001
*wR*(*F*^2^) = 0.142	Δρ_max_ = 0.89 e Å^−^^3^
*S* = 1.06	Δρ_min_ = −0.74 e Å^−^^3^
13641 reflections	Extinction correction: SHELXL2018/3 (Sheldrick, 2015b), Fc^*^=kFc[1+0.001xFc^2^λ^3^/sin(2θ)]^-1/4^
418 parameters	Extinction coefficient: 0.0113 (14)
1 restraint	Absolute structure: Flack *x* determined using 4138 quotients [(*I*^+^)-(*I*^-^)]/[(*I*^+^)+(*I*^-^)] (Parsons *et al.*, 2013)
Primary atom site location: isomorphous structure methods	Absolute structure parameter: 0.044 (3)
Hydrogen site location: mixed	

### [4-(6-bromo-2-methoxyquinolin-3-yl)-3-hydroxy-3-(naphthalen-1-yl)-4-phenylbutyl]dimethylazanium 3-carboxyprop-2-enoate (desolvated) Special details

**Table:**

Geometry. All esds (except the esd in the dihedral angle between two l.s. planes) are estimated using the full covariance matrix. The cell esds are taken into account individually in the estimation of esds in distances, angles and torsion angles; correlations between esds in cell parameters are only used when they are defined by crystal symmetry. An approximate (isotropic) treatment of cell esds is used for estimating esds involving l.s. planes.
Refinement. Crystals were obtained from the acetone / hexane solvate by drying on a glass slide in air over night. In the solavted structure acetone and hexane molecules are located in infinite channels and slowly vacate the crystal lattice. Crystals become milky overnight when taken out of mother liquor solution and left to dry in air, but retain crystallinity. The structure was solved by isomorphous replacement from the acetone / hexane solvate (structure code 91_2). No substantial electron density was found in the previously solvate occupied channels (largest void peaks are less than 0.5 electrons per cubic Angstrom). Residual electron density was not corrected for. An alternative refinement using the Squeeze algorithm is appended below this cif file.

### [4-(6-bromo-2-methoxyquinolin-3-yl)-3-hydroxy-3-(naphthalen-1-yl)-4-phenylbutyl]dimethylazanium 3-carboxyprop-2-enoate (desolvated) Fractional atomic coordinates and isotropic or equivalent isotropic displacement parameters (Å^2^)

**Table:**

	*x*	*y*	*z*	*U*_iso_*/*U*_eq_	
Br1	0.30125 (4)	0.01859 (5)	0.48058 (3)	0.0879 (2)	
O1	0.48670 (11)	0.26945 (13)	0.16346 (10)	0.0303 (3)	
H1O	0.441024	0.283221	0.176802	0.036*	
O2	0.56663 (13)	0.48363 (14)	0.36126 (10)	0.0353 (4)	
O3	0.48998 (13)	0.69083 (16)	0.21057 (12)	0.0413 (4)	
O4	0.56590 (16)	0.7068 (2)	0.12480 (12)	0.0564 (7)	
O5	0.70940 (16)	0.7676 (2)	0.12134 (12)	0.0530 (6)	
H5	0.648 (3)	0.747 (4)	0.119 (3)	0.064*	
O6	0.83299 (13)	0.81615 (17)	0.20737 (13)	0.0435 (4)	
N1	0.39732 (12)	0.56474 (15)	0.09851 (10)	0.0256 (3)	
H1	0.430746	0.615825	0.136485	0.031*	
N2	0.46078 (14)	0.40519 (19)	0.40536 (11)	0.0344 (4)	
C1	0.60435 (14)	0.32110 (18)	0.27931 (12)	0.0275 (4)	
H1A	0.643297	0.380159	0.299045	0.033*	
C2	0.55201 (14)	0.34533 (16)	0.19164 (12)	0.0248 (4)	
C3	0.50343 (14)	0.44634 (17)	0.18665 (12)	0.0260 (4)	
H3A	0.458870	0.441573	0.215984	0.031*	
H3B	0.546585	0.498846	0.211574	0.031*	
C4	0.45729 (15)	0.47679 (17)	0.10240 (12)	0.0277 (4)	
H4A	0.502527	0.493699	0.075660	0.033*	
H4B	0.422337	0.419506	0.074578	0.033*	
C5	0.31566 (15)	0.5397 (2)	0.12082 (16)	0.0365 (5)	
H5A	0.281758	0.488848	0.085036	0.055*	
H5B	0.279469	0.600029	0.117796	0.055*	
H5C	0.331985	0.513607	0.174296	0.055*	
C6	0.37408 (17)	0.6109 (2)	0.01963 (14)	0.0341 (5)	
H6A	0.351408	0.559273	−0.019970	0.051*	
H6B	0.426940	0.641772	0.010933	0.051*	
H6C	0.328558	0.662211	0.015885	0.051*	
C7	0.61578 (15)	0.34283 (17)	0.14019 (12)	0.0270 (4)	
C8	0.59948 (17)	0.2735 (2)	0.08053 (13)	0.0332 (5)	
H8	0.550112	0.229909	0.072930	0.040*	
C9	0.6545 (2)	0.2657 (2)	0.03022 (14)	0.0405 (6)	
H9	0.640743	0.218480	−0.011245	0.049*	
C10	0.7269 (2)	0.3254 (2)	0.04101 (15)	0.0410 (6)	
H10	0.764008	0.318635	0.007665	0.049*	
C11	0.74727 (17)	0.3971 (2)	0.10125 (15)	0.0372 (5)	
C12	0.8226 (2)	0.4605 (3)	0.1115 (2)	0.0514 (7)	
H12	0.859768	0.451730	0.078446	0.062*	
C13	0.8427 (2)	0.5325 (3)	0.1668 (2)	0.0540 (8)	
H13	0.892496	0.574758	0.171714	0.065*	
C14	0.7884 (2)	0.5441 (2)	0.21744 (19)	0.0449 (6)	
H14	0.802662	0.593800	0.257128	0.054*	
C15	0.71535 (16)	0.4843 (2)	0.20964 (15)	0.0344 (5)	
H15	0.679762	0.494282	0.243939	0.041*	
C16	0.69118 (15)	0.40772 (18)	0.15192 (13)	0.0298 (4)	
C17	0.66522 (16)	0.2308 (2)	0.28868 (13)	0.0323 (5)	
C18	0.6404 (2)	0.1395 (2)	0.25147 (17)	0.0405 (6)	
H18	0.583556	0.132423	0.215075	0.049*	
C19	0.6984 (3)	0.0582 (3)	0.2672 (2)	0.0528 (8)	
H19	0.680733	−0.003545	0.241114	0.063*	
C20	0.7808 (3)	0.0663 (3)	0.3199 (2)	0.0640 (11)	
H20	0.819334	0.010108	0.331427	0.077*	
C21	0.8068 (2)	0.1574 (4)	0.3561 (2)	0.0649 (11)	
H21	0.864178	0.163800	0.391693	0.078*	
C22	0.74995 (18)	0.2406 (3)	0.34113 (16)	0.0457 (7)	
H22	0.768692	0.302737	0.366253	0.055*	
C23	0.54190 (14)	0.31290 (19)	0.33007 (12)	0.0283 (4)	
C24	0.50509 (16)	0.2257 (2)	0.34414 (13)	0.0318 (4)	
H24	0.519769	0.164956	0.323111	0.038*	
C25	0.44446 (15)	0.2244 (2)	0.39028 (12)	0.0347 (5)	
C26	0.40766 (19)	0.1342 (3)	0.40905 (16)	0.0439 (6)	
H26	0.423811	0.071423	0.392136	0.053*	
C27	0.3479 (2)	0.1395 (3)	0.45243 (17)	0.0510 (8)	
C28	0.32176 (19)	0.2313 (3)	0.47678 (15)	0.0518 (8)	
H28	0.279070	0.233132	0.505128	0.062*	
C29	0.35797 (17)	0.3182 (3)	0.45956 (14)	0.0437 (7)	
H29	0.340071	0.380224	0.476292	0.052*	
C30	0.42187 (15)	0.3178 (2)	0.41712 (12)	0.0352 (5)	
C31	0.51945 (15)	0.40092 (19)	0.36697 (12)	0.0299 (4)	
C32	0.5516 (3)	0.5718 (3)	0.4008 (2)	0.0502 (7)	
H32A	0.491738	0.596937	0.376121	0.075*	
H32B	0.595047	0.623067	0.397856	0.075*	
H32C	0.557740	0.555914	0.455671	0.075*	
C33	0.55716 (17)	0.71776 (19)	0.19320 (14)	0.0340 (5)	
C34	0.63126 (18)	0.7609 (2)	0.25666 (14)	0.0361 (5)	
H34	0.618957	0.769093	0.305373	0.043*	
C35	0.71202 (17)	0.7897 (2)	0.25575 (14)	0.0351 (5)	
H35	0.748533	0.813471	0.304385	0.042*	
C36	0.75461 (17)	0.79078 (19)	0.19096 (15)	0.0343 (5)	

### [4-(6-bromo-2-methoxyquinolin-3-yl)-3-hydroxy-3-(naphthalen-1-yl)-4-phenylbutyl]dimethylazanium 3-carboxyprop-2-enoate (desolvated) Atomic displacement parameters (Å^2^)

**Table:**

	*U*^11^	*U*^22^	*U*^33^	*U*^12^	*U*^13^	*U*^23^
Br1	0.0857 (3)	0.1020 (4)	0.0935 (4)	−0.0386 (3)	0.0537 (3)	0.0023 (3)
O1	0.0275 (7)	0.0320 (8)	0.0306 (7)	−0.0008 (6)	0.0072 (6)	−0.0025 (6)
O2	0.0405 (9)	0.0364 (8)	0.0298 (8)	0.0055 (7)	0.0111 (7)	−0.0013 (7)
O3	0.0372 (9)	0.0457 (10)	0.0450 (10)	−0.0101 (8)	0.0182 (8)	−0.0100 (8)
O4	0.0453 (11)	0.0926 (19)	0.0346 (9)	−0.0313 (12)	0.0168 (8)	−0.0209 (11)
O5	0.0469 (11)	0.0836 (17)	0.0327 (9)	−0.0259 (12)	0.0184 (8)	−0.0088 (10)
O6	0.0305 (9)	0.0483 (11)	0.0535 (11)	−0.0052 (8)	0.0146 (8)	0.0024 (9)
N1	0.0236 (7)	0.0280 (8)	0.0255 (7)	0.0031 (6)	0.0073 (6)	0.0021 (6)
N2	0.0287 (9)	0.0520 (12)	0.0221 (8)	0.0112 (8)	0.0064 (7)	−0.0002 (8)
C1	0.0254 (9)	0.0347 (10)	0.0226 (8)	0.0063 (8)	0.0071 (7)	0.0028 (7)
C2	0.0244 (8)	0.0284 (9)	0.0214 (8)	0.0036 (7)	0.0064 (7)	0.0003 (7)
C3	0.0253 (9)	0.0304 (9)	0.0219 (8)	0.0067 (7)	0.0061 (7)	0.0012 (7)
C4	0.0280 (9)	0.0312 (9)	0.0232 (8)	0.0087 (8)	0.0064 (7)	0.0013 (7)
C5	0.0242 (9)	0.0441 (13)	0.0437 (12)	0.0039 (9)	0.0139 (9)	0.0103 (10)
C6	0.0333 (11)	0.0378 (12)	0.0302 (10)	0.0073 (9)	0.0074 (8)	0.0085 (9)
C7	0.0290 (9)	0.0315 (10)	0.0214 (8)	0.0086 (8)	0.0087 (7)	0.0027 (7)
C8	0.0369 (11)	0.0386 (12)	0.0238 (9)	0.0105 (9)	0.0080 (8)	−0.0007 (8)
C9	0.0529 (15)	0.0447 (13)	0.0256 (10)	0.0206 (12)	0.0141 (10)	0.0022 (9)
C10	0.0478 (14)	0.0484 (14)	0.0343 (11)	0.0228 (12)	0.0234 (10)	0.0121 (10)
C11	0.0342 (11)	0.0453 (13)	0.0370 (11)	0.0131 (10)	0.0181 (9)	0.0110 (10)
C12	0.0421 (14)	0.0610 (19)	0.0604 (18)	0.0074 (13)	0.0295 (13)	0.0152 (15)
C13	0.0372 (13)	0.0572 (18)	0.072 (2)	−0.0033 (13)	0.0230 (14)	0.0087 (16)
C14	0.0361 (12)	0.0448 (15)	0.0529 (15)	−0.0046 (10)	0.0112 (11)	0.0022 (12)
C15	0.0313 (10)	0.0385 (11)	0.0341 (11)	0.0017 (9)	0.0103 (8)	0.0019 (9)
C16	0.0273 (9)	0.0361 (11)	0.0276 (9)	0.0074 (8)	0.0103 (7)	0.0053 (8)
C17	0.0295 (10)	0.0427 (12)	0.0273 (9)	0.0131 (9)	0.0121 (8)	0.0099 (9)
C18	0.0428 (13)	0.0393 (12)	0.0435 (13)	0.0148 (11)	0.0191 (11)	0.0105 (11)
C19	0.0625 (19)	0.0432 (15)	0.0626 (18)	0.0229 (14)	0.0338 (16)	0.0147 (13)
C20	0.064 (2)	0.073 (2)	0.0621 (19)	0.0466 (19)	0.0299 (16)	0.0284 (18)
C21	0.0435 (16)	0.098 (3)	0.0519 (18)	0.0396 (19)	0.0105 (13)	0.0207 (19)
C22	0.0313 (11)	0.0681 (18)	0.0374 (12)	0.0196 (12)	0.0092 (10)	0.0089 (12)
C23	0.0244 (9)	0.0389 (11)	0.0211 (8)	0.0065 (8)	0.0057 (7)	0.0018 (8)
C24	0.0293 (10)	0.0424 (12)	0.0239 (8)	0.0034 (9)	0.0079 (7)	0.0023 (9)
C25	0.0253 (9)	0.0568 (15)	0.0215 (8)	0.0000 (10)	0.0057 (7)	0.0017 (9)
C26	0.0369 (12)	0.0637 (18)	0.0328 (11)	−0.0080 (12)	0.0126 (10)	0.0021 (12)
C27	0.0396 (14)	0.080 (2)	0.0355 (12)	−0.0165 (15)	0.0143 (11)	0.0000 (14)
C28	0.0312 (11)	0.098 (3)	0.0290 (11)	−0.0093 (14)	0.0125 (9)	−0.0069 (14)
C29	0.0285 (11)	0.080 (2)	0.0238 (10)	0.0015 (12)	0.0084 (8)	−0.0063 (11)
C30	0.0225 (9)	0.0622 (16)	0.0193 (8)	0.0060 (10)	0.0035 (7)	0.0003 (9)
C31	0.0281 (10)	0.0399 (11)	0.0203 (8)	0.0082 (8)	0.0047 (7)	0.0021 (8)
C32	0.0598 (18)	0.0423 (14)	0.0522 (16)	0.0058 (13)	0.0219 (14)	−0.0097 (13)
C33	0.0350 (11)	0.0358 (11)	0.0327 (10)	−0.0072 (9)	0.0120 (9)	−0.0062 (9)
C34	0.0392 (12)	0.0422 (12)	0.0290 (10)	−0.0094 (10)	0.0132 (9)	−0.0066 (9)
C35	0.0336 (11)	0.0424 (12)	0.0288 (10)	−0.0060 (9)	0.0079 (8)	−0.0024 (9)
C36	0.0341 (11)	0.0344 (11)	0.0365 (11)	−0.0045 (9)	0.0138 (9)	0.0014 (9)

### [4-(6-bromo-2-methoxyquinolin-3-yl)-3-hydroxy-3-(naphthalen-1-yl)-4-phenylbutyl]dimethylazanium 3-carboxyprop-2-enoate (desolvated) Geometric parameters (Å, º)

**Table:**

Br1—C27	1.900 (4)	C11—C12	1.426 (5)
O1—C2	1.430 (3)	C11—C16	1.440 (3)
O1—H1O	0.8400	C12—C13	1.350 (6)
O2—C31	1.351 (3)	C12—H12	0.9500
O2—C32	1.427 (3)	C13—C14	1.419 (5)
O3—C33	1.238 (3)	C13—H13	0.9500
O4—C33	1.276 (3)	C14—C15	1.376 (4)
O4—H5	1.43 (5)	C14—H14	0.9500
O5—C36	1.283 (3)	C15—C16	1.425 (4)
O5—H5	0.99 (5)	C15—H15	0.9500
O6—C36	1.233 (3)	C17—C18	1.391 (4)
N1—C6	1.486 (3)	C17—C22	1.402 (4)
N1—C5	1.489 (3)	C18—C19	1.395 (4)
N1—C4	1.497 (3)	C18—H18	0.9500
N1—H1	1.0000	C19—C20	1.374 (6)
N2—C31	1.299 (3)	C19—H19	0.9500
N2—C30	1.363 (4)	C20—C21	1.384 (7)
C1—C23	1.519 (3)	C20—H20	0.9500
C1—C17	1.520 (3)	C21—C22	1.404 (5)
C1—C2	1.582 (3)	C21—H21	0.9500
C1—H1A	1.0000	C22—H22	0.9500
C2—C3	1.541 (3)	C23—C24	1.356 (4)
C2—C7	1.542 (3)	C23—C31	1.440 (3)
C3—C4	1.529 (3)	C24—C25	1.428 (3)
C3—H3A	0.9900	C24—H24	0.9500
C3—H3B	0.9900	C25—C30	1.417 (4)
C4—H4A	0.9900	C25—C26	1.418 (4)
C4—H4B	0.9900	C26—C27	1.381 (4)
C5—H5A	0.9800	C26—H26	0.9500
C5—H5B	0.9800	C27—C28	1.402 (6)
C5—H5C	0.9800	C28—C29	1.366 (5)
C6—H6A	0.9800	C28—H28	0.9500
C6—H6B	0.9800	C29—C30	1.422 (3)
C6—H6C	0.9800	C29—H29	0.9500
C7—C8	1.380 (3)	C32—H32A	0.9800
C7—C16	1.437 (3)	C32—H32B	0.9800
C8—C9	1.419 (3)	C32—H32C	0.9800
C8—H8	0.9500	C33—C34	1.493 (3)
C9—C10	1.358 (5)	C34—C35	1.333 (4)
C9—H9	0.9500	C34—H34	0.9500
C10—C11	1.408 (4)	C35—C36	1.493 (4)
C10—H10	0.9500	C35—H35	0.9500
			
C2—O1—H1O	109.5	C13—C14—H14	119.7
C31—O2—C32	117.7 (2)	C14—C15—C16	122.3 (2)
C33—O4—H5	111.7 (19)	C14—C15—H15	118.8
C36—O5—H5	112 (3)	C16—C15—H15	118.8
C6—N1—C5	110.39 (18)	C15—C16—C7	125.3 (2)
C6—N1—C4	110.62 (17)	C15—C16—C11	116.1 (2)
C5—N1—C4	113.13 (18)	C7—C16—C11	118.6 (2)
C6—N1—H1	107.5	C18—C17—C22	119.1 (2)
C5—N1—H1	107.5	C18—C17—C1	124.1 (2)
C4—N1—H1	107.5	C22—C17—C1	116.8 (3)
C31—N2—C30	117.5 (2)	C17—C18—C19	120.4 (3)
C23—C1—C17	111.85 (18)	C17—C18—H18	119.8
C23—C1—C2	111.21 (17)	C19—C18—H18	119.8
C17—C1—C2	113.98 (17)	C20—C19—C18	121.0 (4)
C23—C1—H1A	106.4	C20—C19—H19	119.5
C17—C1—H1A	106.4	C18—C19—H19	119.5
C2—C1—H1A	106.4	C19—C20—C21	119.0 (3)
O1—C2—C3	107.73 (17)	C19—C20—H20	120.5
O1—C2—C7	107.45 (17)	C21—C20—H20	120.5
C3—C2—C7	112.40 (17)	C20—C21—C22	121.3 (3)
O1—C2—C1	108.90 (17)	C20—C21—H21	119.4
C3—C2—C1	110.30 (16)	C22—C21—H21	119.4
C7—C2—C1	109.94 (16)	C17—C22—C21	119.2 (4)
C4—C3—C2	112.29 (17)	C17—C22—H22	120.4
C4—C3—H3A	109.1	C21—C22—H22	120.4
C2—C3—H3A	109.1	C24—C23—C31	116.3 (2)
C4—C3—H3B	109.1	C24—C23—C1	123.8 (2)
C2—C3—H3B	109.1	C31—C23—C1	119.9 (2)
H3A—C3—H3B	107.9	C23—C24—C25	120.6 (2)
N1—C4—C3	111.83 (17)	C23—C24—H24	119.7
N1—C4—H4A	109.3	C25—C24—H24	119.7
C3—C4—H4A	109.3	C30—C25—C26	120.6 (2)
N1—C4—H4B	109.3	C30—C25—C24	117.2 (2)
C3—C4—H4B	109.3	C26—C25—C24	122.2 (3)
H4A—C4—H4B	107.9	C27—C26—C25	118.6 (3)
N1—C5—H5A	109.5	C27—C26—H26	120.7
N1—C5—H5B	109.5	C25—C26—H26	120.7
H5A—C5—H5B	109.5	C26—C27—C28	121.7 (3)
N1—C5—H5C	109.5	C26—C27—Br1	118.8 (3)
H5A—C5—H5C	109.5	C28—C27—Br1	119.5 (2)
H5B—C5—H5C	109.5	C29—C28—C27	119.7 (3)
N1—C6—H6A	109.5	C29—C28—H28	120.1
N1—C6—H6B	109.5	C27—C28—H28	120.1
H6A—C6—H6B	109.5	C28—C29—C30	121.4 (3)
N1—C6—H6C	109.5	C28—C29—H29	119.3
H6A—C6—H6C	109.5	C30—C29—H29	119.3
H6B—C6—H6C	109.5	N2—C30—C25	122.3 (2)
C8—C7—C16	118.8 (2)	N2—C30—C29	119.7 (3)
C8—C7—C2	117.7 (2)	C25—C30—C29	117.9 (3)
C16—C7—C2	123.44 (19)	N2—C31—O2	119.5 (2)
C7—C8—C9	121.7 (3)	N2—C31—C23	125.5 (2)
C7—C8—H8	119.1	O2—C31—C23	114.9 (2)
C9—C8—H8	119.1	O2—C32—H32A	109.5
C10—C9—C8	120.4 (2)	O2—C32—H32B	109.5
C10—C9—H9	119.8	H32A—C32—H32B	109.5
C8—C9—H9	119.8	O2—C32—H32C	109.5
C9—C10—C11	120.5 (2)	H32A—C32—H32C	109.5
C9—C10—H10	119.7	H32B—C32—H32C	109.5
C11—C10—H10	119.7	O3—C33—O4	122.6 (2)
C10—C11—C12	120.2 (3)	O3—C33—C34	117.5 (2)
C10—C11—C16	120.0 (3)	O4—C33—C34	119.9 (2)
C12—C11—C16	119.8 (3)	C35—C34—C33	130.2 (2)
C13—C12—C11	122.1 (3)	C35—C34—H34	114.9
C13—C12—H12	119.0	C33—C34—H34	114.9
C11—C12—H12	119.0	C34—C35—C36	130.7 (2)
C12—C13—C14	119.1 (3)	C34—C35—H35	114.6
C12—C13—H13	120.5	C36—C35—H35	114.6
C14—C13—H13	120.5	O6—C36—O5	122.6 (2)
C15—C14—C13	120.7 (3)	O6—C36—C35	117.3 (2)
C15—C14—H14	119.7	O5—C36—C35	120.1 (2)
			
C23—C1—C2—O1	−56.9 (2)	C1—C17—C18—C19	−175.7 (2)
C17—C1—C2—O1	70.6 (2)	C17—C18—C19—C20	0.4 (5)
C23—C1—C2—C3	61.1 (2)	C18—C19—C20—C21	−1.7 (5)
C17—C1—C2—C3	−171.37 (19)	C19—C20—C21—C22	1.4 (6)
C23—C1—C2—C7	−174.42 (19)	C18—C17—C22—C21	−1.4 (4)
C17—C1—C2—C7	−46.9 (3)	C1—C17—C22—C21	175.7 (3)
O1—C2—C3—C4	−65.4 (2)	C20—C21—C22—C17	0.1 (5)
C7—C2—C3—C4	52.8 (2)	C17—C1—C23—C24	−36.6 (3)
C1—C2—C3—C4	175.85 (18)	C2—C1—C23—C24	92.1 (2)
C6—N1—C4—C3	163.58 (19)	C17—C1—C23—C31	142.8 (2)
C5—N1—C4—C3	−72.0 (2)	C2—C1—C23—C31	−88.5 (2)
C2—C3—C4—N1	169.78 (18)	C31—C23—C24—C25	2.8 (3)
O1—C2—C7—C8	0.3 (3)	C1—C23—C24—C25	−177.8 (2)
C3—C2—C7—C8	−118.1 (2)	C23—C24—C25—C30	2.8 (3)
C1—C2—C7—C8	118.7 (2)	C23—C24—C25—C26	−177.2 (2)
O1—C2—C7—C16	−178.93 (19)	C30—C25—C26—C27	1.5 (4)
C3—C2—C7—C16	62.7 (2)	C24—C25—C26—C27	−178.5 (2)
C1—C2—C7—C16	−60.5 (3)	C25—C26—C27—C28	1.2 (4)
C16—C7—C8—C9	−0.8 (3)	C25—C26—C27—Br1	−178.0 (2)
C2—C7—C8—C9	−180.0 (2)	C26—C27—C28—C29	−2.0 (4)
C7—C8—C9—C10	1.7 (4)	Br1—C27—C28—C29	177.2 (2)
C8—C9—C10—C11	−1.3 (4)	C27—C28—C29—C30	0.1 (4)
C9—C10—C11—C12	−179.1 (3)	C31—N2—C30—C25	1.1 (3)
C9—C10—C11—C16	−0.1 (4)	C31—N2—C30—C29	179.3 (2)
C10—C11—C12—C13	177.7 (3)	C26—C25—C30—N2	174.9 (2)
C16—C11—C12—C13	−1.4 (5)	C24—C25—C30—N2	−5.1 (3)
C11—C12—C13—C14	1.4 (5)	C26—C25—C30—C29	−3.3 (3)
C12—C13—C14—C15	−1.1 (5)	C24—C25—C30—C29	176.7 (2)
C13—C14—C15—C16	0.8 (4)	C28—C29—C30—N2	−175.8 (2)
C14—C15—C16—C7	−179.7 (2)	C28—C29—C30—C25	2.5 (3)
C14—C15—C16—C11	−0.7 (4)	C30—N2—C31—O2	−172.82 (19)
C8—C7—C16—C15	178.4 (2)	C30—N2—C31—C23	5.4 (3)
C2—C7—C16—C15	−2.4 (3)	C32—O2—C31—N2	2.3 (3)
C8—C7—C16—C11	−0.6 (3)	C32—O2—C31—C23	−176.1 (2)
C2—C7—C16—C11	178.6 (2)	C24—C23—C31—N2	−7.4 (3)
C10—C11—C16—C15	−178.1 (2)	C1—C23—C31—N2	173.1 (2)
C12—C11—C16—C15	0.9 (3)	C24—C23—C31—O2	170.88 (19)
C10—C11—C16—C7	1.0 (3)	C1—C23—C31—O2	−8.6 (3)
C12—C11—C16—C7	−180.0 (2)	O3—C33—C34—C35	175.3 (3)
C23—C1—C17—C18	80.4 (3)	O4—C33—C34—C35	−2.3 (5)
C2—C1—C17—C18	−46.8 (3)	C33—C34—C35—C36	1.6 (5)
C23—C1—C17—C22	−96.6 (3)	C34—C35—C36—O6	−175.9 (3)
C2—C1—C17—C22	136.2 (2)	C34—C35—C36—O5	5.4 (5)
C22—C17—C18—C19	1.2 (4)		

### [4-(6-bromo-2-methoxyquinolin-3-yl)-3-hydroxy-3-(naphthalen-1-yl)-4-phenylbutyl]dimethylazanium 3-carboxyprop-2-enoate (desolvated) Hydrogen-bond geometry (Å, º)

**Table:**

*D*—H···*A*	*D*—H	H···*A*	*D*···*A*	*D*—H···*A*
O1—H1*O*···O6^i^	0.84	1.98	2.818 (3)	180
O5—H5···O4	0.99 (5)	1.43 (5)	2.419 (3)	172 (5)
N1—H1···O3	1.00	1.71	2.704 (3)	172
N1—H1···O4	1.00	2.51	3.188 (3)	125

Symmetry code: (i) *x*−1/2, *y*−1/2, *z*.

### [4-(6-bromo-2-methoxyquinolin-3-yl)-3-hydroxy-3-(naphthalen-1-yl)-4-phenylbutyl]dimethylazanium 3-carboxyprop-2-enoate (desolvated_sq) Crystal data

**Table:**

C_32_H_32_BrN_2_O_2_^+^·C_4_H_3_O_4_^−^	*F*(000) = 1392
*M**_r_* = 671.56	*D*_x_ = 1.238 Mg m^−^^3^
Monoclinic, *C*2	Mo *K*α radiation, λ = 0.71073 Å
*a* = 15.7494 (12) Å	Cell parameters from 9787 reflections
*b* = 13.3568 (11) Å	θ = 2.5–31.6°
*c* = 17.8634 (14) Å	µ = 1.19 mm^−^^1^
β = 106.500 (3)°	*T* = 150 K
*V* = 3603.0 (5) Å^3^	Block, white
*Z* = 4	0.48 × 0.35 × 0.21 mm

### [4-(6-bromo-2-methoxyquinolin-3-yl)-3-hydroxy-3-(naphthalen-1-yl)-4-phenylbutyl]dimethylazanium 3-carboxyprop-2-enoate (desolvated_sq) Data collection

**Table:**

Bruker AXS D8 Quest with PhotonII CPAD diffractometer	13639 independent reflections
Radiation source: fine focus sealed tube X-ray source	10346 reflections with *I* > 2σ(*I*)
Triumph curved graphite crystal monochromator	*R*_int_ = 0.049
Detector resolution: 7.4074 pixels mm^-1^	θ_max_ = 33.1°, θ_min_ = 2.5°
ω and phi scans	*h* = −24→24
Absorption correction: multi-scan (SADABS; Krause *et al.*, 2015)	*k* = −20→20
*T*_min_ = 0.678, *T*_max_ = 0.740	*l* = −27→27
78970 measured reflections	

### [4-(6-bromo-2-methoxyquinolin-3-yl)-3-hydroxy-3-(naphthalen-1-yl)-4-phenylbutyl]dimethylazanium 3-carboxyprop-2-enoate (desolvated_sq) Refinement

**Table:**

Refinement on *F*^2^	H atoms treated by a mixture of independent and constrained refinement
Least-squares matrix: full	*w* = 1/[σ^2^(*F*_o_^2^) + (0.0554*P*)^2^ + 0.5224*P*] where *P* = (*F*_o_^2^ + 2*F*_c_^2^)/3
*R*[*F*^2^ > 2σ(*F*^2^)] = 0.042	(Δ/σ)_max_ = 0.011
*wR*(*F*^2^) = 0.119	Δρ_max_ = 0.76 e Å^−^^3^
*S* = 1.06	Δρ_min_ = −0.63 e Å^−^^3^
13639 reflections	Extinction correction: SHELXL2018/3 (Sheldrick, 2015b), Fc^*^=kFc[1+0.001xFc^2^λ^3^/sin(2θ)]^-1/4^
418 parameters	Extinction coefficient: 0.0076 (12)
1 restraint	Absolute structure: Flack *x* determined using 4139 quotients [(*I*^+^)-(*I*^-^)]/[(*I*^+^)+(*I*^-^)] (Parsons *et al.*, 2013)
Primary atom site location: isomorphous structure methods	Absolute structure parameter: 0.044 (3)
Hydrogen site location: mixed	

### [4-(6-bromo-2-methoxyquinolin-3-yl)-3-hydroxy-3-(naphthalen-1-yl)-4-phenylbutyl]dimethylazanium 3-carboxyprop-2-enoate (desolvated_sq) Special details

**Table:**

Geometry. All esds (except the esd in the dihedral angle between two l.s. planes) are estimated using the full covariance matrix. The cell esds are taken into account individually in the estimation of esds in distances, angles and torsion angles; correlations between esds in cell parameters are only used when they are defined by crystal symmetry. An approximate (isotropic) treatment of cell esds is used for estimating esds involving l.s. planes.
Refinement. Crystals were obtained from the acetone / hexane solvate by drying on a glass slide in air over night. In the solavted structure acetone and hexane molecules are located in infinite channels and slowly vacate the crystal lattice. Crystals become milky overnight when taken out of mother liquor solution and left to dry in air, but retain crystallinity. The structure was solved by isomorphous replacement from the acetone / hexane solvate (structure code 91_2). The structure contains solvent accessible voids of 607 Ang3 combined. No substantial electron density peaks were found in the solvent accessible voids (less than 0.5 electron per cubic Angstrom) and the residual electron density peaks are not arranged in an interpretable pattern. The structure factors were instead augmented via reverse Fourier transform methods using the SQUEEZE routine (P. van der Sluis & A.L. Spek (1990). Acta Cryst. A46, 194-201) as implemented in the program Platon. The resultant FAB file containing the structure factor contribution from the electron content of the void space was used in together with the original hkl file in the further refinement. (The FAB file with details of the Squeeze results is appended to this cif file). The Squeeze procedure corrected for 66 electrons within the solvent accessible voids, equivalent to 1.14 molecules of acetone per unit cell, or 0.28 acetone per cation / anion pair. Prior to desolvation, 1 molecule of acetone and half a molecule of hexane were determined per cation / anion pair (equivalent to 202 electrons per unit cell).

### [4-(6-bromo-2-methoxyquinolin-3-yl)-3-hydroxy-3-(naphthalen-1-yl)-4-phenylbutyl]dimethylazanium 3-carboxyprop-2-enoate (desolvated_sq) Fractional atomic coordinates and isotropic or equivalent isotropic displacement parameters (Å^2^)

**Table:**

	*x*	*y*	*z*	*U*_iso_*/*U*_eq_	
Br1	0.30118 (3)	0.01862 (4)	0.48056 (3)	0.08775 (18)	
O1	0.48679 (9)	0.26938 (11)	0.16351 (8)	0.0299 (3)	
H1O	0.440910	0.283392	0.176451	0.036*	
O2	0.56679 (11)	0.48352 (12)	0.36136 (9)	0.0353 (3)	
O3	0.49001 (11)	0.69078 (14)	0.21060 (10)	0.0412 (4)	
O4	0.56582 (13)	0.7067 (2)	0.12477 (10)	0.0561 (6)	
O5	0.70934 (14)	0.76777 (19)	0.12131 (10)	0.0527 (5)	
H5	0.647 (3)	0.747 (3)	0.117 (2)	0.063*	
O6	0.83287 (11)	0.81603 (15)	0.20731 (11)	0.0433 (4)	
N1	0.39737 (10)	0.56465 (13)	0.09851 (9)	0.0253 (3)	
H1	0.430837	0.615719	0.136472	0.030*	
N2	0.46071 (12)	0.40514 (16)	0.40537 (10)	0.0341 (4)	
C1	0.60427 (12)	0.32105 (15)	0.27931 (10)	0.0273 (3)	
H1A	0.643206	0.380148	0.298957	0.033*	
C2	0.55192 (12)	0.34523 (14)	0.19163 (10)	0.0246 (3)	
C3	0.50350 (12)	0.44640 (15)	0.18668 (10)	0.0260 (3)	
H3A	0.459011	0.441802	0.216096	0.031*	
H3B	0.546765	0.498851	0.211502	0.031*	
C4	0.45728 (13)	0.47672 (15)	0.10240 (10)	0.0274 (3)	
H4A	0.502469	0.493565	0.075593	0.033*	
H4B	0.422292	0.419415	0.074655	0.033*	
C5	0.31575 (13)	0.53959 (18)	0.12091 (14)	0.0361 (4)	
H5A	0.280149	0.491610	0.083411	0.054*	
H5B	0.281213	0.600655	0.120786	0.054*	
H5C	0.332157	0.510016	0.173268	0.054*	
C6	0.37409 (14)	0.61089 (17)	0.01966 (12)	0.0337 (4)	
H6A	0.350953	0.559350	−0.019928	0.051*	
H6B	0.427036	0.641283	0.010792	0.051*	
H6C	0.328913	0.662530	0.016106	0.051*	
C7	0.61572 (13)	0.34273 (15)	0.14022 (10)	0.0266 (3)	
C8	0.59959 (15)	0.27345 (17)	0.08056 (11)	0.0333 (4)	
H8	0.550296	0.229713	0.072955	0.040*	
C9	0.65464 (18)	0.26575 (19)	0.03025 (12)	0.0399 (5)	
H9	0.640823	0.218576	−0.011255	0.048*	
C10	0.72683 (17)	0.3253 (2)	0.04103 (13)	0.0406 (5)	
H10	0.763934	0.318524	0.007670	0.049*	
C11	0.74731 (15)	0.39708 (19)	0.10124 (13)	0.0374 (5)	
C12	0.82271 (18)	0.4602 (2)	0.11175 (18)	0.0514 (6)	
H12	0.860130	0.451201	0.078903	0.062*	
C13	0.84281 (18)	0.5327 (3)	0.16696 (19)	0.0530 (6)	
H13	0.892498	0.575123	0.171845	0.064*	
C14	0.78832 (17)	0.5440 (2)	0.21732 (17)	0.0447 (5)	
H14	0.802454	0.593757	0.256962	0.054*	
C15	0.71539 (14)	0.48429 (17)	0.20974 (13)	0.0345 (4)	
H15	0.679896	0.494250	0.244118	0.041*	
C16	0.69124 (13)	0.40771 (16)	0.15184 (11)	0.0294 (4)	
C17	0.66524 (14)	0.23088 (18)	0.28874 (11)	0.0320 (4)	
C18	0.64028 (17)	0.13930 (19)	0.25149 (14)	0.0404 (5)	
H18	0.583392	0.132137	0.215185	0.048*	
C19	0.6984 (2)	0.0582 (2)	0.26725 (19)	0.0524 (7)	
H19	0.680885	−0.003562	0.241076	0.063*	
C20	0.7809 (2)	0.0665 (3)	0.3203 (2)	0.0636 (9)	
H20	0.819505	0.010366	0.331850	0.076*	
C21	0.8066 (2)	0.1569 (3)	0.35611 (19)	0.0645 (10)	
H21	0.863956	0.163296	0.391798	0.077*	
C22	0.74975 (16)	0.2403 (2)	0.34109 (14)	0.0453 (6)	
H22	0.768577	0.302392	0.366257	0.054*	
C23	0.54206 (12)	0.31305 (16)	0.33015 (10)	0.0278 (4)	
C24	0.50506 (13)	0.22578 (18)	0.34412 (11)	0.0313 (4)	
H24	0.519625	0.165058	0.322953	0.038*	
C25	0.44448 (13)	0.2244 (2)	0.39030 (11)	0.0341 (4)	
C26	0.40745 (17)	0.1344 (2)	0.40884 (14)	0.0434 (5)	
H26	0.423475	0.071736	0.391709	0.052*	
C27	0.34766 (18)	0.1393 (3)	0.45222 (15)	0.0509 (7)	
C28	0.32165 (16)	0.2313 (3)	0.47676 (13)	0.0517 (7)	
H28	0.279078	0.233155	0.505230	0.062*	
C29	0.35782 (15)	0.3182 (2)	0.45953 (12)	0.0433 (6)	
H29	0.339912	0.380171	0.476248	0.052*	
C30	0.42172 (13)	0.3177 (2)	0.41708 (11)	0.0350 (4)	
C31	0.51959 (13)	0.40074 (16)	0.36695 (10)	0.0297 (4)	
C32	0.5520 (2)	0.5719 (2)	0.40070 (17)	0.0498 (6)	
H32A	0.492849	0.598347	0.374896	0.075*	
H32B	0.596679	0.622211	0.399067	0.075*	
H32C	0.556169	0.555802	0.455161	0.075*	
C33	0.55723 (15)	0.71778 (17)	0.19319 (12)	0.0339 (4)	
C34	0.63133 (15)	0.76090 (18)	0.25665 (12)	0.0359 (4)	
H34	0.619022	0.768985	0.305370	0.043*	
C35	0.71187 (15)	0.78980 (18)	0.25582 (12)	0.0351 (4)	
H35	0.748219	0.813825	0.304432	0.042*	
C36	0.75467 (15)	0.79076 (17)	0.19113 (13)	0.0343 (4)	

### [4-(6-bromo-2-methoxyquinolin-3-yl)-3-hydroxy-3-(naphthalen-1-yl)-4-phenylbutyl]dimethylazanium 3-carboxyprop-2-enoate (desolvated_sq) Atomic displacement parameters (Å^2^)

**Table:**

	*U*^11^	*U*^22^	*U*^33^	*U*^12^	*U*^13^	*U*^23^
Br1	0.0858 (3)	0.1017 (3)	0.0933 (3)	−0.0388 (2)	0.0538 (2)	0.0023 (2)
O1	0.0269 (6)	0.0317 (7)	0.0303 (6)	−0.0008 (5)	0.0069 (5)	−0.0023 (5)
O2	0.0412 (8)	0.0359 (7)	0.0296 (7)	0.0055 (6)	0.0113 (6)	−0.0013 (6)
O3	0.0368 (8)	0.0462 (9)	0.0446 (8)	−0.0102 (7)	0.0180 (7)	−0.0101 (7)
O4	0.0447 (9)	0.0925 (16)	0.0338 (8)	−0.0314 (10)	0.0156 (7)	−0.0205 (9)
O5	0.0471 (10)	0.0827 (14)	0.0326 (8)	−0.0262 (10)	0.0185 (7)	−0.0094 (8)
O6	0.0304 (7)	0.0481 (9)	0.0533 (10)	−0.0050 (7)	0.0150 (7)	0.0025 (8)
N1	0.0229 (6)	0.0280 (7)	0.0253 (7)	0.0031 (5)	0.0072 (5)	0.0022 (5)
N2	0.0282 (8)	0.0520 (11)	0.0215 (7)	0.0112 (7)	0.0062 (6)	−0.0002 (7)
C1	0.0249 (8)	0.0346 (9)	0.0226 (7)	0.0061 (7)	0.0071 (6)	0.0028 (7)
C2	0.0240 (7)	0.0288 (8)	0.0211 (7)	0.0036 (6)	0.0064 (6)	0.0001 (6)
C3	0.0250 (8)	0.0306 (8)	0.0220 (7)	0.0070 (6)	0.0060 (6)	0.0014 (6)
C4	0.0280 (8)	0.0308 (8)	0.0229 (7)	0.0089 (7)	0.0066 (6)	0.0013 (6)
C5	0.0240 (8)	0.0438 (12)	0.0432 (11)	0.0043 (8)	0.0137 (8)	0.0108 (9)
C6	0.0331 (9)	0.0371 (10)	0.0299 (9)	0.0074 (8)	0.0072 (7)	0.0084 (8)
C7	0.0282 (8)	0.0311 (9)	0.0213 (7)	0.0091 (7)	0.0084 (6)	0.0029 (6)
C8	0.0372 (10)	0.0389 (10)	0.0235 (8)	0.0108 (8)	0.0082 (7)	−0.0007 (7)
C9	0.0530 (13)	0.0430 (11)	0.0255 (8)	0.0200 (10)	0.0142 (8)	0.0022 (8)
C10	0.0471 (12)	0.0477 (12)	0.0342 (10)	0.0225 (10)	0.0235 (9)	0.0124 (9)
C11	0.0343 (10)	0.0463 (12)	0.0368 (10)	0.0138 (9)	0.0184 (8)	0.0111 (9)
C12	0.0410 (12)	0.0619 (16)	0.0602 (15)	0.0081 (11)	0.0286 (11)	0.0153 (13)
C13	0.0360 (11)	0.0566 (16)	0.0704 (17)	−0.0023 (11)	0.0213 (11)	0.0092 (14)
C14	0.0355 (11)	0.0449 (13)	0.0524 (13)	−0.0048 (9)	0.0101 (9)	0.0017 (10)
C15	0.0315 (9)	0.0389 (10)	0.0334 (9)	0.0017 (8)	0.0098 (7)	0.0023 (8)
C16	0.0271 (8)	0.0355 (9)	0.0271 (8)	0.0070 (7)	0.0100 (7)	0.0054 (7)
C17	0.0293 (9)	0.0423 (10)	0.0270 (8)	0.0125 (8)	0.0122 (7)	0.0098 (8)
C18	0.0421 (12)	0.0399 (11)	0.0436 (11)	0.0151 (9)	0.0194 (9)	0.0108 (9)
C19	0.0627 (16)	0.0422 (13)	0.0624 (16)	0.0219 (12)	0.0341 (13)	0.0142 (12)
C20	0.0639 (18)	0.073 (2)	0.0621 (17)	0.0466 (17)	0.0304 (14)	0.0281 (16)
C21	0.0434 (14)	0.095 (3)	0.0527 (15)	0.0388 (16)	0.0102 (12)	0.0213 (16)
C22	0.0311 (10)	0.0676 (16)	0.0369 (10)	0.0193 (10)	0.0088 (8)	0.0091 (11)
C23	0.0237 (8)	0.0385 (10)	0.0207 (7)	0.0063 (7)	0.0053 (6)	0.0013 (7)
C24	0.0288 (8)	0.0418 (10)	0.0235 (7)	0.0032 (8)	0.0078 (6)	0.0025 (7)
C25	0.0250 (8)	0.0558 (13)	0.0210 (7)	0.0003 (8)	0.0056 (6)	0.0016 (8)
C26	0.0366 (11)	0.0622 (15)	0.0329 (10)	−0.0079 (11)	0.0121 (8)	0.0018 (10)
C27	0.0390 (12)	0.081 (2)	0.0347 (11)	−0.0167 (13)	0.0134 (9)	0.0007 (12)
C28	0.0309 (10)	0.098 (2)	0.0289 (9)	−0.0091 (13)	0.0121 (8)	−0.0074 (12)
C29	0.0285 (9)	0.0788 (18)	0.0234 (8)	0.0011 (10)	0.0085 (7)	−0.0066 (10)
C30	0.0226 (8)	0.0617 (14)	0.0191 (7)	0.0067 (8)	0.0034 (6)	0.0006 (8)
C31	0.0279 (8)	0.0398 (10)	0.0202 (7)	0.0079 (7)	0.0048 (6)	0.0020 (7)
C32	0.0591 (16)	0.0417 (12)	0.0522 (14)	0.0046 (12)	0.0217 (12)	−0.0106 (11)
C33	0.0352 (9)	0.0351 (10)	0.0331 (9)	−0.0075 (8)	0.0126 (8)	−0.0062 (8)
C34	0.0389 (10)	0.0424 (11)	0.0285 (8)	−0.0089 (9)	0.0129 (8)	−0.0067 (8)
C35	0.0335 (10)	0.0425 (11)	0.0286 (9)	−0.0053 (8)	0.0076 (7)	−0.0028 (8)
C36	0.0347 (10)	0.0338 (10)	0.0367 (10)	−0.0050 (8)	0.0135 (8)	0.0012 (8)

### [4-(6-bromo-2-methoxyquinolin-3-yl)-3-hydroxy-3-(naphthalen-1-yl)-4-phenylbutyl]dimethylazanium 3-carboxyprop-2-enoate (desolvated_sq) Geometric parameters (Å, º)

**Table:**

Br1—C27	1.897 (3)	C11—C12	1.425 (4)
O1—C2	1.428 (2)	C11—C16	1.439 (3)
O1—H1O	0.8400	C12—C13	1.353 (5)
O2—C31	1.352 (3)	C12—H12	0.9500
O2—C32	1.426 (3)	C13—C14	1.417 (4)
O3—C33	1.239 (3)	C13—H13	0.9500
O4—C33	1.276 (3)	C14—C15	1.373 (3)
O4—H5	1.42 (4)	C14—H14	0.9500
O5—C36	1.286 (3)	C15—C16	1.427 (3)
O5—H5	1.01 (4)	C15—H15	0.9500
O6—C36	1.230 (3)	C17—C18	1.395 (4)
N1—C6	1.485 (2)	C17—C22	1.398 (3)
N1—C5	1.489 (2)	C18—C19	1.394 (3)
N1—C4	1.496 (2)	C18—H18	0.9500
N1—H1	1.0000	C19—C20	1.378 (5)
N2—C31	1.302 (3)	C19—H19	0.9500
N2—C30	1.363 (3)	C20—C21	1.373 (6)
C1—C23	1.517 (3)	C20—H20	0.9500
C1—C17	1.520 (3)	C21—C22	1.406 (4)
C1—C2	1.582 (2)	C21—H21	0.9500
C1—H1A	1.0000	C22—H22	0.9500
C2—C3	1.542 (3)	C23—C24	1.358 (3)
C2—C7	1.542 (3)	C23—C31	1.436 (3)
C3—C4	1.529 (2)	C24—C25	1.428 (3)
C3—H3A	0.9900	C24—H24	0.9500
C3—H3B	0.9900	C25—C26	1.415 (4)
C4—H4A	0.9900	C25—C30	1.418 (4)
C4—H4B	0.9900	C26—C27	1.381 (4)
C5—H5A	0.9800	C26—H26	0.9500
C5—H5B	0.9800	C27—C28	1.405 (5)
C5—H5C	0.9800	C28—C29	1.365 (5)
C6—H6A	0.9800	C28—H28	0.9500
C6—H6B	0.9800	C29—C30	1.422 (3)
C6—H6C	0.9800	C29—H29	0.9500
C7—C8	1.380 (3)	C32—H32A	0.9800
C7—C16	1.439 (3)	C32—H32B	0.9800
C8—C9	1.418 (3)	C32—H32C	0.9800
C8—H8	0.9500	C33—C34	1.492 (3)
C9—C10	1.356 (4)	C34—C35	1.330 (3)
C9—H9	0.9500	C34—H34	0.9500
C10—C11	1.408 (4)	C35—C36	1.494 (3)
C10—H10	0.9500	C35—H35	0.9500
			
C2—O1—H1O	109.5	C13—C14—H14	119.5
C31—O2—C32	118.04 (19)	C14—C15—C16	122.1 (2)
C33—O4—H5	112.6 (16)	C14—C15—H15	119.0
C36—O5—H5	113 (2)	C16—C15—H15	119.0
C6—N1—C5	110.41 (15)	C15—C16—C7	125.18 (17)
C6—N1—C4	110.68 (15)	C15—C16—C11	116.2 (2)
C5—N1—C4	113.10 (16)	C7—C16—C11	118.64 (19)
C6—N1—H1	107.5	C18—C17—C22	118.9 (2)
C5—N1—H1	107.5	C18—C17—C1	123.96 (19)
C4—N1—H1	107.5	C22—C17—C1	117.0 (2)
C31—N2—C30	117.48 (19)	C19—C18—C17	120.3 (3)
C23—C1—C17	111.90 (16)	C19—C18—H18	119.8
C23—C1—C2	111.31 (15)	C17—C18—H18	119.8
C17—C1—C2	113.99 (15)	C20—C19—C18	120.9 (3)
C23—C1—H1A	106.3	C20—C19—H19	119.5
C17—C1—H1A	106.3	C18—C19—H19	119.5
C2—C1—H1A	106.3	C21—C20—C19	119.1 (2)
O1—C2—C3	107.91 (15)	C21—C20—H20	120.5
O1—C2—C7	107.43 (15)	C19—C20—H20	120.5
C3—C2—C7	112.34 (15)	C20—C21—C22	121.4 (3)
O1—C2—C1	108.89 (15)	C20—C21—H21	119.3
C3—C2—C1	110.23 (14)	C22—C21—H21	119.3
C7—C2—C1	109.92 (14)	C17—C22—C21	119.4 (3)
C4—C3—C2	112.22 (15)	C17—C22—H22	120.3
C4—C3—H3A	109.2	C21—C22—H22	120.3
C2—C3—H3A	109.2	C24—C23—C31	116.22 (18)
C4—C3—H3B	109.2	C24—C23—C1	123.66 (18)
C2—C3—H3B	109.2	C31—C23—C1	120.11 (19)
H3A—C3—H3B	107.9	C23—C24—C25	120.6 (2)
N1—C4—C3	111.81 (14)	C23—C24—H24	119.7
N1—C4—H4A	109.3	C25—C24—H24	119.7
C3—C4—H4A	109.3	C26—C25—C30	120.5 (2)
N1—C4—H4B	109.3	C26—C25—C24	122.3 (2)
C3—C4—H4B	109.3	C30—C25—C24	117.3 (2)
H4A—C4—H4B	107.9	C27—C26—C25	118.9 (3)
N1—C5—H5A	109.5	C27—C26—H26	120.5
N1—C5—H5B	109.5	C25—C26—H26	120.5
H5A—C5—H5B	109.5	C26—C27—C28	121.5 (3)
N1—C5—H5C	109.5	C26—C27—Br1	119.1 (3)
H5A—C5—H5C	109.5	C28—C27—Br1	119.43 (19)
H5B—C5—H5C	109.5	C29—C28—C27	119.7 (2)
N1—C6—H6A	109.5	C29—C28—H28	120.1
N1—C6—H6B	109.5	C27—C28—H28	120.1
H6A—C6—H6B	109.5	C28—C29—C30	121.4 (3)
N1—C6—H6C	109.5	C28—C29—H29	119.3
H6A—C6—H6C	109.5	C30—C29—H29	119.3
H6B—C6—H6C	109.5	N2—C30—C25	122.26 (18)
C8—C7—C16	118.65 (18)	N2—C30—C29	119.8 (2)
C8—C7—C2	117.80 (18)	C25—C30—C29	117.9 (2)
C16—C7—C2	123.55 (16)	N2—C31—O2	119.39 (19)
C7—C8—C9	121.8 (2)	N2—C31—C23	125.7 (2)
C7—C8—H8	119.1	O2—C31—C23	114.91 (17)
C9—C8—H8	119.1	O2—C32—H32A	109.5
C10—C9—C8	120.4 (2)	O2—C32—H32B	109.5
C10—C9—H9	119.8	H32A—C32—H32B	109.5
C8—C9—H9	119.8	O2—C32—H32C	109.5
C9—C10—C11	120.58 (19)	H32A—C32—H32C	109.5
C9—C10—H10	119.7	H32B—C32—H32C	109.5
C11—C10—H10	119.7	O3—C33—O4	122.5 (2)
C10—C11—C12	120.4 (2)	O3—C33—C34	117.47 (19)
C10—C11—C16	119.9 (2)	O4—C33—C34	120.0 (2)
C12—C11—C16	119.7 (2)	C35—C34—C33	130.2 (2)
C13—C12—C11	122.2 (2)	C35—C34—H34	114.9
C13—C12—H12	118.9	C33—C34—H34	114.9
C11—C12—H12	118.9	C34—C35—C36	130.7 (2)
C12—C13—C14	118.7 (3)	C34—C35—H35	114.6
C12—C13—H13	120.6	C36—C35—H35	114.6
C14—C13—H13	120.6	O6—C36—O5	122.4 (2)
C15—C14—C13	121.0 (3)	O6—C36—C35	117.5 (2)
C15—C14—H14	119.5	O5—C36—C35	119.99 (19)
			
C23—C1—C2—O1	−57.1 (2)	C1—C17—C18—C19	−175.8 (2)
C17—C1—C2—O1	70.6 (2)	C17—C18—C19—C20	0.7 (4)
C23—C1—C2—C3	61.1 (2)	C18—C19—C20—C21	−1.9 (5)
C17—C1—C2—C3	−171.18 (17)	C19—C20—C21—C22	1.4 (5)
C23—C1—C2—C7	−174.55 (16)	C18—C17—C22—C21	−1.4 (4)
C17—C1—C2—C7	−46.8 (2)	C1—C17—C22—C21	175.6 (2)
O1—C2—C3—C4	−65.28 (19)	C20—C21—C22—C17	0.2 (4)
C7—C2—C3—C4	53.0 (2)	C17—C1—C23—C24	−37.0 (2)
C1—C2—C3—C4	175.92 (16)	C2—C1—C23—C24	91.9 (2)
C6—N1—C4—C3	163.50 (17)	C17—C1—C23—C31	142.75 (18)
C5—N1—C4—C3	−72.0 (2)	C2—C1—C23—C31	−88.4 (2)
C2—C3—C4—N1	169.68 (16)	C31—C23—C24—C25	2.6 (3)
O1—C2—C7—C8	0.3 (2)	C1—C23—C24—C25	−177.69 (17)
C3—C2—C7—C8	−118.21 (19)	C23—C24—C25—C26	−177.30 (19)
C1—C2—C7—C8	118.66 (18)	C23—C24—C25—C30	3.1 (3)
O1—C2—C7—C16	−178.92 (16)	C30—C25—C26—C27	1.1 (3)
C3—C2—C7—C16	62.6 (2)	C24—C25—C26—C27	−178.4 (2)
C1—C2—C7—C16	−60.6 (2)	C25—C26—C27—C28	1.3 (4)
C16—C7—C8—C9	−0.8 (3)	C25—C26—C27—Br1	−177.66 (17)
C2—C7—C8—C9	179.92 (18)	C26—C27—C28—C29	−1.9 (4)
C7—C8—C9—C10	1.8 (3)	Br1—C27—C28—C29	177.10 (18)
C8—C9—C10—C11	−1.3 (3)	C27—C28—C29—C30	−0.1 (3)
C9—C10—C11—C12	−179.3 (2)	C31—N2—C30—C25	1.2 (3)
C9—C10—C11—C16	−0.1 (3)	C31—N2—C30—C29	179.20 (18)
C10—C11—C12—C13	177.4 (3)	C26—C25—C30—N2	175.10 (19)
C16—C11—C12—C13	−1.8 (4)	C24—C25—C30—N2	−5.3 (3)
C11—C12—C13—C14	1.8 (4)	C26—C25—C30—C29	−3.0 (3)
C12—C13—C14—C15	−1.2 (4)	C24—C25—C30—C29	176.64 (18)
C13—C14—C15—C16	0.6 (4)	C28—C29—C30—N2	−175.7 (2)
C14—C15—C16—C7	−179.7 (2)	C28—C29—C30—C25	2.4 (3)
C14—C15—C16—C11	−0.5 (3)	C30—N2—C31—O2	−172.83 (16)
C8—C7—C16—C15	178.55 (19)	C30—N2—C31—C23	5.4 (3)
C2—C7—C16—C15	−2.2 (3)	C32—O2—C31—N2	2.3 (3)
C8—C7—C16—C11	−0.6 (3)	C32—O2—C31—C23	−176.2 (2)
C2—C7—C16—C11	178.63 (17)	C24—C23—C31—N2	−7.4 (3)
C10—C11—C16—C15	−178.2 (2)	C1—C23—C31—N2	172.90 (17)
C12—C11—C16—C15	1.0 (3)	C24—C23—C31—O2	170.95 (16)
C10—C11—C16—C7	1.1 (3)	C1—C23—C31—O2	−8.8 (2)
C12—C11—C16—C7	−179.7 (2)	O3—C33—C34—C35	175.4 (3)
C23—C1—C17—C18	80.5 (2)	O4—C33—C34—C35	−2.2 (4)
C2—C1—C17—C18	−46.9 (3)	C33—C34—C35—C36	1.3 (5)
C23—C1—C17—C22	−96.3 (2)	C34—C35—C36—O6	−175.7 (3)
C2—C1—C17—C22	136.30 (19)	C34—C35—C36—O5	5.8 (4)
C22—C17—C18—C19	0.9 (3)		

### [4-(6-bromo-2-methoxyquinolin-3-yl)-3-hydroxy-3-(naphthalen-1-yl)-4-phenylbutyl]dimethylazanium 3-carboxyprop-2-enoate (desolvated_sq) Hydrogen-bond geometry (Å, º)

**Table:**

*D*—H···*A*	*D*—H	H···*A*	*D*···*A*	*D*—H···*A*
O1—H1*O*···O6^i^	0.84	1.98	2.820 (2)	180
O5—H5···O4	1.01 (4)	1.42 (4)	2.420 (3)	169 (4)
N1—H1···O3	1.00	1.71	2.705 (2)	172
N1—H1···O4	1.00	2.51	3.186 (2)	125

Symmetry code: (i) *x*−1/2, *y*−1/2, *z*.

## References

[bb1] Brigden, G., Hewison, C. & Varaine, F. (2015). *Infect. Drug Resist.* **8**, 367–378. 10.2147/IDR.S68351 PMC4634826 26586956

[bb2] Bruker (2020). *APEX3* and *SAINT.* Bruker Nano Inc., Madison, Wisconsin, USA.

[bb3] Etter, M. C., MacDonald, J. C. & Bernstein, J. (1990). *Acta Cryst.* B**46**, 256–262. 10.1107/s0108768189012929 2344397

[bb4] European Chemical Agency (2015). Maleic Acid (CAS 110-16-7). Registered Substances Dossier. European Chemical Agency. Available from http://echa.europa.eu/

[bb5] Groom, C. R., Bruno, I. J., Lightfoot, M. P. & Ward, S. C. (2016). *Acta Cryst.* B**72**, 171–179. 10.1107/S2052520616003954 PMC4822653 27048719

[bb6] Hegyi, J. F. A. L., Aelterman, W. A. A., Lang, Y. L., Stokbroekx, S. C. M., Leys, C., Van Remoortere, P. J. M. & Faure, A. (2013). United States Patent US 8,546,428, Janssen Pharmaceuticals, USA.

[bb7] Hübschle, C. B., Sheldrick, G. M. & Dittrich, B. (2011). *J. Appl. Cryst.* **44**, 1281–1284. 10.1107/S0021889811043202 PMC3246833 22477785

[bb8] Krause, L., Herbst-Irmer, R., Sheldrick, G. M. & Stalke, D. (2015). *J. Appl. Cryst.* **48**, 3–10. 10.1107/S1600576714022985 PMC4453166 26089746

[bb9] Macrae, C. F., Sovago, I., Cottrell, S. J., Galek, P. T. A., McCabe, P., Pidcock, E., Platings, M., Shields, G. P., Stevens, J. S., Towler, M. & Wood, P. A. (2020). *J. Appl. Cryst.* **53**, 226–235. 10.1107/S1600576719014092 PMC6998782 32047413

[bb10] Okezue, M., Smith, D., Zeller, M., Byrn, S. R., Smith, P., Bogandowich-Knipp, S., Purcell, D. K. & Clase, K. L. (2020). *Acta Cryst.* C**76**, 1010–1023. 10.1107/S2053229620013455 PMC7642769 33148877

[bb11] Parsons, S., Flack, H. D. & Wagner, T. (2013). *Acta Cryst.* B**69**, 249–259. 10.1107/S2052519213010014 PMC3661305 23719469

[bb12] Petit, S., Coquerel, G., Meyer, C. & Guillemont, J. (2007). *J. Mol. Struct.* **837**, 252–256.

[bb19] Rombouts, J. A., Veenboer, R. P., Villellas, C., Lu, P., Ehlers, A. W., Andries, K., Koul, A., Lill, H., Ruijter, E., Orru, R. V. A., Lammertsma, K., Bald, D. & Slootweg, J. C. (2016). *RSC Adv.* **6**, 108708–108716.

[bb13] Sheldrick, G. M. (2008). *Acta Cryst.* A**64**, 112–122. 10.1107/S0108767307043930 18156677

[bb14] Sheldrick, G. M. (2015*a*). *Acta Cryst.* A**71**, 3–8.

[bb15] Sheldrick, G. M. (2015*b*). *Acta Cryst.* C**71**, 3–8.

[bb16] Sluis, P. van der & Spek, A. L. (1990). *Acta Cryst.* A**46**, 194–201.

[bb17] Spek, A. L. (2015). *Acta Cryst.* C**71**, 9–18. 10.1107/S2053229614024929 25567569

[bb18] Westrip, S. P. (2010). *J. Appl. Cryst.* **43**, 920–925.

