# Supplementary material for: Maleate salts of bedaquiline
Source: Acta Crystallogr E Crystallogr Commun. 2021 Mar 26;77(Pt 4):433–45. doi: 10.1107/S2056989021002991 (PMC8025853; doi:10.1107/S2056989021002991)
Supplement: Supplementary file 2 [file e-77-00433-sup8.pdf]

Maleate Salts of Bedaquiline

Matthias Zeller[a*], Susan Bogdanowich-Knipp[b], Pamela Smith[c, d], Dale Purcell[e], Mercy Okezue[f], Daniel Smith[f], Stephen R. Byrn[b, f], Kari Clase[f,g]

[a]Purdue University, Chemistry, 560 Oval Dr., West Lafayette, IN 47907-2084, USA, [b]Ravine Pharma LLC, 3425 DuBois St., West Lafayette, IN 47906, A, [c]Improved Pharma LLC, 1281 Win Hentschel Blvd. Suite 1565, West Lafayette, IN 47906, [d]Leading with Smart Science, LLC, 5315 Shootingstar Ln, West Lafayette, IN 47906, [e]Chemical Microscopy LLC, 1281 Win Hentschel Blvd., West Lafayette, IN 47906, [f]Purdue University, Industrial and Physical Pharmacy, 575 Stadium Mall, West Lafayette, IN 47906, US, [g]Biotechnology Innovation and Regulatory Science Center, Lilly Hall of Life Sciences, 915 State Street, Purdue University, West Lafayette, IN 47906.*Correspondence email: zeller4@purdue.edu.

Supporting Information

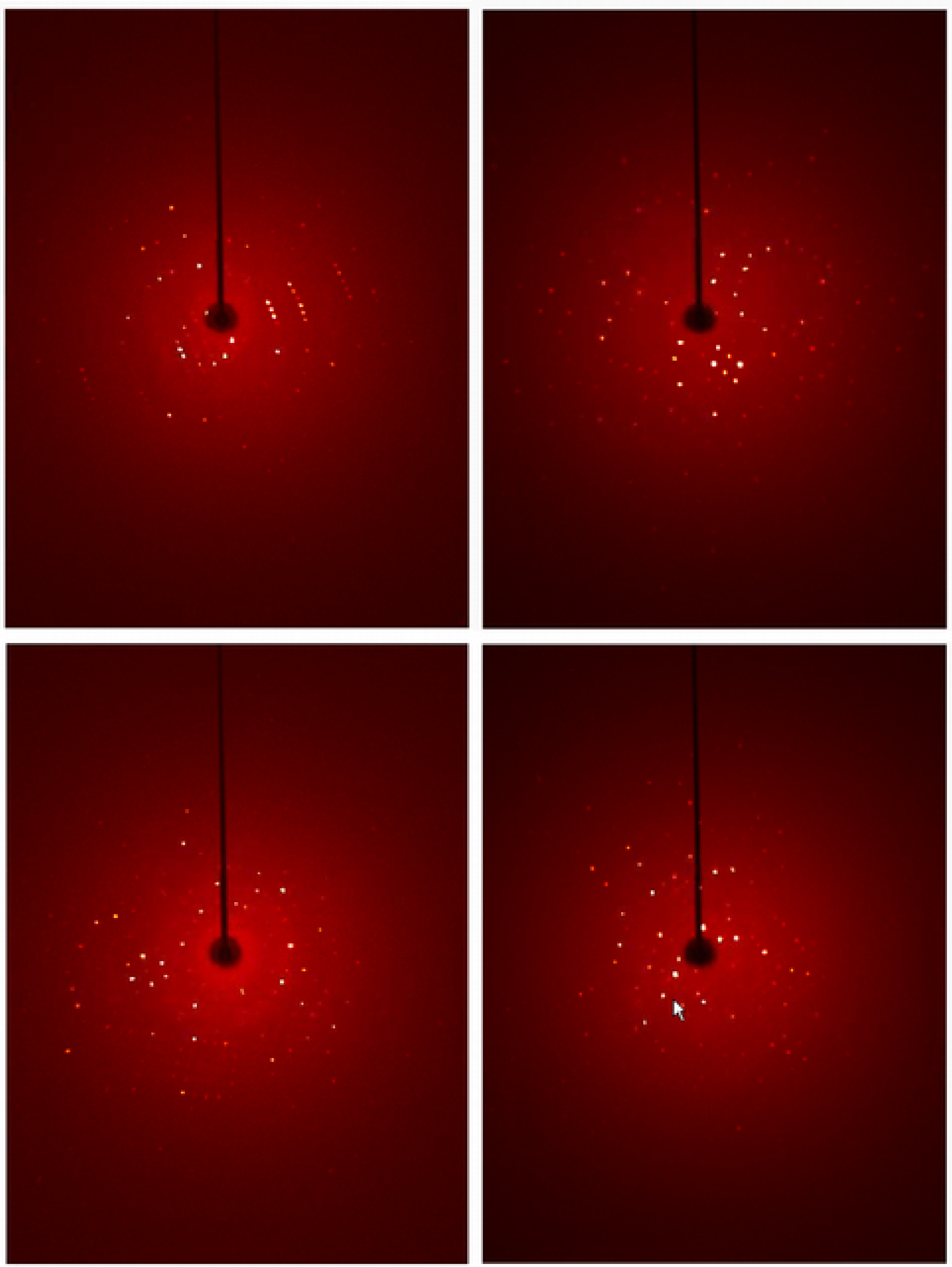

Figure S1. Comparison of diffraction patterns from crystals fresh out of mother liquor and after storing in atmosphere for 14 hrs. Patterns are from different but similar sized crystals. Top left: acetone/hexane solvate, native. Top right: acetone/hexane solvate, after 14hrs. Bottom left: ethyl acetate solvate, native. Bottom right: ethyl acetate, after 14hrs.

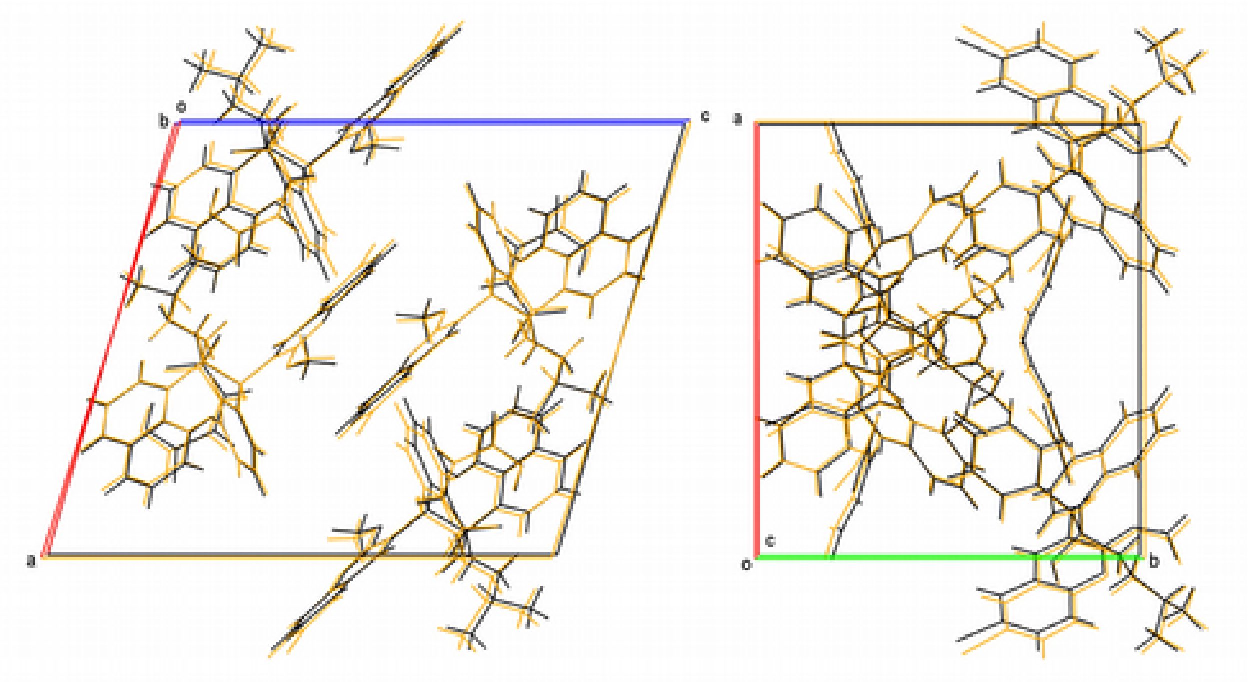

Figure S2. Unit cell overlay of the acetone/hexane solvate (orange, solvate molecules omitted for clarity) and the structure of the desolvated structure (black, derived from the acetone/hexane solvate).

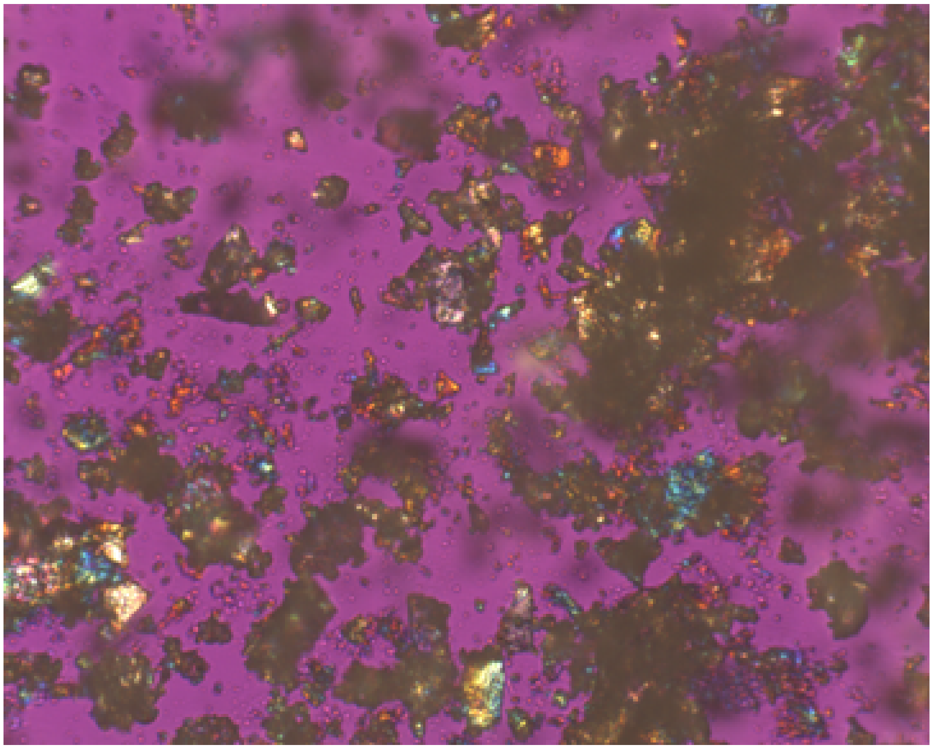

Figure S3: Bedaquiline maleate (n-hexanes/acetone) Run 1, Image file: 1-91-2-001, Temperature: 32.3 °C, start of heating.

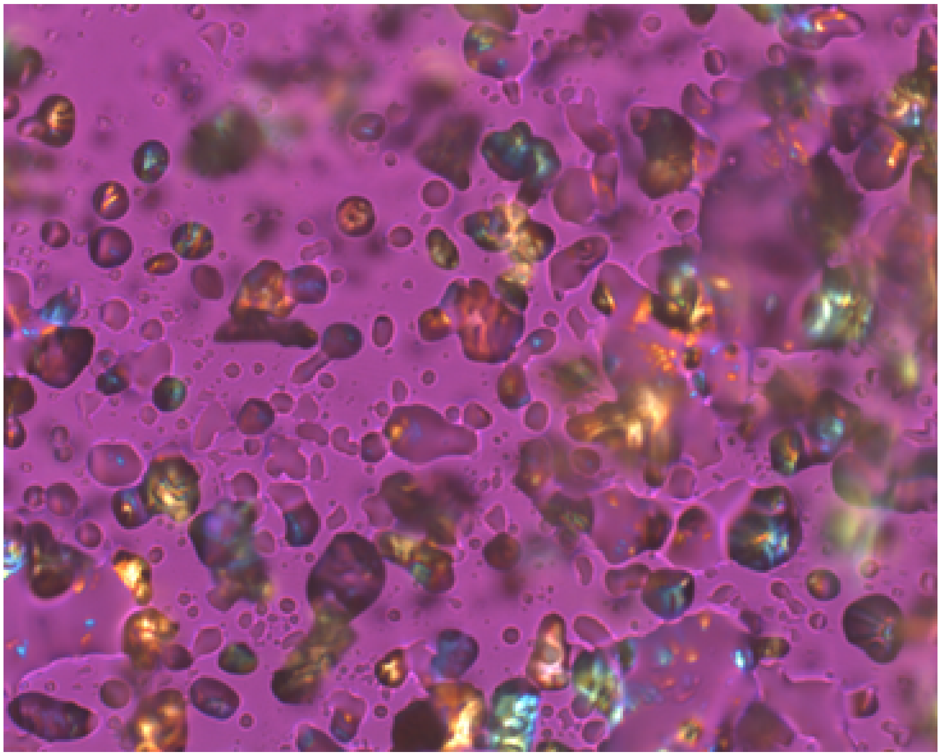

Figure S4: Bedaquiline maleate (n-hexanes/acetone) Run 1, Image file: 1-91-2-003, Temperature: 130.6 °C, melting.

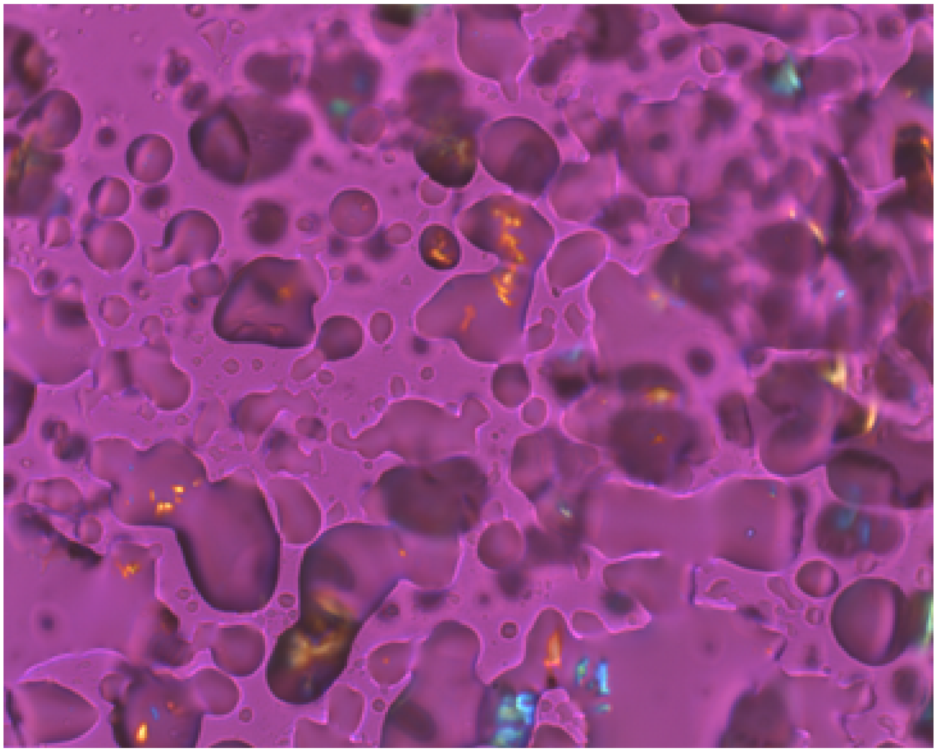

Figure S5: Bedaquiline maleate (n-hexanes/acetone) Run 1, Image file: 1-91-2-004, Temperature: 131.5 °C, melting.

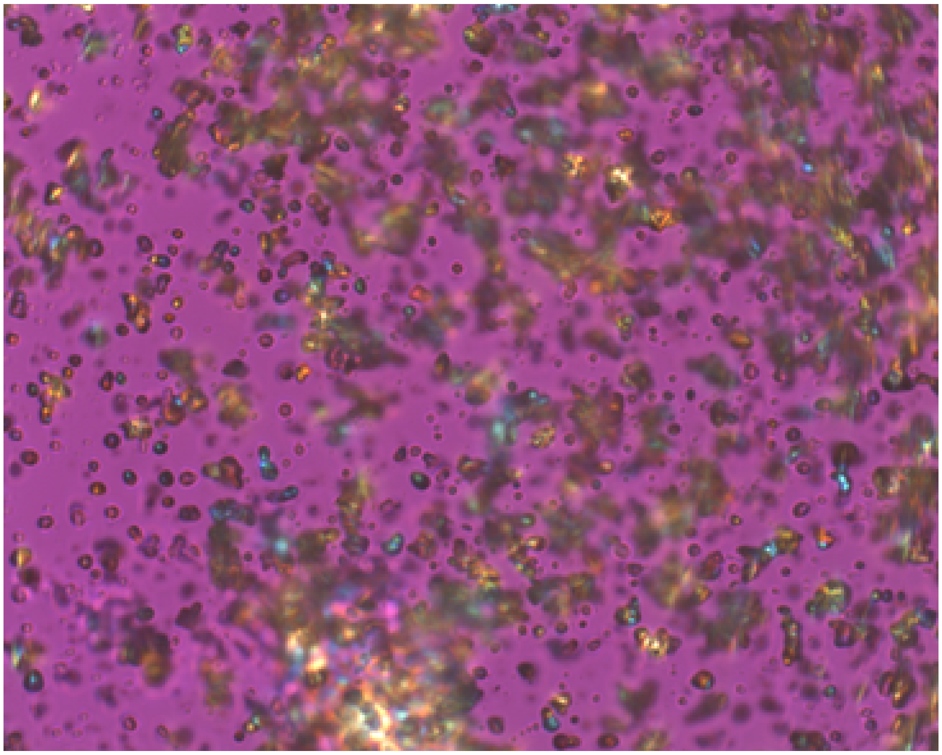

Figure S6: Bedaquiline maleate (n-hexanes/acetone) Run 2, Image file: 1-91-2-008, Temperature: 124.7 °C, melting.

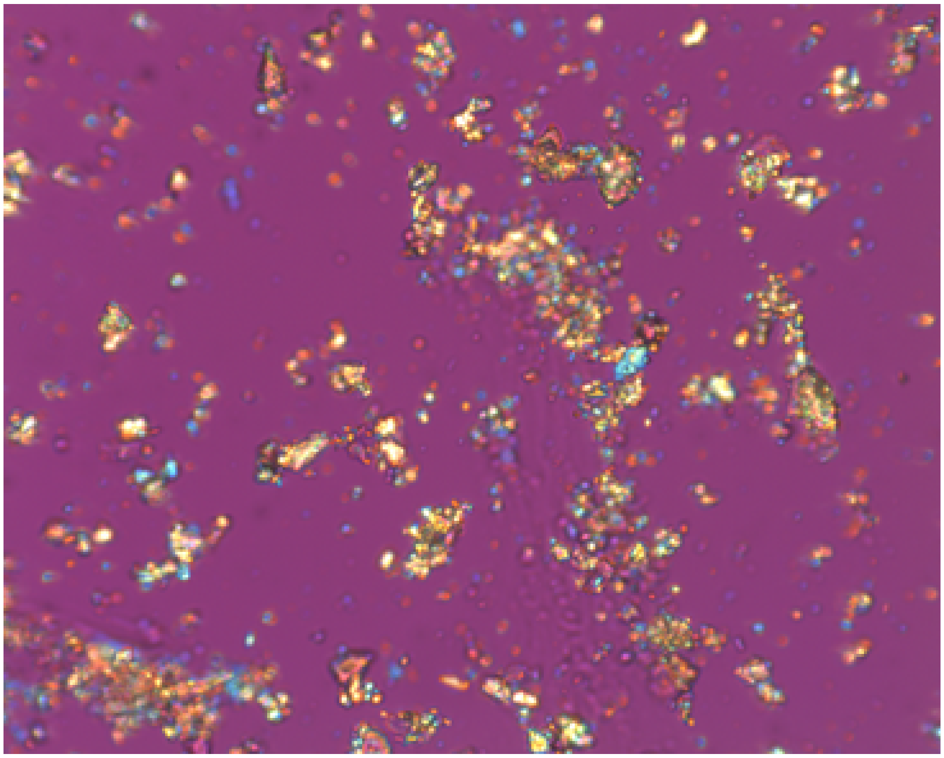

Figure S7: Bedaquiline maleate (n-hexanes/acetone) Run 3, Image file: 1-91-2-012, Temperature: 36.2 °C, start of heating.

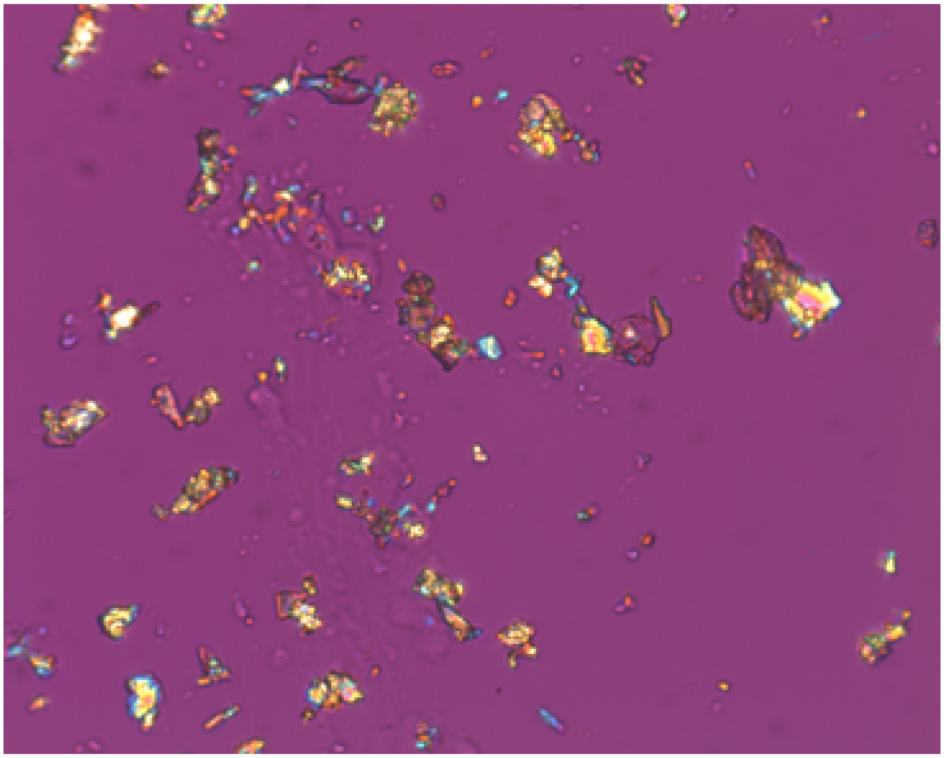

Figure S8: Bedaquiline maleate (n-hexanes/acetone) Run 3, Image file: 1-91-2-013, Temperature: 119.9 °C, onset melting.
